# RADICL-seq identifies general and cell type–specific principles of genome-wide RNA-chromatin interactions

**DOI:** 10.1038/s41467-020-14337-6

**Published:** 2020-02-24

**Authors:** Alessandro Bonetti, Federico Agostini, Ana Maria Suzuki, Kosuke Hashimoto, Giovanni Pascarella, Juliette Gimenez, Leonie Roos, Alex J. Nash, Marco Ghilotti, Christopher J. F. Cameron, Matthew Valentine, Yulia A. Medvedeva, Shuhei Noguchi, Eneritz Agirre, Kaori Kashi, Samudyata, Joachim Luginbühl, Riccardo Cazzoli, Saumya Agrawal, Nicholas M. Luscombe, Mathieu Blanchette, Takeya Kasukawa, Michiel de Hoon, Erik Arner, Boris Lenhard, Charles Plessy, Gonçalo Castelo-Branco, Valerio Orlando, Piero Carninci

**Affiliations:** 1 RIKEN Center for Integrative Medical Sciences, Yokohama, Kanagawa 230-0045 Japan; 2 Laboratory of Molecular Neurobiology, Department Medical Biochemistry and Biophysics, Karolinska Institutet, Stockholm, Sweden; 3 The Francis Crick Institute, 1 Midland Road, London, NW1 1AT UK; 4 Department of Medicine (H7), Karolinska Institutet, Stockholm, 141 86 Sweden; 5 Epigenetics and Genome Reprogramming Laboratory, IRCCS Fondazione Santa Lucia, Rome, Italy; 6 Faculty of Medicine, Imperial College London, Institute of Clinical Sciences, London, W12 0NN UK; 7 MRC London Institute of Medical Sciences, London, W12 0NN UK; 8 School of Computer Science, McGill University, Montréal, QC Canada; 9 Department of Biochemistry and Goodman Cancer Research Centre, McGill University, Montréal, QC Canada; 10 Institute of Bioengineering, Research Centre of Biotechnology, Russian Academy of Science, 117312 Moscow, Russia; 11 Department of Computational Biology, Vavilov Institute of General Genetics, Russian Academy of Science, 119991 Moscow, Russia; 12 Department of Biological and Medical Physics, Moscow Institute of Physics and Technology, 141701 Dolgoprudny, Moscow Region Russia; 13 Department of Experimental Oncology, IEO, European Institute of Oncology IRCCS, Milan, Italy; 14 UCL Genetics Institute, University College London, London, WC1E 6BT UK; 15 Okinawa Institute of Science and Technology, Graduate University, 1919-1 Tancha, Onna-son, Kunigami-gun, Okinawa, 904-0495 Japan; 16 Sars International Centre for Marine Molecular Biology, University of Bergen, 5008 Bergen, Norway; 17 KAUST Environmental Epigenetics Program, King Abdullah University of Science and Technology (KAUST), Division of Biological Environmental Sciences and Engineering, 23955-6900 Thuwal, Saudi Arabia

**Keywords:** Biological techniques, Chromatin

## Abstract

Mammalian genomes encode tens of thousands of noncoding RNAs. Most noncoding transcripts exhibit nuclear localization and several have been shown to play a role in the regulation of gene expression and chromatin remodeling. To investigate the function of such RNAs, methods to massively map the genomic interacting sites of multiple transcripts have been developed; however, these methods have some limitations. Here, we introduce RNA And DNA Interacting Complexes Ligated and sequenced (RADICL-seq), a technology that maps genome-wide RNA–chromatin interactions in intact nuclei. RADICL-seq is a proximity ligation-based methodology that reduces the bias for nascent transcription, while increasing genomic coverage and unique mapping rate efficiency compared with existing methods. RADICL-seq identifies distinct patterns of genome occupancy for different classes of transcripts as well as cell type–specific RNA-chromatin interactions, and highlights the role of transcription in the establishment of chromatin structure.

## Abstract (web summary)

Mammalian genomes encode tens of thousands of ncRNAs that have important roles in regulation of gene expression and chromatin organization. Here, the authors present RADICLseq to map RNA-chromatin interactions in intact nuclei to shed light on these fine-tuned processes.

## Introduction

The vast majority of mammalian genomes is pervasively transcribed, accounting for a previously unappreciated complexity of the noncoding RNA (ncRNA) fraction^1^. In particular, long ncRNAs (lncRNAs) have emerged as important regulators of various biological processes^2^. Although most lncRNAs exhibit nuclear localization with enrichment for the chromatin fraction, the genomic-interacting regions for most of these transcripts are still unknown^2,3^.

Several technologies have been developed to map the genomic-interacting sites of lncRNAs^4–6^. However, these methodologies rely on the use of antisense probes to target individual transcripts and are not suitable for de novo discovery and high-throughput application in multiple cell types.

A few technologies have emerged to assess genome-wide RNA–chromatin interactions^7–9^, but each has limitations. Mapping RNA-genome interactions (MARGI) is a proximity ligation-based technology that requires a high number of input cells (i.e., hundreds of millions) and the disruption of the nuclear structure^7^, which can result in detection of a large number of spurious interactions^10^; because of this, it has limited applicability to investigations of RNA–chromatin interactions in multiple cell types. Chromatin-associated RNA-sequencing (CHAR-seq) and global RNA interaction with DNA by deep-sequencing (GRID-seq) utilize in situ approaches to detect genome-wide RNA–chromatin contacts^8,9^. CHAR-seq requires a large number of cells as starting material and the use of *Dpn*II to digest the chromatin. Because *Dpn*II has a restricted number of sites across the genome, this method has limited coverage of captured RNA–DNA interactions^9^. In addition, the technology does not size select the molecules containing both interacting RNA and DNA, resulting in a large fraction of uninformative sequences in the final library. GRID-seq preferentially captures nascent RNA–chromatin interactions and, consequently, may overlook the presence of other patterns of genome occupancy by specific classes of transcripts. Furthermore, its reliance on a restriction enzyme for chromatin fragmentation coupled with read length restricted to 20 nucleotides (nt) limits both genome coverage and mappability.

To address these limitations, we introduce RNA And DNA-Interacting Complexes Ligated and sequenced (RADICL-seq), a methodology to identify genome-wide RNA–chromatin interactions in crosslinked nuclei that substantially improves on previously published methods. Specifically, RADICL-seq reduces the bias for nascent transcription while increasing genomic coverage and unique mapping rate efficiency. Application of RADICL-seq to mouse embryonic stem cells (mESCs) and mouse oligodendrocyte progenitor cells (mOPCs) reveals distinct genome occupancy patterns for specific classes of transcripts and uncovers cell-type-specific RNA–chromatin interactions. Furthermore, our results highlight the role of transcription in the establishment of the three-dimensional (3D) structure of chromatin.

## Results

### RADICL-seq technology

**Fig. 1:**
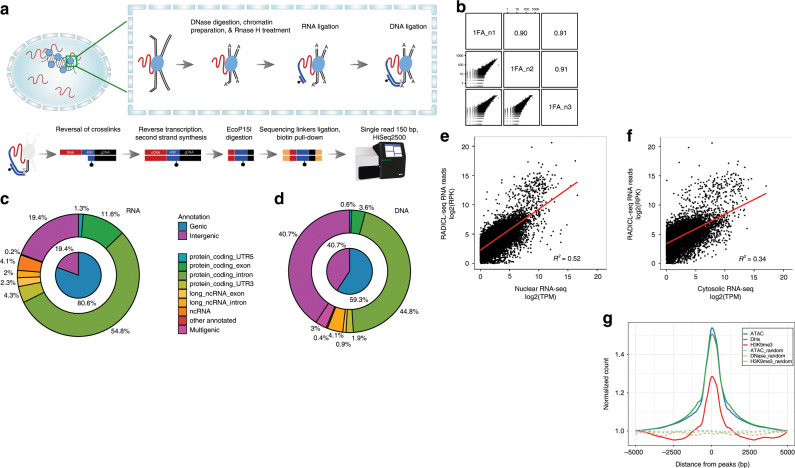
RADICL-seq method for the identification of RNA–chromatin interactions. **a** Schematic representation of the RADICL-seq protocol. Top: sequence of enzymatic reactions occurring in fixed nuclei after partial lysis of the nuclear membrane. The adduct formed by genomic DNA (black), RNA (red), and proteins (blue circles) is subjected to controlled DNase I digestion and chromatin preparation. After RNase H digestion, an adapter (dark blue) containing an internally biotinylated residue (black dot) bridges the RNA and DNA molecules that lie in close proximity. Bottom: sequence of enzymatic reactions performed in solution. After reversal of crosslinks, the RNA–DNA chimera is converted into a fully double-stranded DNA (dsDNA) molecule and digested by the *Eco*P15I enzyme to a designated length (adpr, adapter). After ligation of the sequencing linkers (yellow) and biotin pull-down, the library is PCR amplified and high-throughput sequenced. **b** Reproducibility of the RNA–DNA interaction frequencies across replicates, assessed by counting the occurrences of genic transcripts and 25-kb genomic bin pairs. **c** RNA and **d** DNA tag origins. The inner pie charts represent a broader classification into intergenic and genic (annotated genes), while the outer circles show a finer classification of the genic portion. **e**, **f** Comparison between (**e**) nuclear and (**f**) cytosolic RNA-seq tag counts and RADICL-seq genic transcript counts. The former are normalized to tags per million (TPM), while the latter are normalized to reads per kilobase (RPK). The linear regression lines are shown in red. **g** Density of the normalized counts of DNA reads detected by RADICL-seq around ATAC-seq (blue), DHS-seq (green), and H3K9me3 ChIP-seq (red) peaks; dashed lines represent the density profiles of aggregated signal from random genomic reads equal in number and size to the real peaks.

We developed RADICL-seq by using R08, a male mESC line with a deeply characterized transcriptome^11^, to identify genome-wide RNA–chromatin (or RNA–DNA) interactions in preserved nuclei (Fig. 1a). We crosslinked cells with 1% formaldehyde (FA) unless stated otherwise. After crosslinking we isolated the nuclei, partially digested the genomic DNA with DNase I, and end-prepared the chromatin. During technical development of RADICL-seq, we evaluated different enzymes that specifically act on RNA to generate a 3ʹ-hydroxyl end compatible with RNA ligation (Supplementary Fig. 1a). Sequencing data of test RADICL-seq libraries showed that RNase H treatment increased the percentage of uniquely mapped RNA–chromatin interactions by decreasing the ribosomal RNA (rRNA) content, when compared with nuclease S1 or RNase V1 treatment, or no treatment. RNase H is known to target RNA–DNA hybrids and, therefore, it could potentially digest nascent RNA bound to its transcription locus, including the highly transcribed rRNA. Indeed, we observed a 1.7-fold reduction in the number of RNA–DNA interactions occurring at a distance of <1 kb between RNase H-treated and -untreated samples (Supplementary Fig. 1b).

After enzymatic treatment of the RNA, we introduced a bridge adapter to specifically ligate proximal RNA and DNA (Supplementary Fig. 1c). The adapter is a 5ʹ pre-adenylated, partially double-stranded DNA linker with an internal biotin moiety and a thymidine (T) overhang located at the 3ʹ end. The adapter was selectively ligated to available 3ʹ-OH RNA ends, and the excess of non-ligated adapter was washed away before DNA ligation was performed to capture the digested genomic DNA ends located in near proximity (Fig. 1a). The experimental design of RADICL-seq not only allows for unambiguous discrimination of RNA and DNA tags within the chimeric construct but also correctly assigns sense and antisense transcripts by retaining the information on the RNA fragment strand. After reversal of crosslinks, the resulting RNA–adapter–DNA chimera was converted to double-stranded DNA by reverse transcription and second-strand DNA synthesis, followed by digestion with the type III restriction enzyme *Eco*P15I, which cleaves 25 to 27 nucleotides (nts) away from each of its two recognition sites strategically placed within the adapter (Supplementary Fig. 1c). Next, the digested DNA fragments were end-prepared and ligated to sequencing linkers. Finally, the biotinylated adapter-ligated molecules were captured and PCR amplified, and the library corresponding to the correct RNA–adapter–DNA ligation product size was gel purified (Supplementary Fig. 1d).

To test whether the captured interactions were dependent on the amount of crosslinking agent, we compared results for 1 and 2% FA (1FA and 2FA) datasets. After deep sequencing, RADICL-seq produced an average of 120 and 115 million 150-nt single-end raw reads from 1FA and 2FA libraries, respectively (Supplementary Fig. 2a). Each library with the two different crosslinking conditions yielded over 15 million RNA–DNA pairs uniquely mapping to the reference genome (Supplementary Fig. 2a). We prepared libraries from three biological replicates for each experimental condition. RADICL-seq exhibited high reproducibility among biological replicates and conditions, even when crosslinked with different FA concentrations (Fig. 1b and Supplementary Fig. 2b). Since results obtained with 1 and 2% FA crosslinking were highly comparable, all analyses described below were conducted with 1FA unless stated otherwise.

To characterize the interactions detected by RADICL-seq, we annotated RNA–DNA pairs that could be uniquely mapped to the genome. The RNA tags were found to be primarily from genic regions with a dominant contribution from intronic reads (Fig. 1c). In contrast, DNA tags had an equivalent contribution from genic (mainly intronic) and intergenic regions (Fig. 1d). When the distributions of RNA and DNA tags captured by RADICL-seq were compared with the background distribution, we observed an enrichment for regions of the genome that have functional annotations (Supplementary Fig. 2c). We assigned the RNA and DNA fragments captured by RADICL-seq to the genomic features annotated by the GENCODE consortium^12^ and analyzed their distribution among different classes of gene biotypes (Supplementary Fig. 2d). Protein-coding genes were the most abundant class of loci detected by RADICL-seq at both the RNA and DNA level. Indeed, we observed multiple classes of transcripts interacting with chromatin regions encompassing protein-coding genes, suggesting a multi-layered regulation for the expression of these mRNAs.

When the expression of chromatin-interacting RNAs was compared with fractionated RNA-seq data^11^, higher correlation was found with the nuclear fraction than the cytosolic counterpart (Fig. 1e, f). This finding is consistent with RADICL-seq capturing ligation events occurring between RNAs and DNAs located within intact nuclei.

Although the majority of DNA reads captured by RADICL-seq originated from euchromatin (based on DNase I hypersensitive site sequencing (DHS-seq) and assay for transposase-accessible chromatin using sequencing *(*ATAC-seq) data), we observed a minor enrichment from genomic regions located in heterochromatic regions, consistent with the role of some lncRNAs as repressors of gene expression^13^ (Fig. 1g).

To better evaluate the quality of our results, we developed two controls (Supplementary Fig. 3a). The first control was used to test the stability of RNA–chromatin interactions upon transcriptional blockade. Hence, we treated mESCs with actinomycin D (ActD), an inhibitor of RNA polymerase II (RNA pol II) elongation^14^, for 4 h before crosslinking with 1% FA (Supplementary Fig. 3b, c). The second control was developed to estimate the specificity of RNA–chromatin interactions mediated by the presence of proteins. To this end, 1% FA crosslinks were reversed immediately before the RNA ligation reaction by digesting the sample with proteinase K in denaturing conditions. As a result, RNA and DNA would be able to reproducibly interact only if the binding was direct and not mediated by the presence of proteins. We defined this dataset as “non-protein mediated” (NPM) (Supplementary Fig. 3d, e). Since the standard 1% FA crosslinking condition includes both protein-mediated and NPM interactions, we defined the 1FA dataset as “total.” Although total and ActD-treated datasets displayed a relative similarity in the distribution of their RNA–DNA interactions, the two datasets greatly differed from the NPM dataset (Supplementary Fig. 4).

### Comparison of RADICL-seq with existing technologies

RADICL-seq introduces substantial improvements over similar RNA–chromatin proximity ligation approaches^7–9^. Compared with MARGI, RADICL-seq minimizes the frequency of spurious interactions in the dataset by performing the in situ ligation in intact nuclei. Moreover, RADICL-seq requires a substantially lower number of cells (two million) than MARGI or CHAR-seq, which require 400 and 100 million cells, respectively.

**Fig. 2:**
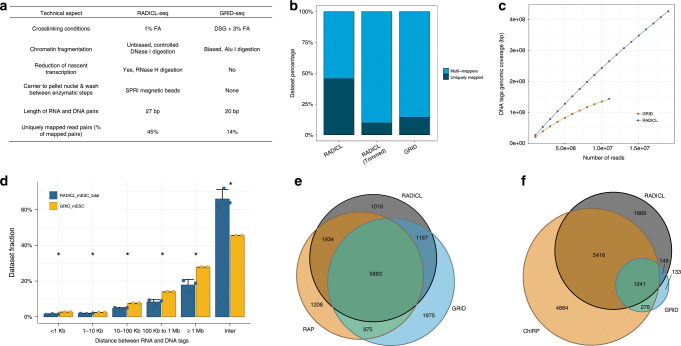
Comparison of RADICL-seq with similar methods. **a** Summary of the features that distinguish RADICL-seq from GRID-seq. **b** Analysis of the read length and mapping outcome. Unique (dark gray) and multi-mapping (blue) reads are reported as percentage of the total number of reads pool. RADICL-seq reads were artificially trimmed down to 20 nt for direct comparison with the GRID-seq dataset. **c** Assessment of the genomic coverage as a function of the sequencing depth for RADICL-seq (blue) and GRID-seq (yellow). The coverage was calculated for both datasets by sub-sampling with a step of 1,000,000 reads up to the maximum available number of reads. **d** Distribution of the linear genomic distance between RNA and DNA tags derived from the same read for the GRID-seq (yellow) and RADICL-seq (blue) datasets. Data are presented as mean ± s.d.; statistical significance was calculated with one-sided two-proportions *z* test; *P ≤ 0.05. **e** Comparison of Malat1 transcript target DNA loci in mESCs identified by RAP-DNA (yellow), RADICL-seq (grey), and GRID-seq (blue) methods. **f** Comparison of Rn7sk transcript target DNA loci in mESCs identified by ChIRP-seq (yellow), RADICL-seq (grey), and GRID-seq (blue) methods. All panels were generated using RADICL-seq total dataset. Source data are available in the Source Data File.

RADICL-seq differs from GRID-seq in four main technical aspects (Fig. 2a), as described below:

(i) In the fixation step, GRID-seq employs a higher concentration of FA and uses disuccinimidyl glutarate, a strong protein–protein crosslinker, thus capturing RNA and DNA linked together indirectly via multiple protein intermediates^15^.

(ii) The GRID-seq protocol employs the type II restriction enzyme *Mme*I to trim RNA- and DNA-interacting sequences, generating 20-nt tags as opposed to the 27-nt tags produced by the *Eco*P15I restriction enzyme, thus resulting in tags that are more difficult to map uniquely to the genome. Indeed, when we compared the percentage of sequencing reads that can be mapped to the mouse genome, RADICL-seq outperformed GRID-seq with a more than a 3-fold increase in uniquely mappable reads (45% vs. 14%) (Fig. 2b). To confirm this result, we artificially trimmed down RADICL-seq tags (i.e., 27 nt) to generate RNA and DNA reads with lengths similar to those obtained by GRID-seq tags (i.e., 20 nt) and observed a dramatic reduction in the fraction of uniquely mapped RNA–DNA interactions (from 45 to 10%), comparable to the rate observed for the GRID-seq dataset and in concordance with previous findings^16^. This difference affects both the number and the type of detected interactions because reads encompassing repetitive regions are intrinsically more difficult to map than those from other genomic regions.

(iii) GRID-seq technology digests genomic DNA with *Alu*I, whereas the RADICL-seq protocol employs a controlled DNase I digestion that avoids the sequence biases encountered with restriction enzymes, and, therefore, generates a more homogeneous shearing of the chromatin. When we looked at the genomic coverage of DNA regions identified by both technologies, RADICL-seq exhibited higher coverage (Fig. 2c). We additionally observed that RADICL-seq genome coverage increased proportionally with the sequencing depth, whereas the coverage of GRID-seq converged to a plateau (Fig. 2c).

(iv) Finally, RADICL-seq employs RNase H treatment prior to the RNA ligation step to reduce the number of captured interactions generated by nascent transcription and consequently increases the variety of captured RNA–DNA interactions. When compared with uniquely mapped RNA and DNA reads detected by GRID-seq, the RADICL-seq dataset showed increased detection of intergenic transcripts and ncRNAs (Supplementary Fig. 5a). Moreover, in GRID-seq data we observed both a higher contribution of intronic coding RNA reads (66.9% vs. 54.7%) (Supplementary Fig. 5a) and a 2.5-fold increase in RNA–DNA interactions occurring at a distance below 1 kb than in the RADICL-seq data (Fig. 2d), which suggests a higher content of nascent transcripts in this set of interactions. Furthermore, GRID-seq DNA reads exhibited stronger enrichment for the H3K36me3 signal, a marker of elongating RNA pol II (Supplementary Fig. 5b), suggesting a stronger bias for nascent transcription when compared with RADICL-seq. In contrast, RADICL-seq captured RNA sequences derived from the bodies of annotated genes and enriched for histone modifications associated with exonic regions (H3K4me3 and H3K9Ac; first exon and intron regions were removed for the analysis, Supplementary Fig. 5c, d), indicating enrichment for mature transcripts^17^. Notably, RADICL-seq uses a similar amount of input cells as GRID-seq, but it achieves higher detection power by yielding a higher number of informative RNA–DNA interactions at a lower cost. The publicly available GRID-seq dataset has higher sequencing depth (156.6M raw/total reads), but yields less usable reads (14M uniquely mapped reads; 8.9% of the total)^9^, whereas RADICL-seq was sequenced at a lower depth (121.9M total reads) and produced 19.9M uniquely mapped reads (16.3% of all sequenced reads). Moreover, when comparing the capture rate for different classes of transcripts between the two technologies, we observed that RADICL-seq detects a higher number of RNAs from different biotype groups compared to GRID-seq (up to one order of magnitude for antisense, lincRNAs and protein-coding classes, Supplementary Table 1), thus widening the spectrum of RNA–DNA interactions that is otherwise unattainable with GRID-seq.

Interestingly, while GRID-seq displayed a larger fraction of captured *cis* (i.e., intrachromosomal) interactions, RADICL-seq recovered ~30% more *trans* interactions (i.e., interactions where RNA and DNA tags in the pair are mapped to different chromosomes) (Fig. 2d), thus providing a vastly expanded interaction dataset for investigating long-range RNA–chromatin associations. We overall observed lower correlation between technologies than within replicates (Supplementary Fig. 5e), suggesting that RADICL-seq and GRID-seq capture different sets of interactions.

We evaluated the extent of known interactions captured by RADICL-seq and GRID-seq by comparing the genomic targets of Malat1 (lncRNA) and Rn7sk (small nuclear RNA) detected by RADICL-seq with those observed using RNA antisense purification, followed by DNA-sequencing (RAP-DNA)^15^ for Malat1 and chromatin isolation by RNA purification (ChIRP-seq)^18^ for Rn7sk. Malat1 targets detected by RADICL-seq data had a genomic distribution comparable to that of Malat1-targeted RAP-DNA libraries prepared from pSM33 ES cells^15^ (Supplementary Fig. 5f). The RADICL-seq list of targets confirmed 78% of Malat1-decorated protein-coding genes in the RAP-DNA library compared with 69% of targets found by GRID-seq (Fig. 2e). When compared with GRID-seq, RADICL-seq exhibited a lower percentage of genomic targets that were not detected by RAP-DNA. For the genomic targets of Rn7sk detected by ChIRP-seq^18^, RADICL-seq detected over 20-fold more interactions mediated by Rn7sk than did GRID-seq (9.491 and 0.357 average normalized counts in RADICL-seq and GRID-seq, respectively). Furthermore, RADICL-seq detected 56% of the protein-coding genes interacting with Rn7sk, compared with only 13% of targets for GRID-seq (Fig. 2f).

Collectively, these analyses suggest that RADICL-seq captures RNA–chromatin interactions more comprehensively and with less genomic bias when compared with GRID-seq.

Recently, a technology that maps DNA–DNA contacts, split-pool recognition of interactions by tags extension (SPRITE)^19^ has been published. SPRITE has also the capability to map RNA–chromatin interactions, and it has been used to map RNA–DNA contacts in mESCs. However, we noticed that out of 52.5M total DNA–DNA and RNA–DNA complexes identified by SPRITE, only 0.275M (0.5%) contained RNA species, and 80% of these contained rRNA. Hence, this indicates that RNA–DNA interactions detected by SPRITE are only corollary to the overwhelming majority of rRNA-mediated chromatin interactions. Nevertheless, we included SPRITE data in our analysis and compared RNA–DNA interactions captured by existing technologies in mESCs. Compared to other technologies, RADICL-seq exhibited significantly higher resolution for the genomic DNA signal for Malat1 (Supplementary Fig. 5g) and Rn7sk (Supplementary Fig. 5h), respectively.

### Identification of robust RNA–chromatin interactions

The RADICL-seq technology yields a large amount of interaction data with a complexity comparable to that obtained with Hi-C technology. Consequently, to account for the occurrence of spurious events, we decided to adopt an approach similar to that employed in Hi-C analyses^20–22^; this approach assumes that all biases (e.g., amplification biases due to differences in sequence composition across the genome) are reflected in the observed interaction counts. To this end, we partitioned the linear genome into intervals (i.e., bins of 25 kb, see Methods) to represent the RADICL-seq data as a contact matrix between RNA and DNA loci. We then used a one-sided cumulative binomial test to detect significant RNA–chromatin interactions, assuming that the transcript-specific background interaction frequency of a given RNA and a genomic interval depends also on their relative genome-wide coverage^23^. We employed the Benjamini–Hochberg multiple-testing correction to control for the false discovery rate and used an adjusted *P* cut-off of 0.05 to define the “significant” set (Supplementary Fig. 6a). The resulting significant dataset displayed different distributions for specific transcript biotypes (Supplementary Fig. 6b). By applying this method to RADICL-seq data produced from the total dataset, 288,065 unique, robust RNA–DNA-interacting loci, supported by 8,420,123 interactions, were identified as statistically significant (Supplementary Fig. 6c). As expected, many of the *trans* interactions were removed because of their lower occurrence and inherent difficulty in being consistently detected at the chosen sequencing depth (Supplementary Fig. 6c). The RNA–chromatin interactions were mediated by 14,001 transcripts, with a prevalent contribution from protein-coding transcripts (12,441 transcripts; 89%) followed by lncRNAs (1430 transcripts; 10%) (Supplementary Fig. 6d). Furthermore, the RNAs that interacted the most with the chromatin were similar across experimental conditions (Supplementary Table 2).

To compare RNA–chromatin interaction patterns across different cell types, we performed RADICL-seq (using 1% FA) on oli-neu, a neural cell line derived from mOPCs^24^ (Supplementary Fig. 7a). Again, the three biological replicates exhibited high reproducibility, but markedly lower correlation with the mESC biological replicates, suggesting cell specificity in a substantial fraction of the captured interactions (Supplementary Fig. 7b). Interestingly, the proportion of the noncoding transcriptome captured by RADICL-seq in mOPC total dataset showed markedly higher detection of lncRNAs (Supplementary Fig. 7c, d) compared with the equivalent dataset in mESCs (Fig. 1c, d). Although mapping of RNA and DNA tags from the mOPC NPM dataset (Supplementary Fig. 8a–c) revealed a distribution of biotypes similar to the mOPC total dataset, we observed higher variability in the frequency of RNA–DNA interactions between conditions than within replicates (Supplementary Fig. 8d). As for mESCs, we filtered for robust RNA–chromatin interactions in both mOPC datasets (Supplementary Fig. 9a–c), and, hereafter, we have used the significant interactions in all the analyses described below.

**Fig. 3:**
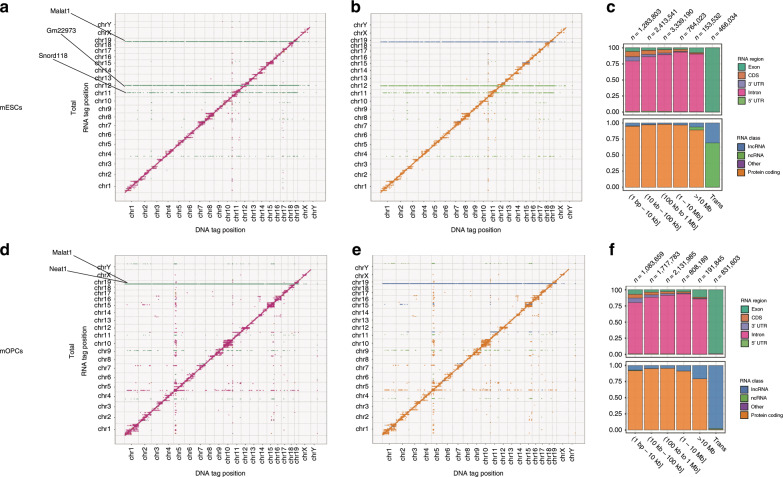
RADICL-seq identifies genome-wide RNA–chromatin interactions. **a**, **b** RNA–DNA interactions in mESCs shown as a single point per 25-kb bin and colored (see key in **c**) by the most-represented RNA region or RNA class, respectively, in that bin. **c** RNA–DNA interactions in mESCs, quantified according to genomic distance between RNA and DNA tags. **d**, **e** RNA–DNA interaction matrix for mOPCs, similar to that shown in **a**, **b**. **f** RNA–DNA interactions in mOPCs, quantified according to genomic distance between RNA and DNA tags. All panels were generated using significant total datasets.

To globally visualize the RNA–DNA interactions in the mESC and mOPC total datasets, we arranged each transcript and its interacting genomic regions in a two-dimensional contact matrix. For each 25-kb bin, the highest contributing RNA region and class were depicted and quantified according to distance categories (Fig. 3a–c). A clear trend for proximal interactions emerged, highlighted by a diagonal signal dominated by intronic RNA signal (Fig. 3a) derived from protein-coding genes (Fig. 3b). On the one hand, we observed that the number of interactions in *cis* from intronic regions increased with the distance to the genomic region bound by the transcript, and on the other hand, we observed a dominant contribution of exonic regions from noncoding transcripts in the *trans* interactions (Fig. 3c). Remarkably, a few noncoding transcripts, such as Malat1, the small nuclear RNA Gm22973, and the small nucleolar RNA Snord118, which is involved in splicing, exhibited extensive *trans-*interaction patterns (Fig. 3a). In mOPCs, we observed the same trend of dominant contribution of intronic protein-coding and exonic noncoding transcripts for *cis* and *trans* interactions, respectively (Fig. 3d–f). In addition, most lncRNAs displayed preferential binding to chromatin with local (≤10 kb), short- (>10 and ≤100 kb) and medium-range (>100 kb and ≤1 Mb) *cis* patterns in both cell types (Supplementary Table 3).

To further strengthen the substantial difference between RADICL-seq and GRID-seq technologies, we proceeded by analyzing their datasets of significant interactions. We confirmed that RADICL-seq retains a much higher genomic coverage than GRID-seq even when we consider only significant interactions (Supplementary Fig. 10a). In order to investigate whether GRID-seq data would be able to find similar results to those obtained with RADICL-seq (Fig. 3), we called significant interactions from GRID-seq data by using the same statistical approach we employed for RADICL-seq. Then, we plotted the proportion of specific RNA biotypes for different intervals of RNA–DNA interaction distances (Supplementary Fig. 10b). The comparison between the two technologies shows that GRID-seq captures considerably less long-range *cis* and *trans* RNA–chromatin interactions, specifically those mediated by ncRNAs (Supplementary Fig. 10c).

Global patterns of interactions indicated clear differences between the two cell types for both *cis* and *trans* interactions. Specifically, we observed large domains of *cis* interactions distributed along each chromosome with various transcripts interacting with broad regions of the chromosome from which they originate in a cell-type-specific manner (Supplementary Fig. 11a, b). For example, Pvt1 contacted large portions of its chromosome of origin in both cell types, whereas Malat1 interacted widely across the genome with cell-type-specific patterns, and Gm22973 contacted multiple chromosomes but only in mESCs (Supplementary Fig. 11a, b).

We sought to investigate whether the DNA binding could be connected to the transcriptional levels of the RNAs involved in the interactions. Accordingly, we generated cap analysis of gene expression (CAGE) data from the nuclear fraction of mESCs and mOPCs, and divided the genic transcripts into expression quartiles (I: lowest; IV: highest). When we plotted the number of interactions as a function of the distance to the gene body in the total datasets of both cell types, we observed a sharper decay rate for transcripts that were expressed at lower levels, suggesting that the expression plays a role in the establishment of RNA–DNA contacts proximal to the transcription locus (Supplementary Fig. 12a, b). However, we noticed that a fraction of *cis* interactions occurring at considerable distance from the gene body were still present after treatment with ActD, which suggests that factors other than transcriptional activity might play a role in the formation of such contacts (Supplementary Fig. 12a). Indeed, in ActD-treated mESCs, we still observed a prevalent intronic RNA signal from protein-coding genes in *cis* interactions (Supplementary Fig. 13a–c); however, the inhibition of RNA pol II elongation resulted in a strong depletion of the signal in long- (>1 Mb and ≤10 Mb, 1.4% vs. 9.1% in total dataset) and extreme long-range (>10 Mb, 0.5% vs. 1.8% in total dataset) *cis* interactions (Supplementary Fig. 13c). Furthermore, the contribution of ncRNAs was increased in the subset of extreme long-range *cis* interactions, and, interestingly, a significant number of *trans* interactions were preserved (Supplementary Fig. 13a, b) and appeared in the same regions of the genome as in the total dataset (Fig. 3a, b).

In the NPM datasets of both cell types, the broadening of the signal from the diagonal observed in the total datasets (Fig. 3a, b) was completely lost (Supplementary Fig. 13d, e, g, h), suggesting a relevant contribution of proteins in the establishment of RNA–chromatin interactions. Genome-wide binding of specific noncoding transcripts was absent, although we observed genomic regions highly bound by RNAs with *cis* and *trans* contacts. Moreover, we observed a dramatic drop in *trans* interactions involving exonic RNAs from noncoding genes, which suggests that most of these interactions are protein mediated (Supplementary Fig. 13f, i).

To further investigate the nature of direct binding between RNA and DNA molecules, we examined the distribution of the distances between interacting RNA and DNA tags in the mESC and mOPC NPM datasets (Supplementary Fig. 14a). We observed a dominant contribution (>85%) of interactions where both RNA and DNA tags were complementary to each other (distance of <1 kb) compared with only 4.5% in the total datasets (Supplementary Fig. 14a, b). Next, we looked at the overlap of DNA tags captured by the NPM and total datasets with available DRIP-seq data that maps the location of R-loops genome-wide in mESCs^25^ (Supplementary Fig. 14c). There was a relative enrichment of RADICL-seq DNA tags across DRIP-seq peaks in the NPM dataset compared with the total dataset, possibly indicating an over-representation of R-loops. As the remaining fraction of direct RNA–DNA interactions in the NPM dataset could not be explained by complementarity of RNA and DNA strands, we examined whether these interactions could be mediated by the formation of triple-helical nucleic acid structures^26^. To this end, we analyzed the interactions of lncRNAs that are known to form triple helices with DNA^26,27^, namely Malat1 and Meg3. We found that these transcripts were among the lncRNAs with the highest number of *trans* contacts (Supplementary Table 4) and that they are likely to form triplexes in close proximity to these contacts (Supplementary Fig. 14d–i).

### Influence of 3D chromatin architecture on RNA–DNA interactions

To better understand the relationship between the interactions captured by RADICL-seq and the 3D architecture of the genome (i.e., DNA–DNA contacts and/or genomic distance), we leveraged Hi-C data produced from similar cell types (mESCs and neural progenitor cells)^28^. At 25-kb resolution, the frequencies of *cis* RNA–DNA contacts in the total datasets moderately correlated with normalized Hi-C DNA–DNA contacts (Pearson’s correlation coefficient = 0.56 for both cell types, Supplementary Fig. 15). However, about 30% of the variance (based on the above correlation coefficient) in the *cis* RNA–DNA contact frequency could be explained by genomic architecture. The remaining ~70% of variance in RNA–DNA contacts is most likely a combination of noise and true signal that is not linearly dependent on spatial distance between genomic loci.

**Fig. 4:**
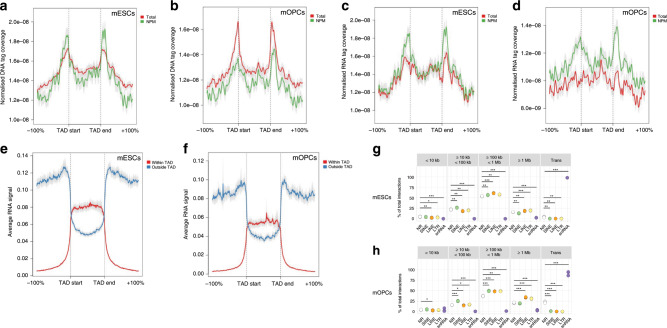
RNA interactions and genomic structural features. **a**–**d** Metadata profiles showing the average coverage of **a**, **b** DNA tags or **c**, **d** RNA tags at the boundaries of TADs in mESCs (**a**, **c**) and mOPCs (**b**, **d**) for the indicated conditions. **e**, **f** Average RNA-binding signal at TAD boundaries in total mESCs and mOPCs, respectively. The signal was split based on whether the RNA originated within or outside of the TAD. Coverage distance from the TAD is relative to the corresponding TAD width. **g**, **h** Percentage of RNA–DNA interactions in total RADICL-seq libraries from mESCs and mOPCs, respectively, divided into incremental RNA–DNA distances and with RNA tags grouped by the identity of intersected repeat elements. NR, RNA tags not mapped on any repeat. Statistical significance was calculated with the two-tailed Student’s *t* test. *P ≤ 0.05; **P ≤ 0.01; ***P ≤ 0.001. All panels were generated using significant datasets. Source data are available in the Source Data File.

We investigated the distribution of DNA tags with respect to topologically associating domains (TADs) identified in the above Hi-C data^28^ and found a clear signal enrichment at TAD boundaries in both cell types total and NPM datasets (Fig. 4a, b). This finding was replicated also in the mESC ActD dataset. (Supplementary Fig. 16a). Furthermore, we observed enrichment for RNA tags at TAD boundaries primarily in the NPM condition in both cell types (Fig. 4c, d and Supplementary Fig. 16b). Interestingly, we obtained a similar enrichment for DRIP-seq signal^25^ at the TAD boundaries, possibly suggesting a relationship between TAD formation and the generation of R-loops (Supplementary Fig. 16c). These results underline the importance of including the NPM condition to capture biological features that might be otherwise overlooked by solely using total datasets. Finally, we surveyed RNA–DNA interactions located in A and B compartments (i.e., preferentially open and closed chromatin, respectively) for both the mESC and mOPC total datasets (Supplementary Fig. 16d). Although we did not observe significant enrichment of RNA classes (protein coding or lncRNA) for any compartment, the results showed a clear segregation of A and B compartments with transcripts originating from one compartment mainly involved in interactions within the same compartment.

Next, we asked whether RNAs originating from loci positioned within or outside TADs showed specific DNA-binding patterns, possibly dictated by genomic structural constraints. When we looked at the distribution of the signal for RNAs transcribed within TADs in the mESC and mOPC total datasets, we found it to dramatically drop outside the domain regions, whereas the signal from transcripts transcribed outside TADs showed the opposite trend (Fig. 4e, f), thus suggesting a barrier effect for the RNA migration into or out of TADs that prevents free diffusion. Collectively, our results highlight a putative role for TADs in shaping RNA–chromatin interactions in mESCs, mOPCs, and possibly other cell types.

### Transcripts containing repeat elements differentially engage in specific chromatin interactions

Repeat elements (REs) have emerged in recent years as key contributors to genomic regulation and organization^29^. In the mESC and mOPC total datasets we observed that ~12% and ~8%, respectively, of the uniquely mapped genic RNA tags intersected with REs as defined by RepeatMasker^30^ (Supplementary Fig. 17). In mESCs, the most abundant classes of intragenic REs were small nuclear RNA (snRNA) (~39%) and SINE (short interspersed elements) (~35%), followed by LINE (long interspersed elements) and LTR (long terminal repeat) (both ~9%) (Supplementary Fig. 18). In mOPCs the most abundant classes were SINE (~48%) followed by LINE (~20%) and LTR (~15%); intriguingly, the frequency of snRNAs involved in RNA–DNA interactions was dramatically lower in mOPCs (<1%) than in mESCs (Supplementary Fig. 18). We annotated non-self RNA–chromatin interactions for the most abundant classes of intragenic REs across increasing distances from the site of transcription. When we compared them to interactions not involving REs, we found a remarkably well-defined RE-specific pattern of *cis* interactions that was reproducible in both cell types (Fig. 4g, h). In terms of differences among different RE families, RNA–DNA pairs where the RNA mapped to SINE were found to be enriched at distance intervals of ≥10 kb and <1 Mb, whereas RNAs that mapped to LINE and LTR were proportionally depleted at linear distances of <100 kb, but significantly enriched at longer range intervals (Fig. 4g, h) even in the absence of nascent transcription (Supplementary Fig. 19). Although pairs where the RNA mapped to SINE, LINE, or LTR displayed no *trans* interactions, those mapping to snRNAs exhibited extensive *trans* interactions (>95%, Fig. 4g, h and Supplementary Fig. 19). Collectively, these analyses show that transcripts containing REs are engaged in *cis* interactions with the chromatin, which is in agreement with previous studies that reported their association with euchromatin^29^.

### RADICL-seq identifies cell-type-specific RNA–DNA interactions

**Fig. 5:**
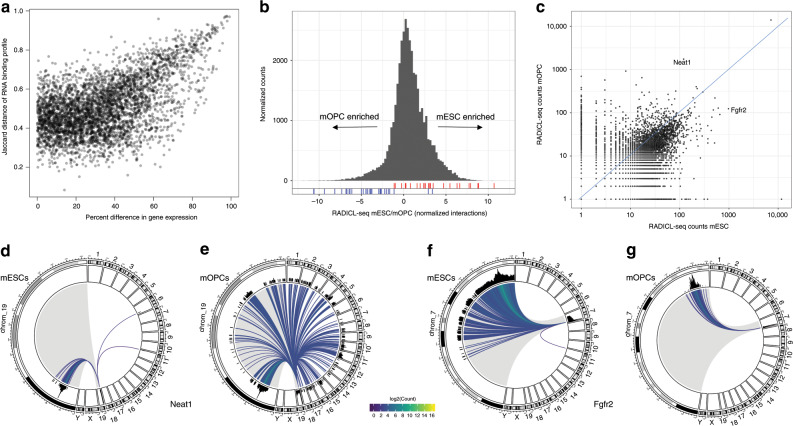
Cell-type-specific RNA–chromatin interaction patterns. **a** For each gene, the Jaccard distance between the genome-wide RNA–DNA binding profiles in mESCs and mOPCs is compared with the difference in capture rate (proxy for gene expression) between the two cell types. **b** Distribution of the log_2_ ratios of RADICL-seq mESC to mOPC DNA normalized tag counts for targeted gene promoter regions, which are defined as ±2 kb around the TSS. Cell-type-specific marker gene positions are highlighted for both mESCs (red) and mOPCs (blue). Counts were normalized by library size. **c** RADICL-seq counts of unique genomic targets per interacting RNA in mESCs vs. mOPCs. **d**, **e** Circos plots depicting Neat1 genomic interactions in mESCs and mOPCs, respectively. **f**, **g** Circos plots depicting Fgfr2 genomic interactions in mESCs and mOPCs, respectively. Each line represents the interaction between the genic transcript and the contacted genomic bin, while its color indicates log_2_ of the RADICL-seq count. The chromosome of origin of the RNA under investigation is shown enlarged (gray shading) on the left portion of each circos plot. All panels were generated using significant total datasets.

To better understand the involvement of RNA in genome organization and the fine-tuning of cell-specific gene expression, we compared genome-wide RNA–DNA binding profiles and capture rates between mESCs and mOPCs. For each transcript, we calculated the Jaccard distance between the RNA–DNA binding profiles as a function of the difference in RADICL-seq capture rate, here considered a proxy for gene expression (Fig. 5a). As expected, there was a clear relationship between differential transcript abundance and differences in RNA–DNA binding profiles. However, even at comparable expression levels, we observed a diversity of interaction patterns among various transcripts between the two cell types (e.g., Hunk and Ralgapa2, Supplementary Fig. 20a–d). When transcripts were divided into differential expression deciles, for genes with <40% difference in gene expression between the two cell types, we found no link between differential gene expression and changes in genome-wide binding profile (Supplementary Fig. 20e). These results suggest that, although expression plays a role in determining the binding pattern of a transcript, the DNA binding profile for genes expressed at similar levels may be governed by other factors, such as 3D chromatin architecture or epigenomic state.

Next, we assessed whether RADICL-seq can be used to discriminate biologically relevant differences between cell types. To this end, we collected from the literature a list of marker genes that are specifically expressed in mESCs or mOPCs (Supplementary Table 5) and used RADICL-seq to analyze patterns of RNA–chromatin regulation at the genomic regions containing these markers. To compare cell-specific RNA–chromatin interactions at the gene level, we used the total datasets to calculate the mESC/mOPC ratio of the normalized RADICL-seq counts of bound RNAs for the promoter regions (defined as ±2 kb around the transcription start site [TSS]) of all genes. We examined the positions of the cell-specific markers in the distribution of these mESC/mOPC ratios and found that they segregated towards the tails of the distribution according to the cell line in which they were expected to be more highly expressed (Fig. 5b). Our results suggest that the technology is able to discriminate cell-type-specific features.

To investigate in greater detail whether RNA–chromatin interactions could play a role in gene expression, we employed CAGE data to annotate de novo promoters in mESCs and mOPCs. For both total datasets, we compared the distribution of unique RNAs interacting with promoter regions of genes that are transcriptionally active with those that are inactive. We did not observe any clear difference between the two groups in either cell type (Supplementary Fig. 21a, b), indicating that gene expression does not seem to be dependent on the number of unique interacting RNAs. However, the top 100 promoters of the genes that interacted with the highest number of unique RNAs clustered in different chromosomes in the two cell types: predominantly in chromosomes 8 and 11 in mESCs and in chromosome 5 in mOPCs (Supplementary Fig. 21c, d).

When comparing the number of unique genomic targets for each transcript detected in the total datasets for mESCs vs. mOPCs, we observed a linear correlation with the presence of some outliers (Fig. 5c). Among the RNAs that showed large deviations from the diagonal (i.e., transcripts that had dissimilar patterns of interactions between the cell types), we selected Neat1 and Fgfr2 as representative examples of transcripts having higher RADICL-seq counts in mOPCs and mESCs, respectively. The nuclear lncRNA Neat1 is one of the main components of paraspeckles, membrane-less compartments present in the nucleus of differentiated cells^31^. In mESCs, Neat1 exists as a shorter isoform, which is unable to promote the formation of paraspeckles, whereas its longer isoform is expressed in differentiated cells^32,33^. Consistent with these observations, RADICL-seq RNA reads mapped only to the shorter isoform in mESCs, whereas the signal covered the whole span of the longer isoform in mOPCs (Supplementary Fig. 22a). Furthermore, the genomic binding pattern of Neat1 exhibited dramatic differences between the two cell types, with the lncRNA interacting mostly in *cis* in mESCs as opposed to the extensive *trans* interactions mediated in mOPCs (Fig. 5d, e). Analysis of the DNA-binding regions of Neat1 confirmed the preference for binding to the 5ʹ ends of the target genes observed previously^34^ (Supplementary Fig. 22b, c). Fgfr2 is a protein-coding gene with an important role in pluripotency; mutations affecting its expression result in early embryonic lethality due to inner cell mass defects^35^. Unlike the localized pattern exhibited in mOPCs, Fgfr2 displayed extensive *cis* interactions in mESCs, covering above 30% of the chromosome from which it is transcribed (Fig. 5f, g and Supplementary Fig. 11a). The RADICL-seq results thus suggest a potentially structural role for Fgfr2 in mESCs.

Finally, we turned our attention to *trans* RNA–DNA interactions. To compare the extent of overlap between the two cell types, we calculated the intersection of RNA–chromatin interactions categorized by linear distance in the total datasets (Supplementary Fig. 23). To our surprise, we found 3414 unique (12.2%) *trans* RNA–DNA pairs shared by both cell types; this percentage of overlap was similar to that for *cis* RNA–chromatin interactions separated by a linear distance of ≥1 Mb and <10 Mb (Supplementary Fig. 23). Furthermore, these *trans* interactions captured in both cell types were mediated by 14 transcripts with a major contribution from Malat1 (Supplementary Table 6). These results highlight the contribution of Malat1 in the organization of general principles of RNA–chromatin interactions.

## Discussion

RADICL-seq provides four main advantages over existing methods: (i) Chromatin shearing is achieved by a controlled DNase I digestion, which results in a greater resolution compared with digestion with restriction enzymes. Indeed, the distribution of cut sites for restriction enzymes is often uneven and may result in the inability to detect important interaction chromatin regions. The sequence-independent digestion of chromatin by DNase I enables RADICL-seq to overcome such resolution limitations. (ii) The use of paramagnetic carboxylated beads as carriers for the nuclei allows additional washes to remove small genomic fragments (upon DNase digestion) and the excess of biotinylated bridge adapter, thus reducing noise. Furthermore, beads improve visualization of the nuclear pellet when using fewer cells and can potentially decrease the number of input cells for future applications. (iii) Digestion of RNA–DNA hybrids with RNase H reduces the fraction of nascent RNA–chromatin interactions captured by RADICL-seq, thereby increasing the capture rate of other types of interaction. (iv) Use of *Eco*P15I to generate RNA and DNA reads of uniform size greatly improves unique alignment to the genome. Furthermore, RADICL-seq uses the same amount of input cells as GRID-seq, but it achieves a higher detection power for uniquely mapped RNA–DNA interactions and consequently a better performance/cost ratio.

Our results confirm previous observations regarding modality of interaction for lncRNAs, with most noncoding transcripts binding genomic targets locally or over short- and medium-range distances in *cis*^36^. However, one surprising finding was the dominant contribution of intronic RNA sequences from protein-coding genes in *cis* interactions. These binding events seem to be of stable nature, as we observe interactions mediated by intronic RNAs following inhibition of transcription elongation for several hours. Excised introns have been reported to exert a biological function on growth phenotype in yeast^37^. We speculate the existence of a similar mechanism in higher eukaryotes where specific intronic RNA sequences might escape degradation and interact with the chromatin in *cis*. Additionally, a subset of interactions mediated by intronic RNAs from protein-coding genes might be involved in the transcriptional regulation of neighboring genes mediated by protein-coding transcripts as previously reported^38^.

To test our hypothesis of the involvement of genome structure in transcriptional regulation mediated by RNA interactions, we used RADICL-seq to assess the frequency of RNA–DNA interactions at TAD boundaries. The widespread enrichment of RNA–chromatin interactions at these boundaries indicates a possible role for transcription or for its products in influencing 3D genome structure. CHAR-seq technology identified enrichment of transcription-associated RNAs at TAD boundaries in *Drosophila melanogaster*, suggesting an evolutionary conservation of this phenomenon. Intriguingly, Heinz et al.^39^ have reported that transcription elongation remodels 3D chromatin architecture by displacing TAD boundaries. Moreover, SINE sequences have previously been found to be enriched at TAD boundaries^40^. With RADICL-seq we have uncovered specific patterns of interactions for transcripts overlapping REs, thus suggesting that transcripts from REs might facilitate the formation of 3D structures in cells, especially after cell division when TADs are temporarily dismantled.

The observed enrichment of CHARs at TAD boundaries even in the ActD dataset—where RNA pol II elongation is inhibited—potentially suggests that TADs might generate a barrier effect, which is consistent with the transcripts preferentially binding to DNA within or outside, but not across, TADs. This observation confirms recent evidence of a TAD-restricted genome occupancy mediated by immune gene-priming lncRNAs^41^.

The NPM datasets identified reproducible RNA–chromatin interactions that are not mediated by the presence of proteins. Such interactions can be explained either by direct RNA–DNA pairing or by binding of RNA to duplex DNA via the formation of triplexes. Increased frequency of RNA–chromatin interactions and enrichment for R-loops at TAD boundaries in NPM datasets indicate the possible role of active transcription in partitioning the genome, as disruption of the cohesin/CTCF complex in mammals does not lead to disappearance of TAD boundaries^42^. Although the RADICL-seq protocol includes an RNase H step to remove RNAs paired with DNA in Watson–Crick fashion while the sample is still crosslinked, proteins located nearby the complementary RNA–DNA binding could hinder enzyme accessibility, thus preventing the complete digestion of these hybrids. Nevertheless, we were still able to observe a consistent number of interactions that can be explained by triple helix formation. Future extensions of the protocol could include an RNase H step after reversal of crosslinks to enrich for triplexes structures. Although a protein-free method that separately identifies RNA and DNA involved in triple helix formation has been published^43^, to our knowledge RADICL-seq performed in the NPM condition is the only approach that can simultaneously link interacting RNA and DNA to triplex structures.

Our results further show that RADICL-seq can pinpoint cell-type-specific RNA–chromatin interactions. We uncovered an mOPC-specific genome occupancy pattern for Neat1 RNA where the long isoform interacts in *trans* with the chromatin. Recently, Katsel et al.^44^ have reported down-regulation of Neat1 expression in schizophrenia patients, which is associated with reduction in the number of cells of the oligodendrocyte lineage.

In summary, we have developed a technology to map genome-wide RNA–chromatin interactions that significantly improves upon other existing technologies by reducing nascent transcription bias and increasing genomic coverage and unique mapping rate efficiency. Application of RADICL-seq in mESC- and mOPC-derived cells allowed the unveiling of principles of RNA–chromatin *cis* and *trans* interactions, and the identification of the cell type specificity of such associations. We anticipate that the RADICL-seq technology will pave the way for a deeper understanding of the fine regulatory network governing gene expression and ultimately cell identity.

## Methods

### Cell Culture

R08 mESCs (CIRA) were grown under feeder-free conditions in mouse ESC medium consisting of Dulbecco’s modified Eagle’s medium (DMEM; Wako) supplemented with 1000 U/ml leukemia inhibitory factor (Millipore), 15% fetal bovine serum (Gibco), 2.4 mM l-glutamine (Invitrogen), 0.1 mM non-essential amino acids (Invitrogen), 0.1 mM 2-mercaptoethanol (Gibco), 50 U/ml penicillin, and 50 μg/ml streptomycin (Gibco). Culture media were changed daily, and cells were passaged every 2–3 days. For the ActD-treated RADICL-seq libraries, R08 mESCs were treated with ActD (Sigma) at a final concentration of 5 µg/ml for 4 h before crosslinking, as described below.

Oli-neu cells were grown on poly-l-lysine-coated dishes and expanded in proliferation media consisting of DMEM (Lifetech), N2 supplement, penicillin–streptomycin–glutamine (Lifetech), T3 (3,3′,5′-triiodo-l-thyronine; Sigma-Aldrich) 340 ng/ml, l-thyroxine (Sigma-Aldrich) 400 ng/ml, fibroblast growth factor-basic 10 ng/ml, and platelet-derived growth factor-BB 1 ng/ml. Culture media were changed on alternate days and the cells were passaged every 5–6 days.

The origins, authentications, and mycoplasma-testing methods of the cell lines used in the current study are listed in the Reporting Summary.

### Crosslinking of cells

Confluent cells were rinsed with pre-warmed phosphate-buffered saline (PBS) and trypsinized. Detached cells were pelleted, resuspended in PBS, counted, and pelleted again. Cell pellets were then crosslinked by resuspension in freshly prepared 1% or 2% FA (Thermo Fisher Scientific) solution using 1 ml for every one million cells. Cells were incubated at room temperature for 10 min with rotation, followed by quenching with 125 mM glycine (Sigma). Cells were pelleted at 4 °C, washed with ice-cold PBS, pelleted again, and snap frozen in liquid nitrogen.

We use the term biological replicates to refer to batches of cells with different passage numbers.

### Generation of RADICL-seq libraries

Adenylation of the adapter. The adapter is a partially double-stranded DNA molecule containing chemical modifications (5ʹ phosphorylation [5Phos] and Internal Biotin dT [ibiodT]; Integrated DNA Technologies). The upper strand sequence is 5ʹ-/5Phos/CTGCTGCTCCTTCCCTTTCCCCTTTTGGTCCGACGGTCCAAGTCAGCAGT-3ʹ. The lower strand sequence is 5ʹ-/5Phos/CTGCTGACT/ibiodT/GGACCGTCGGACC-3ʹ. The upper strand was pre-adenylated by using a DNA 5′ Adenylation Kit (New England BioLabs). The pre-adenylated upper strand was mixed with an equimolar quantity of the lower strand and subsequently incubated at 95 °C for 2 min, followed by 71 cycles of 20 s, with a reduction of 1 °C every cycle. Annealed pre-adenylated adapter was then purified using a Nucleotide Removal Kit (Qiagen).

Chromatin digestion. Chromatin preparation was performed using a modified protocol^45^. Briefly, cell pellets containing approximately two million crosslinked cells were resuspended in cold lysis buffer (10 mM Tris-HCl, pH 8.0, 10 mM NaCl, 0.2% NP-40) and incubated on ice for 10 min. Nuclei were pelleted at 2500 × *g* for 60 s, resuspended in 100 μl of 0.5× DNase I digestion buffer (Thermo Fisher Scientific), containing 0.5 mM MnCl_2_ and 0.2% sodium dodecyl sulfate (SDS), and incubated at 37 °C for 30 min. An equal volume of 0.5× DNase I digestion buffer containing 2% Triton X-100 was added, and then incubation at 37 °C was continued for 10 min. Then, 1.5 U DNase I (Thermo Fisher Scientific) was added and digestion was carried out at room temperature for 4 or 6 min for 1 and 2% FA, respectively. DNase I digestion was stopped by adding 40 μl of 6× Stop Solution (125 mM EDTA, 2.5% SDS), followed by centrifugation at 2500 × *g* for 60 s. Nuclei were resuspended in 150 μl nuclease-free H_2_O and purified with two volumes (300 μl) of AMPure XP magnetic beads (Beckman Coulter). After 5 min incubation at room temperature, beads were separated using a magnetic rack, washed twice with 80% ethanol, and air dried for 2 min.

Chromatin end-repair, dA-tailing, and RNase H treatment. The purified bead–nuclei pellet was resuspended in 200 μl 1× T4 DNA ligase buffer (New England Biolabs) containing 0.25 mM dNTPs, 0.075 U/μl T4 DNA polymerase (Thermo Fisher Scientific), and 0.15 U/μl Klenow fragment (Thermo Fisher Scientific), and then incubated at room temperature for 1 h. The end-repair reaction was stopped by adding 5 μl of 10% SDS. The bead–nuclei mixture was pelleted at 2500 × *g* for 60 s; resuspended in 200 μl 1× NEBuffer 2 (New England Biolabs) containing 0.5 mM dATP, 1% Triton X-100, and 0.375 U/μl Klenow (exo-) (Thermo Fisher Scientific), and then incubated at 37 °C for 1 h. After that, 0.122 U/μl RNase H (New England Biolabs) was added, and the reaction was incubated at 37 °C for a further 40 min. The dA-tailing and RNase H reactions were stopped by adding 5 μl of 10% SDS.

Bridge adapter RNA ligation. The bead–nuclei mixture was pelleted at 2500 × *g* for 60 s and then resuspended in 200 μl H_2_O. To remove soluble RNA, 165 μl of 20% polyethylene glycol (PEG) in 2.5 M NaCl was added to the mixture, followed by a 5-min incubation at room temperature. Beads were collected with a magnetic rack, washed once with 80% ethanol, and resuspended in 200 μl H_2_O. This purification step was repeated once. After the second ethanol wash, the air-dried bead–nuclei mixture was resuspended in 23 μl H_2_O, 3 μl 10× T4 RNA ligase buffer, 1 μl pre-adenylated and biotinylated bridge adaptor (20 μM), 1 μl RNaseOut (Thermo Fisher Scientific), and 13.3 U/μl T4 RNA ligase 2, truncated KQ (New England Biolabs). The mixture was incubated at 20 °C overnight to ligate the pre-adenylated bridge adapter to the 3ʹ-OH of the RNA molecules. The reaction was stopped by adding 5 μl 10% SDS, and the bead–nuclei mixture was then pelleted at 2,500 × *g* for 60 s and resuspended in 200 μl H_2_O. To remove excess unligated adapter, 165 μl of 20% PEG in 2.5 M NaCl was added to the mixture, and then the reaction was incubated at room temperature for 5 min. Beads were then collected with a magnetic rack, washed once with 80% ethanol, and then resuspended in 200 μl H_2_O. This purification was repeated once.

Proximity ligation. The in situ proximity ligation was carried out by resuspending the air-dried bead–nuclei mixture in 500 μl 1× T4 DNA ligase buffer with ATP containing 4 U/μl T4 DNA ligase (New England Biolabs) and incubating it at room temperature for 4 h. After the incubation, bead–nuclei complexes were pelleted at 2500 × *g* for 60 s and resuspended in 200 μl H_2_O. To remove remaining unligated and DNA-only ligated adapter, 165 μl of 20% PEG in 2.5 M NaCl was added to the mixture, and then purified as previously described. Beads were then resuspended in 200 μl H_2_O.

Reversal of crosslinking and purification of RNA–DNA complexes. Reversal of crosslinks was performed by adding 50 μl of proteinase K solution (10 mM Tris-HCl, pH 7.5, 1% SDS, 15 mM EDTA) and 1.6 U/μl proteinase K (Ambion) to the resuspended beads. The mixture was incubated overnight at 65 °C and the RNA–DNA complexes were then precipitated with 3 μl GlycoBlue (Ambion), 28 μl 3 M sodium acetate, pH 5.2, and 303 μl isopropanol for 1 h on ice followed by 20,000 × *g* centrifugation at 4 °C for 30 min. The resulting bead–nucleic acids pellet was resuspended in 100 μl H_2_O and further purified using 100 μl AMPure XP beads. After 5 min incubation at room temperature, beads were separated using a magnetic rack, washed twice with 80% ethanol, and then air dried for 2 min. DNA was eluted using 130 μl H_2_O and quantified with a Qubit High Sensitivity Kit (Thermo Fisher Scientific).

Reverse transcription and second-strand synthesis of the RNA–DNA complexes. Since reverse transcriptase can use DNA sequences as primers for the polymerization, the double-stranded region of the bridge adapter acts as the primer for the reaction. The RNA ligated to the bridge adapter was reverse transcribed after the sample was concentrated to a final volume of 12 μl. First, 1 μl of 10 mM dNTPs was added to the sample, and the mixture was incubated at 65 °C for 5 min. Subsequently, 4 μl of 5× first-strand buffer, 1 μl of 0.1 M dithiothreitol, and 1 μl each of RNaseOut and SuperScript IV (both from Thermo Fisher Scientific) were added, and the reaction was incubated at 56 °C for 10 min and 80 °C for 10 min.

Next, the generated complementary DNA (cDNA)–RNA hybrid was converted to double-stranded DNA through a second-strand synthesis reaction by the addition of 30 μl of 5× second-strand buffer (Thermo Fisher Scientific), 3 μl of 10 mM dNTPs (Thermo Fisher Scientific), 3 μl of RNase H (2 U/μl, Thermo Fisher Scientific), 4 μl of *Escherichia coli* DNA polymerase I (New England Biolabs), 1 μl of *E. coli* ligase (New England Biolabs), and H_2_O to the reverse transcription sample, in a final volume of 150 μl. The mixture was incubated at 16 °C for 2 h and the reaction was stopped by adding 10 μl of 0.5 M EDTA. The sample was purified using a Nucleotide Removal Kit (Qiagen) by adding 1.6 ml of the buffer PNI to the sample, with a final elution in 50 μl H_2_O. Sample volume was reduced to 8 μl using a Speedvac concentrator (Tomy).

Hairpin ligation and *Eco*P15I digestion of the cDNA–DNA complexes. The sample was then subjected to hairpin linker (5ʹ-/5Phos/GGCCCTCCAAAAGGAGGGCA-3ʹ; Integrated DNA Technologies) ligation to selectively ligate the bridge adapter that was covalently bound only to RNA and therefore prevent subsequent ligation of sequencing adapters. A total of 100 pmol of hairpin linker was mixed with 10 μl of 2× Quick ligase buffer (New England Biolabs), 8 μl of sample, and 1 μl of Quick ligase (New England Biolabs). The reaction was carried out for 15 min at room temperature and was then purified with DNA Clean & Concentrator-5 Kit (Zymo) according to the manufacturer’s instructions. Elution was performed in 50 μl of H_2_O and final volume was reduced to 30 μl.

The sample concentration was measured by using a Qubit dsDNA High Sensitivity Kit (Invitrogen). *Eco*P15I digestion of the double-stranded cDNA–DNA complexes was performed by using 10 U of enzyme for 1.5 μg of DNA in the presence of 5 μl NEBuffer 3.1 (New England Biolabs), 5 μl 10 × ATP, 0.5 μl 10 mM sinefungin (Calbiochem), and H_2_O in a final reaction volume of 50 μl. The sample was incubated at 37 °C overnight.

End preparation and sequencing linkers ligation. Each *Eco*P15I-digested sample was purified using a Nucleotide Removal Kit (Qiagen) by adding 1.3 ml of PNI buffer. The sample was eluted in 50 μl and the volume was further reduced to 20 μl. To prepare the sample for the sequencing linkers ligation, 6.5 μl of 10× reaction buffer, 3 μl of End Prep Enzyme Mix from NEB Next Ultra End-Repair/dA-Tailing Module (New England Biolabs), and H_2_O in a final volume of 65 µl were added to the concentrated sample, and then the reaction was incubated at 20 °C for 30 min and 65 °C for 30 min.

Next, Y-shaped sequencing linkers were prepared. The upper strand (5ʹ-/5Phos/GATCGGAAGAGCGTCGTGTAGGGAAAGAGTGT-3ʹ) and lower strand (5ʹ-CTCGGCATTCCTGCTGAACCGCTCTTCCGATCT-3ʹ) were annealed in 1× NEBuffer 2 (New England Biolabs) at 95 °C for 2 min, followed by 71 cycles of 20 s, with a reduction of 1 °C every cycle.

Sequencing linkers ligation was performed with NEB Next Ultra Ligation Module and 20 pmol of annealed Y-shaped sequencing linkers at 20 °C for 15 min. After ligation, the sample volume was reduced to 40 μl.

Pull-down of the RNA–DNA ligated complexes and PCR titration. The RNA–DNA ligated complexes were pulled-down with MyOne C1 Streptavidin magnetic beads (Invitrogen). A total of 20 μl of beads were washed twice with 1× western blot (WB) buffer (5 mM Tris-HCl, pH 7.5, 0.5 mM EDTA, 1 M NaCl, 0.02% Tween-20), once with 2× WB buffer, and finally resuspended in 40 μl of 2× WB buffer. An equal volume of sample was added to the beads, and the mixture was incubated at room temperature for 20 min with rotation. Isolated RNA–DNA ligated complexes were extensively washed three times with 1× WB buffer, washed once with elution (EB) buffer (Qiagen), and finally resuspended in 30 μl of EB buffer.

PCR cycle check was performed by using Phusion High Fidelity PCR Kit (Thermo Fisher Scientific) with Universal FW primer (5ʹ-AATGATACGGCGACCACCGAGATCTACACTCTTTCCCTACACGACGCTCTTCCGATCT-3ʹ; Invitrogen), Index RV primer (5ʹ-CAAGCAGAAGACGGCATACGAGATBBBBBBCTCGGCATTCCTGCTGAACCGCTCTTCCGATCT-3ʹ; Invitrogen, where BBBBBB is a 6-nt barcode for multiplexing libraries), and 4 µl of isolated libraries. PCR was carried out at 98  °C for 30 s, followed by 14 cycles of 98 °C for 10 s, 65 °C for 15 s, and 72 °C for 15 s. After respectively 8, 11, and 14 cycles, 10 μl aliquots were collected and run on a pre-cast 6% polyacrylamide gel (Invitrogen) at 145 V for 60 min. The lowest PCR cycle where the 225-bp band representing the RNA–DNA ligated complexes could be visualized was chosen for the final library amplification.

Library amplification and sequencing. A total of four PCR reactions, each using a different barcoded primer, were prepared for each library. After amplification, the four reactions were pooled and run on a pre-cast 6% polyacrylamide gel (Invitrogen) at 145 V for 60 min. The 225-bp band was excised and purified.

Library size was assessed using a High Sensitivity DNA Bioanalyzer Kit (Agilent) and quantified by quantitative PCR using the Library Quantification Kit for Illumina sequencing platforms (KAPA Biosystems) and StepOne Real-Time PCR System (Applied Biosystems). Sequencing was performed with a Single-End 150-bp Kit on the Illumina HiSeq 2500 platform using the sequencing primer 5ʹ-ACACTCTTTCCCTACACGACGCTCTTCCGATCT-3ʹ.

Generation of libraries capturing NPM interactions. NPM libraries were produced following the above protocol for the generation of RADICL-seq libraries with the modifications below. After the purification steps that followed dA-tailing and RNase H reactions, reversal of crosslinks was performed as described above. Samples were concentrated to a final volume of 13.9 µl, followed by the addition of 3 μl of 10× T4 RNA ligase buffer; 1 μl of 20 μM pre-adenylated and biotinylated bridge adaptor; 1 μl of RNaseOut (Thermo Fisher Scientific); 13.3 U/μl T4 RNA ligase 2, truncated KQ (New England Biolabs); and 9 µl of 50% PEG. The mixture was incubated at 20 °C overnight to ligate the pre-adenylated bridge adapter to the 3ʹ-OH of the RNA molecules. Next, bridge adapter-ligated molecules were purified with DNA Clean & Concentrator-5 Kit (Zymo) according to the manufacturer’s instructions and eluted in 50 μl of H_2_O. DNA ligation was carried out by adding 450 μl of 1× T4 DNA ligase buffer with ATP containing 4 U/μl T4 DNA ligase (New England Biolabs) and incubating the solution at room temperature for 4 h. The RNA–DNA complexes were purified with DNA Clean & Concentrator-5 Kit (Zymo) as described above, and then subjected to reverse transcription and subsequent steps of RADICL-seq library preparation and sequencing.

Generation of libraries treated with different enzymes targeting the RNA. Libraries for treatment with different enzymes were produced following the above protocol for the generation of RADICL-seq libraries with the following exceptions: (i) For the library with no enzymatic treatment, the RNase H digestion after chromatin dA-tailing was omitted. (ii) For the library generated with nuclease S1 treatment, prior to the chromatin end-repair step, the purified bead–nuclei pellet was resuspended in a 200 μl solution containing 40 mM sodium acetate (pH 4.5 at 25 °C), 300 mM NaCl, 2 mM ZnSO_4_, and 10 U nuclease S1 (Thermo Fisher Scientific), and then incubated at room temperature for 30 min. The reaction was stopped by adding 5 μl of 10% SDS, and then the RADICL-seq protocol was followed. (iii) For the library generated with RNase V1 treatment, after the chromatin dA-tailing step, each sample was digested with 0.01 U of RNase V1 (Ambion) at room temperature for 30 min. The reaction was stopped by adding 5 μl of 10% SDS, and then the RADICL-seq protocol was followed.

CAGE library preparation. RNAs from nuclear fractions of mESCs and mOPCs were extracted as previously publishedl^11^. CAGE libraries were prepared according to Takahashi et al. ^46^. Briefly, 3 μg of nuclear RNA was used for reverse transcription with random primers. The 5ʹ end of each cDNA–RNA hybrid was biotinylated and captured using magnetic streptavidin-coated beads. After capture, cDNAs from cap-trapped RNAs were released, ligated to 5ʹ-barcoded linkers and digested with *Eco*P15I. The cDNA tags were then ligated with a 3ʹ linker and amplified with nine PCR cycles. Libraries were sequenced on an Illumina HiSeq 2500 system, with 50-bp single-end reads.

### Data analysis

RADICL-seq mapping and processing. RNA and DNA tags at both ends of the adapter were extracted from raw sequencing reads by using TagDust2^47^ (ver. 2.31). Multiplexed reads were split by six nucleotide barcodes embedded in the 3ʹ-linker sequence. Artificial sequences were removed using TagDust^48^ (ver. 1.1.3). We identified and removed rRNAs from the extracted RNA tags using RNAdust^49^ (ver. 1.0.6) with a ribosomal DNA repeating unit (GenBank: BK000964.2). PCR duplicates were removed from paired RNA–DNA tags using FastUniq^50^. We aligned RNA and DNA tags separately to the mouse genome (mm10 assembly) using BWA^51^ (ver. 0.7.15-r1140) with *aln* and *samse* functions. Mapped RNA and DNA tags were paired with unique sequencing read IDs using samtools^52^ (ver. 1.3.1) and bedtools^53^ (ver. 2.17.0). These processes were run on the MOIRAI pipeline platform^54^.

Reference annotation and genome binning. The comprehensive gene annotation for mouse^12^ (GENCODE release M14) was downloaded and genic transcripts (i.e., the RNA products of a gene) were divided into four major groups of biotypes: protein_coding, long_ncRNA (defined according to GENCODE “Long noncoding RNA gene annotation”), ncRNA (defined according to GENCODE “Noncoding RNA predicted using sequences from Rfam and miRBase”), and other (all remaining genes). The mouse genome binning at 25-kb resolution was performed using bedtools^53^ (ver. 2.26.0) with parameter *-w 25000*, and 1 Mb bins for the genomic heatmap were generated using *-w 1000000*.

Annotated feature overlaps. RNA and DNA tags were associated unambiguously with the corresponding annotated features. To achieve that, we identified each fragment by the single-nucleotide position at its center to reduce the possibility of fragments overlapping with multiple genes or bins. The strand information was considered for RNA tags, but discarded for DNA tags. Both RNA and DNA tags were required to map uniquely (Burrows–Wheeler Aligner MAPping Quality (BWA MAPQ) = 37) to the genome.

Reproducibility. The summary of interactions by condition, replicate, and RNA–DNA pairs; the calculation of pairwise Pearson’s correlation coefficients on the counts of complete sets of observations; and the visualization of the results were performed using the CAGEr package^55^.

Correlation with RNA-seq. RADICL-seq interactions were summarized per genic transcripts and normalized to reads per kilobase (RPK) to account for over-representation of longer transcripts. Nuclear and cytosolic RNA-seq (and CAGE-seq) reads were mapped to the mm10 genome using STAR^56^ (ver. 2.5.0a) with default parameters, and low-quality reads (MAPQ <10) were removed. The files were similarly intersected with GENCODE ver. M14 to generate counts and then normalized to transcripts per million (TPM). A lower threshold of 1 was applied to both RPK and TPM to select interacting and expressed transcripts, respectively. The square of the Pearson’s correlation coefficient was used to determine the dependency between variables.

RADICL-seq and GRID-seq robust interactions calculation. *P* value calculations were performed in R using the *binom.test* function with parameters *N*, *P*, and *alternative* *=* “*greater*.” For any given interaction, *N* is the total number of interactions of the RNA involved and *P* is the reciprocal of the total number of unique genomic bins the RNA has been observed to interact with. The *p* value correction was performed using the *p.adjust* function with *method* *=* “*BH*.” Interactions with an adjusted *p* value < 0.05 were deemed significant. The significant datasets were then filtered to remove any RNA–DNA contacts intersecting blacklisted regions for the mm10 genome.

Genome-wide RNA–DNA interaction plots. For the genome-wide interaction plots, the genome was divided into 25-kb bins, and each interaction was assigned to these bins for both the RNA and DNA end of the tag. For each unique combination of the two bins (RNA and DNA coordinates), all interactions were collapsed into one value to avoid overplotting. The plot was produced in R using the ggplot2 package^57^ where each value is colored by the most-represented RNA region or RNA class if more than one individual interaction was assigned to these coordinates. All interactions were then divided into six classes of genomic distance between the RNA and DNA end of the tag, and the proportion of interactions derived from specific RNA regions and RNA classes were visualized in barplots.

RNA–DNA interactions with respect to TAD boundaries. To investigate any relationship between RNA–DNA binding and TAD boundaries, published mESC and mouse neural progenitor cell TADs were retrieved^28^. For all cell types and conditions, the coverage of RNA and DNA tags was visualized at the boundaries of TADs in the form of a metaplot.

To further investigate the effects of TAD boundaries on the spread of RNA–DNA interactions, interactions were split into those that originated within a TAD and those that did not. This was performed on a TAD-by-TAD basis, because interactions that originate outside one TAD may originate within a neighboring TAD. The coverage of DNA-binding locations for each set of interactions was visualized in windows around TADs as metaplots.

The metadata profiles were generated using the ScoreMatrixBin function from the genomation R package^58^. This function divides the genomic regions of interest into an equal number of bins, and then it calculates the mean scores for each bin across regions. In our case, each TAD region was extended by the same size both upstream and downstream, and the resulting region was chopped into 300 bins and averaged per position by the ScoreMatrixBin function to generate the average profile of tags coverage.

Comparison of RADICL-seq data with DRIP-seq data. To compare DRIP-seq and RADICL-seq DNA-binding patterns in the significant datasets, mESC DRIP-seq data were retrieved from the Gene Expression Omnibus (GEO) database (accession no. GSM1720620)^25^. As with RADICL-seq RNA and DNA tags, the average coverage of DRIP-seq reads in bins around TAD boundaries was visualized in the form of a metaplot. To investigate whether there was enrichment for RADICL-seq interactions binding within R-loops, the average coverage of RADICL-seq DNA tags within DRIP-seq peaks was visualized in the form of a metaplot for the total and NPM datasets.

Correlation of differences in gene expression and genome-wide DNA binding profiles between mESCs and mOPCs. To explore the relationship between gene expression and RNA–DNA binding, the binding pattern and expression level of all genes were compared between the mESC and mOPC total datasets. To compare gene expression between the cell types, the difference in normalized counts between cell types was calculated as a percentage of the maximum expression in either condition. To compare the DNA-binding profiles between the cell types, the genome was divided into 5-kb bins, and each bin was assigned a value of 1 or 0 depending on whether there was a DNA tag present in that bin. This was performed for all genes. The binary vectors produced in this way were then compared by calculating the Jaccard distance between the vectors derived from each cell type. The resulting data from each analysis was visualized as a scatterplot.

Comparison of RADICL-seq with RAP-DNA for Malat1. Malat1 RAP-DNA data was obtained from GEO (accession no. GSE55914). The raw reads were processed with TrimGalore!^59^ (ver. 0.4.5) with parameters *--paired --trim1* and aligned to the mm10 genome by using Bowtie2^60^ (ver. 2.3.3.1) with parameters *--no-mixed --no-discordant*. Samtools^52^ (ver. 1.5) was employed to select uniquely mapping fragments and Picard *MarkDuplicates* (ver. 2.9.0.21, http://broadinstitute.github.io/picard/) was used to remove PCR duplicates. Peak calling was performed using macs2^61^ with parameters *--broad --nolambda –nomodel*. We employed *featureCounts*^62^ to summarize the gene reads from RAP-DNA and Malat1-DNA tags from GRID-seq and RADICL-seq. Gene counts were normalized to RPK and sorted according to the normalized values. The top 10,000 genes from each dataset were used to generate the Venn diagram with the *VennDiagram* package^63^ in R. Linear distance to nearest peak was calculated using the peaks output file obtained from macs2 and the *distanceToNearest* function from the *GenomicRanges* package^64^ in R. SPRITE RNA and DNA clusters were retrieved from GEO (accession no. GSE114242), and only DNA coordinates present within the same cluster as Malat1 RNA were selected for the analysis.

Comparison of RADICL-seq with ChIRP-seq for Rn7sk. The list of Rn7sk genomic target locations in the mm9 genome was retrieved from Flynn et al. ^18^. Peak coordinates were lifted from the mm9 genome to the mm10 genome by using *LiftOver*, and genes overlapping these peaks were considered as targeted by Rn7sk. We employed *featureCounts*^62^ to summarize by gene the Rn7sk-DNA tags from GRID-seq and RADICL-seq data. Counts were normalized to count per million and averaged across replicates. The Venn diagram was generated using the *VennDiagram* R package. Linear distance to nearest peak was calculated using the peaks output file obtained from LiftOver and the *distanceToNearest* function from the *GenomicRanges* package^64^ in R. SPRITE RNA and DNA clusters were retrieved from GEO (accession no. GSE114242), and only DNA coordinates present within the same cluster as Rn7sk RNA were selected for the analysis.

Analysis of repetitive elements. The significant RNA–DNA pairs in this dataset were generated by using the same approach described above for the other RADICL-seq significant datasets. RNA and DNA tags with MAPQ ≥37 were processed using bedtools^53^ (ver. 2.26.0) and RepeatMasker^30^ (ver. 4.0.6) to generate the dataset of RADICL-seq RNAs intersecting (with options -s -wb) and not intersecting (with option -v) with REs annotated in the mm10 genome. Self-interactions were removed from the datasets by comparing the GENCODE Gene ID associated with both the RNA and the DNA tags of the same pair. The relative percentage of interactions across different intervals of RNA–DNA distance was calculated by dividing the number of significant interactions in a given RNA–DNA distance interval by the total number of RNA tags intersecting a given repeat family for each experimental condition. *P* values were calculated by two-sided Student’s *t* test.

Transcript-specific genomic interactions. The significant interactions for the transcripts under analysis were summarized by DNA bins, and reported as counts associated with genomic coordinates. The *circlize* package^65^ in R was used to visualize the results genome wide and in greater detail for *cis* interactions, and the log_2_ of counts were used to color the connecting lines of the RNA–DNA pairs.

High-throughput Chromosome Conformation Capture (Hi-C) paired-end reads processing. Publicly available Hi-C sequencing paired-end reads for mESCs and neural progenitor cells were obtained from Bonev et al. ^28^ and processed using the Hi-C User Pipeline (HiCUP)^66^ (ver. 0.5.3). Using this pipeline, we mapped Hi-C paired-end reads to the mm10 genome and filtered reads for expected artifacts resulting from the sonication and ligation steps (e.g., circularized reads, reads with dangling ends) of the Hi-C protocol. Data from different biological replicates were then pooled together. HOMER^67^ (ver. 4.7.2) software was used to filter HiCUP-processed Hi-C paired-end reads with MAPQ = 30 (following recommendations described in Yaffe and Tanay^20^) and other HOMER recommended settings (e.g., PCR duplicates, expected *Hin*dIII restriction sites, self-ligation events). HOMER-filtered Hi-C-mapped paired-end reads were then binned at a fixed resolution of 25 kb and normalized for restriction fragment bias using HOMER’s *simpleNorm* algorithm.

Density plots. Data for density plots were extracted from RADICL-seq RNA and DNA processed data. Random regions were extracted using bedtools *random*. Peak information for histone modifications and DHS-seq and ATAC-seq data was downloaded from ENCODE. Only H3K4me3 and H3K9ac peaks positioned outside the first exon and intron were used for the analysis. Data of density plots were calculated using *makeTagDirectory* followed by *annotatedPeaks* in HOMER^67^ (ver. 4.9) with parameters -size 10000 -hist 1 -histNorm 100. Finally, data were divided by the mean of values of ±5000 bp to align baselines. Plots were generated by ggplot2^57^ with the *geom smooth* option.

Triple helix search. To computationally determine the location of triplex helices in Meg3 and Malat1 *trans* contacts, we used the spliced transcripts ENSMUST00000146701.7 and ENSMUST00000172812.2, respectively. We assessed the enrichment in triple helices in NPM *trans* contacts expanded by 1000 bp over genomic average. To do so, the Triplex Domain Finder (TDF) region test^68^ was used (triple helix parameters: 12 bp, 1 mismatch) with 1000 random genomic background sets.

Distribution of unique interacting RNAs at gene promoters. DNA tags from the mESC and mOPC total datasets were mapped to de novo CAGE-derived gene promoters by using intersectBed in bedtools, allowing for a 2-kb window around the actual promoter coordinates. The number of RNA tags originating from unique genes was then calculated for each promoter. Promoters were ranked from highest to lowest number of unique interactions, and additional information (gene name, nuclear CAGE expression, locus, biotype) was collated for use in further analyses.

CAGE data mapping and processing. Multiplexed sequencing reads were split by barcode sequences. Reads with ambiguous bases “N” were removed, and linker sequences were trimmed from the 3′ end to create reads of length 21–27 nts. The reads were mapped to the mm10 genome (GENCODE release M14) using bowtie^69^ (ver. 1.2.2) with up to two mismatches, only keeping unique alignments with MAPQ = 20. The CAGEr^55^ (ver. 1.20.0) R package was used to extract TSSs, normalize counts, and define transcriptional clusters. Briefly, the most 5′ position of a CAGE tag represents a TSS; the 5′ coordinates were extracted from the tags, and a genome-wide TSS map was generated at single-nucleotide resolution. The raw counts for the CAGE tag starting site were normalized to expression per million tags using a power-law distribution, and used as proxy for the expression of the corresponding genic transcript. We clustered the tags to define distinct CAGE peaks that occurred within 20 bp and expression was summed per transcriptional cluster.

### Reporting summary

Further information on research design is available in the Nature Research Reporting Summary linked to this article.

## Supplementary information

Supplementary Information

Peer Review File

Reporting Summary

## Data Availability

RADICL-seq and CAGE-seq data were deposited in the GEO database under accession number GSE132192. Source Data for the Figures and Supplementary Figures are available in the Supplementary Data file. All other data are available from the authors upon reasonable request.

## Source data

Source Data

## Code availability

The custom scripts for data analysis are available at RADICL_analysis [https://github.com/fagostini/RADICL_analysis].
