# Author Correction: RADICL-seq identifies general and cell type–specific principles of genome-wide RNA-chromatin interactions

**DOI:** 10.1038/s41467-021-23542-w

**Published:** 2021-05-19

**Authors:** Alessandro Bonetti, Federico Agostini, Ana Maria Suzuki, Kosuke Hashimoto, Giovanni Pascarella, Juliette Gimenez, Leonie Roos, Alex J. Nash, Marco Ghilotti, Christopher J. F. Cameron, Matthew Valentine, Yulia A. Medvedeva, Shuhei Noguchi, Eneritz Agirre, Kaori Kashi, Samudyata, Joachim Luginbühl, Riccardo Cazzoli, Saumya Agrawal, Nicholas M. Luscombe, Mathieu Blanchette, Takeya Kasukawa, Michiel de Hoon, Erik Arner, Boris Lenhard, Charles Plessy, Gonçalo Castelo-Branco, Valerio Orlando, Piero Carninci

**Affiliations:** 1 RIKEN Center for Integrative Medical Sciences, Yokohama, Kanagawa 230-0045 Japan; 2 Laboratory of Molecular Neurobiology, Department Medical Biochemistry and Biophysics, Karolinska Institutet, Stockholm, Sweden; 3 The Francis Crick Institute, 1 Midland Road, London, NW1 1AT UK; 4 Department of Medicine (H7), Karolinska Institutet, Stockholm, 141 86 Sweden; 5 Epigenetics and Genome Reprogramming Laboratory, IRCCS Fondazione Santa Lucia, Rome, Italy; 6 Faculty of Medicine, Imperial College London, Institute of Clinical Sciences, London, W12 0NN UK; 7 MRC London Institute of Medical Sciences, London, W12 0NN UK; 8 School of Computer Science, McGill University, Montréal, QC Canada; 9 Department of Biochemistry and Goodman Cancer Research Centre, McGill University, Montréal, QC Canada; 10 Institute of Bioengineering, Research Centre of Biotechnology, Russian Academy of Science, 117312 Moscow, Russia; 11 Department of Computational Biology, Vavilov Institute of General Genetics, Russian Academy of Science, 119991 Moscow, Russia; 12 Department of Biological and Medical Physics, Moscow Institute of Physics and Technology, 141701 Dolgoprudny, Moscow Region Russia; 13 Department of Experimental Oncology, IEO, European Institute of Oncology IRCCS, Milan, Italy; 14 UCL Genetics Institute, University College London, London, WC1E 6BT UK; 15 Okinawa Institute of Science and Technology, Graduate University, 1919-1 Tancha, Onna-son, Kunigami-gun, Okinawa 904-0495 Japan; 16 Sars International Centre for Marine Molecular Biology, University of Bergen, 5008 Bergen, Norway; 17 KAUST Environmental Epigenetics Program, King Abdullah University of Science and Technology (KAUST), Division of Biological Environmental Sciences and Engineering, 23955-6900 Thuwal, Saudi Arabia

**Keywords:** Biological techniques, Chromatin

Correction to: *Nature Communications* 10.1038/s41467-020-14337-6, published online 24 February 2020.

The original version of this Article contained an error in the author affiliations.

Affiliation 13 incorrectly read ‘Department of Experimental Oncology, European Institute of Oncology, Milan, Italy’ instead of the correct ‘Department of Experimental Oncology, IEO, European Institute of Oncology IRCCS, Milan, Italy’.

This has now been corrected in both the PDF and HTML versions of the Article.

