# Supplementary material for: RADICL-seq identifies general and cell type–specific principles of genome-wide RNA-chromatin interactions
Source: Nat Commun. 2020 Feb 24;11:1018. doi: 10.1038/s41467-020-14337-6 (PMC7039879; doi:10.1038/s41467-020-14337-6)
Supplement: Supplementary file 1 — Supplementary Information [file 41467_2020_14337_MOESM1_ESM.pdf]

Supplementary information for:

RADICL-seq identifies general and cell type–specific principles of genome-wide RNA-chromatin interactions

Alessandro Bonetti[1,2,+,*], Federico Agostini[3,+], Ana Maria Suzuki[1,4], Kosuke Hashimoto[1], Giovanni Pascarella[1], Juliette Gimenez[5], Leonie Roos[6,7], Alex J. Nash[6,7], Marco Ghilotti[1], Christopher JF Cameron[8,9], Matthew Valentine[1], Yulia A Medvedeva[10,11,12], Shuhei Noguchi[1], Eneritz Agirre[2], Kaori Kashi[1], Samudyata[2], Joachim Luginbuehl[1], Riccardo Cazzoli[13], Saumya Agrawal[1], Nicholas M Luscombe[3,14,15], Mathieu Blanchette[8], Takeya Kasukawa[1], Michiel De Hoon[1], Erik Arner[1], Boris Lenhard[6,7,16], Charles Plessy[1], Gonçalo Castelo-Branco[2], Valerio Orlando[5,17,*], Piero Carninci[1,*]

[+]These authors contributed equally to the work.

*Correspondence should be addressed to:

Alessandro Bonetti, Valerio Orlando & Piero Carninci

alessandro.bonetti@riken.jp, valerio.orlando@kaust.edu.sa, piero.carninci@riken.jp

[1]RIKEN Center for Integrative Medical Sciences, Yokohama, Kanagawa, 230-0045 Japan.

[2]Laboratory of Molecular Neurobiology, Department Medical Biochemistry and Biophysics, Karolinska Institutet, Stockholm, Sweden.

[3]The Francis Crick Institute, 1 Midland Road, London NW1 1AT, UK.

[4]Department of Medicine (H7), Karolinska Institutet, Stockholm 141 86, Sweden

[5]Epigenetics and Genome Reprogramming Laboratory, IRCCS Fondazione Santa Lucia, Epigenetics and Genome Reprogramming, Rome, Italy.

[6]Institute of Clinical Sciences, Faculty of Medicine, Imperial College London, London W12 0NN, UK.

[7]MRC London Institute of Medical Sciences, London W12 0NN, UK.

[8]School of Computer Science, McGill University, Montréal, Québec, Canada.

[9]Department of Biochemistry and Goodman Cancer Research Centre, McGill University, Montréal, Québec, Canada.

[10]Institute of Bioengineering, Research Centre of Biotechnology, Russian Academy of Science, 117312 Moscow, Russia.

[11]Department of Computational Biology, Vavilov Institute of General Genetics, Russian Academy of Science, 119991 Moscow, Russia.

[12]Department of Biological and Medical Physics, Moscow Institute of Physics and Technology, 141701, Dolgoprudny, Moscow Region, Russia.

[13]European Institute of Oncology, Department of Experimental Oncology, Milan, Italy.

[14]UCL Genetics Institute, University College London, London WC1E 6BT, UK.

[15]Okinawa Institute of Science & Technology Graduate University, 1919-1 Tancha, Onna-son, Kunigami-gun, Okinawa 904-0495, Japan.

[16]Sars International Centre for Marine Molecular Biology, University of Bergen, N-5008 Bergen, Norway.

[17]King Abdullah University of Science and Technology (KAUST), Division of Biological Environmental Sciences and Engineering, KAUST Environmental Epigenetics Program, 23955-6900 Thuwal, Saudi Arabia.

a

Enzymatic treatment	Raw reads	rRNA-containing (% of input)	Uniquely mapped (% of input)
No treatment	2.2 M	0.8 M (39%)	0.3 M (14%)
Nuclease S1	2.2 M	0.9 M (43%)	0.4 M (16%)
RNase V1	2.9 M	1.4 M (49%)	0.5 M (17%)
RNase H	3 M	0.9 M (30%)	0.8 M (26%)

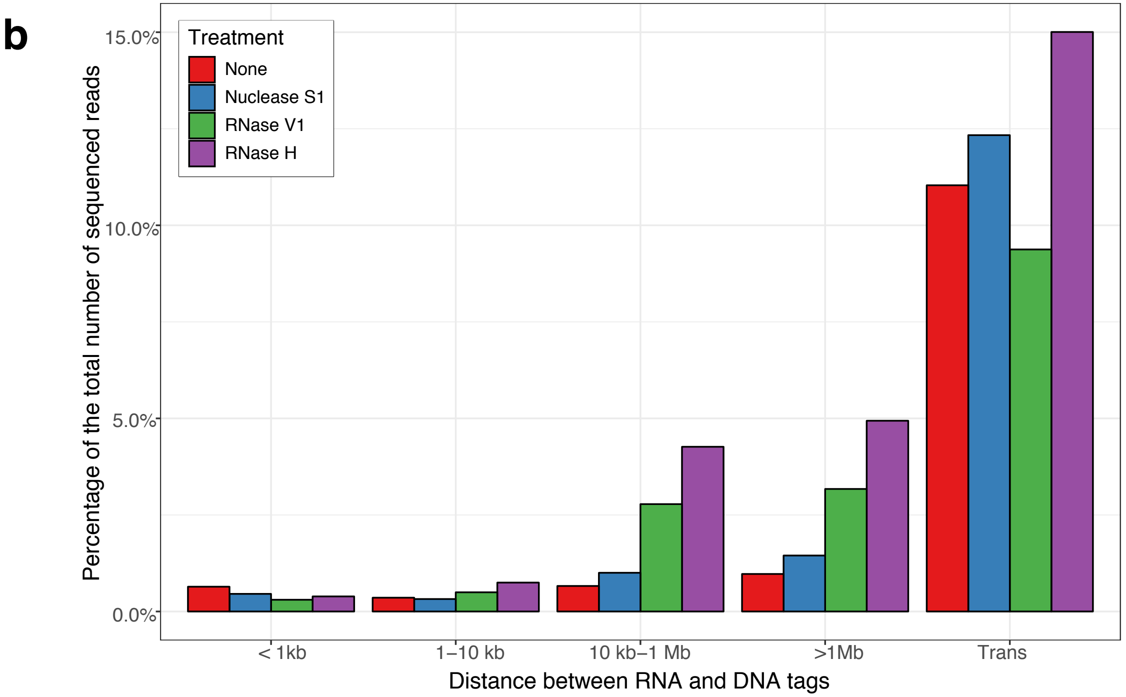

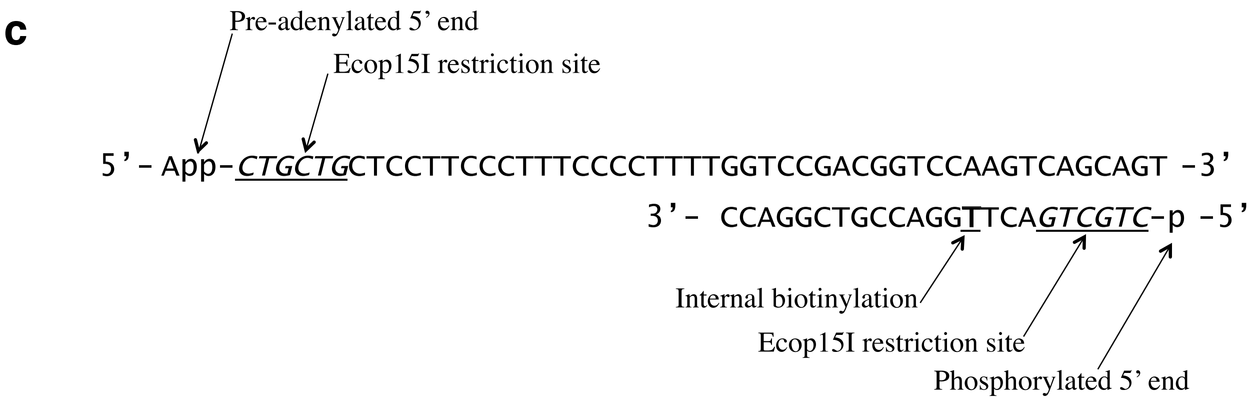

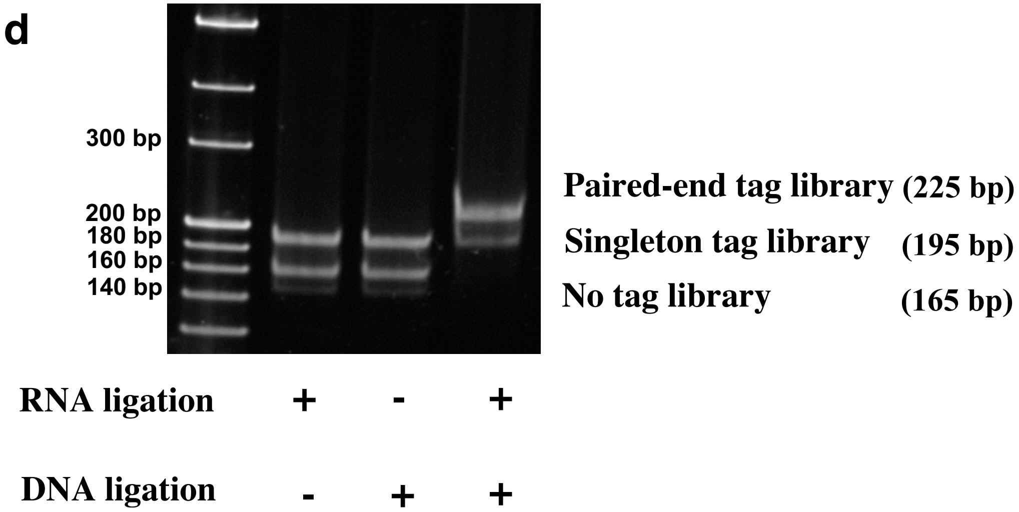

Supplementary Fig. 1. Characterization of RADICL-seq technology. a) Summary statistics of the sequencing results for the datasets of tests of various enzymatic treatments of mESCs. M, million. b) Distribution of the linear genomic distance between RNA and DNA tags derived from the same read after the above enzymatic treatments. c) Sequence and features of the bridge adapter. The adapter contains a 5′ adenylated end (App), an internal T residue with biotin modification (bold) and two restriction sites for EcoP15I. The 3′-overhanging T allows DNA ligation with dA-tailed genomic fragments. d). Generation of RADICL-seq libraries and controls. Gel migration pattern of the PCR-amplified RADICL-seq library compared with controls generated by omitting either DNA ligase or RNA ligase. The expected band for a paired-end tag library was detected only when both RNA and DNA ligases were used for the library construction.

a

Experimental condition	Biological replicate	Raw reads	rRNA	Multi-mapping	Uniquely mapped
1FA	n1	141.3M	42.1M	20.4M	18.2M
1FA	n2	120.5M	46.9M	27.9M	21.9M
1FA	n3	103.8M	37.5M	23.3M	19.7M
2FA	n1	141.8M	31.4M	17.9M	14.9M
2FA	n2	107.4M	44.5M	19.8M	15.8M
2FA	n3	97.6 M	26.9M	20.9M	17.3M

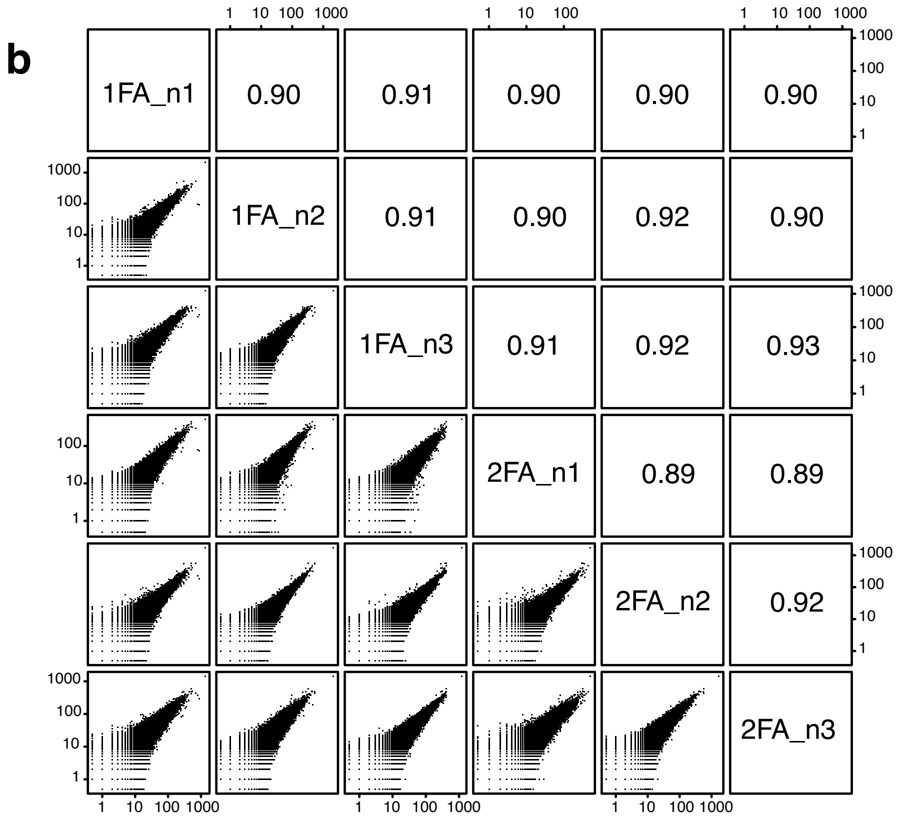

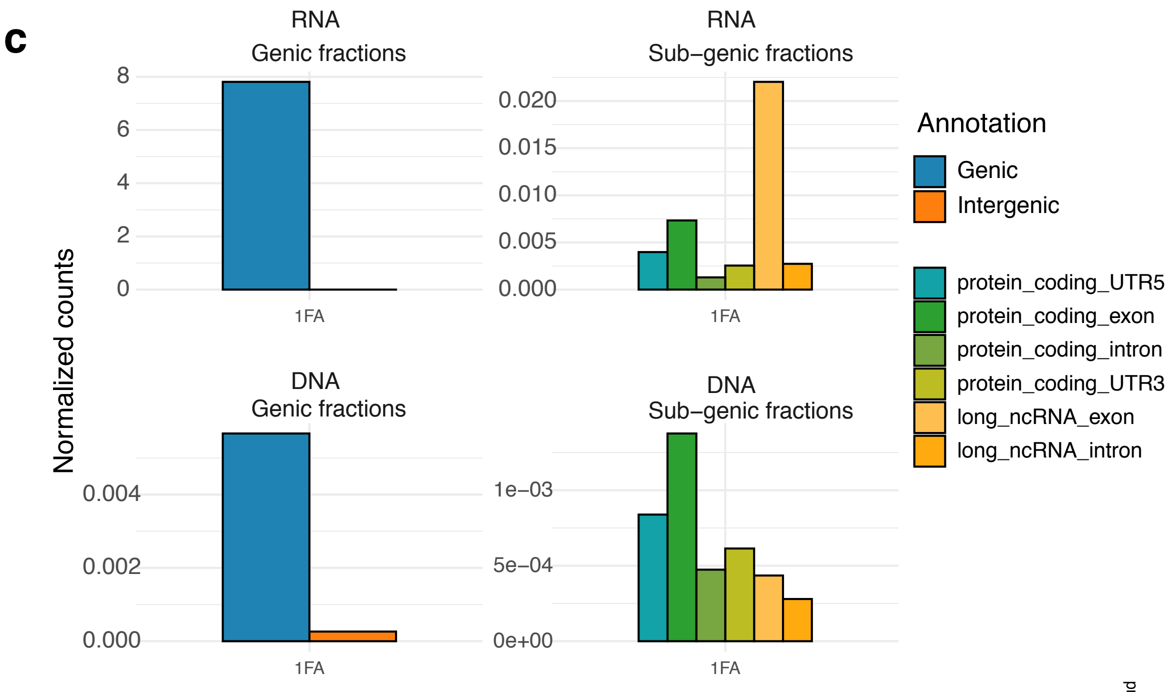

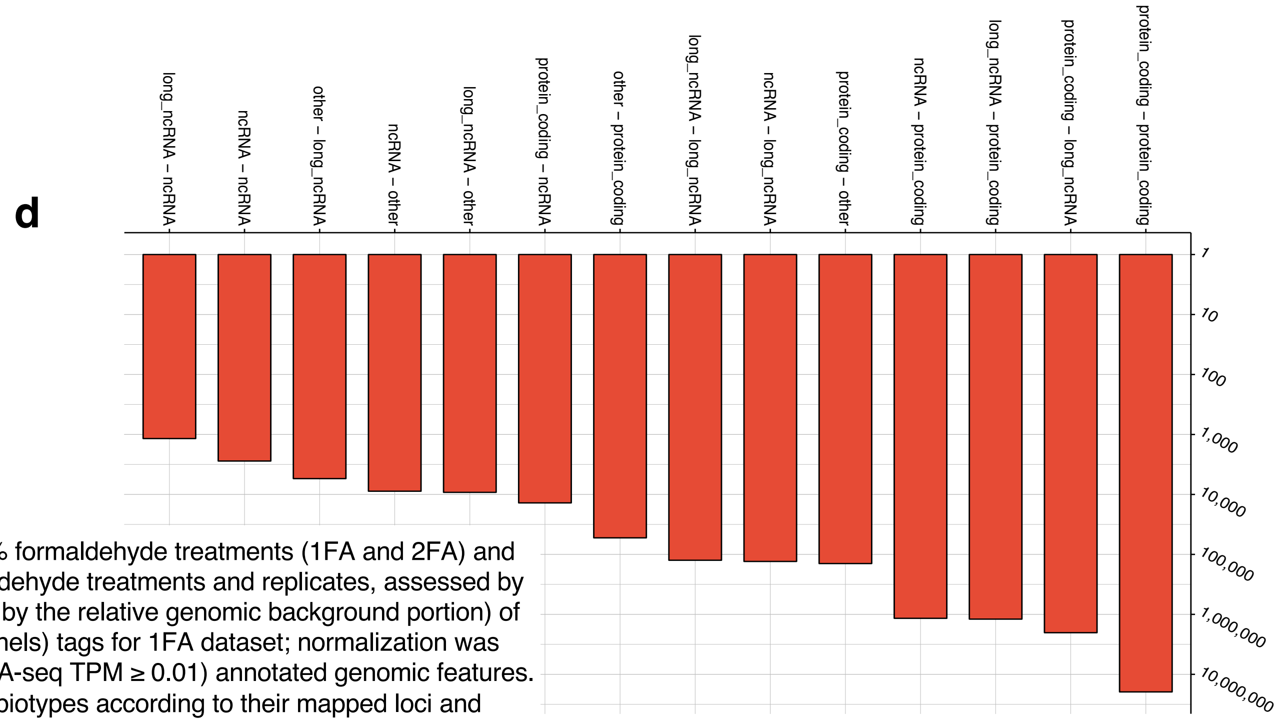

Supplementary Fig. 2. Features of RADICL-seq libraries. a) Summary statistics of the datasets for 1% and 2% formaldehyde treatments (1FA and 2FA) and replicate sequencing results. M, million. b) Reproducibility of the RNA-DNA interaction frequencies across formaldehyde treatments and replicates, assessed by counting the occurrences of genic transcripts and 25-kb genomic bin pairs. c) Normalized counts (counts divided by the relative genomic background portion) of genic and intergenic (left panels) and sub-genic (right panels) fractions of RNA (top panels) and DNA (bottom panels) tags for 1FA dataset; normalization was performed by dividing the interaction count by the sum of the widths of all (DNA) or expressed (RNA; nuclear RNA-seq TPM ≥ 0.01) annotated genomic features. Regions that overlapped by more than one feature were counted only once. d) Distribution of RNA and DNA tag biotypes according to their mapped loci and gene annotation in 1FA dataset. All panels were generated using the raw datasets (not selected for significant interactions).

a

Experimental condition	Biological replicate	Raw reads	rRNA-containing read pairs	Multi-mapping read pairs	Uniquely mapped read pairs
ActD	n1	80.8 M	34 M	12.2 M	9.3 M
ActD	n2	46.4 M	19.7 M	7 M	5.4 M
NPM	n1	185.4 M	86.4 M	28.9 M	14.2 M
NPM	n2	101.2 M	50.3 M	17.6 M	13.9 M

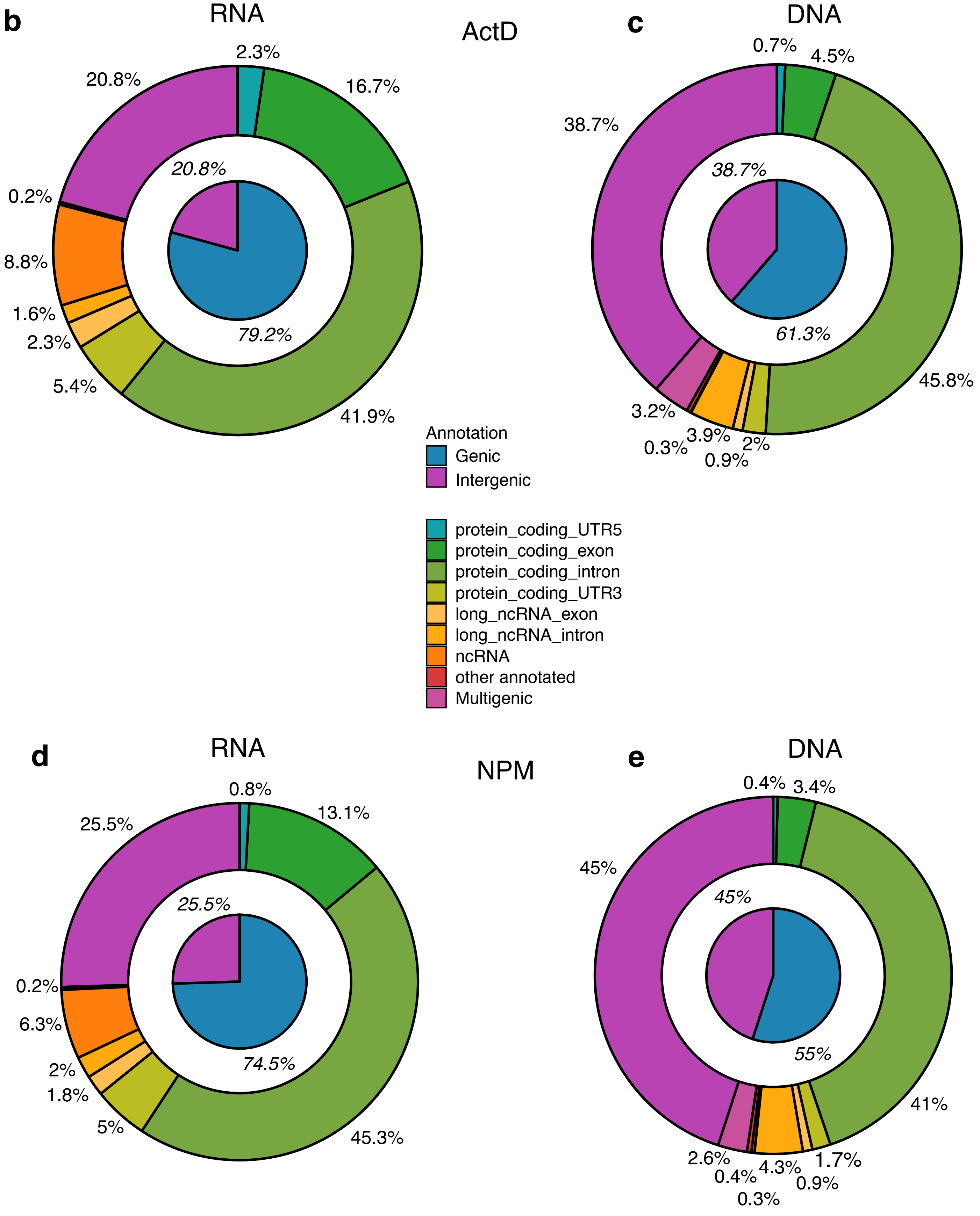

Supplementary Fig. 3. Features of RADICL-seq libraries from ActD-treated and NPM datasets. a) Summary statistics of the ActD-treated and non-protein–mediated (NPM) datasets and replicate sequencing results. M, million. b,d) RNA and c,e) DNA tag origins in b,c) ActD-treated and d,e) NPM datasets. The inner pie charts represent a broader classification into intergenic and genic (annotated genes), while the outer circles show a finer classification of the genic portion. All panels were generated using the raw datasets.

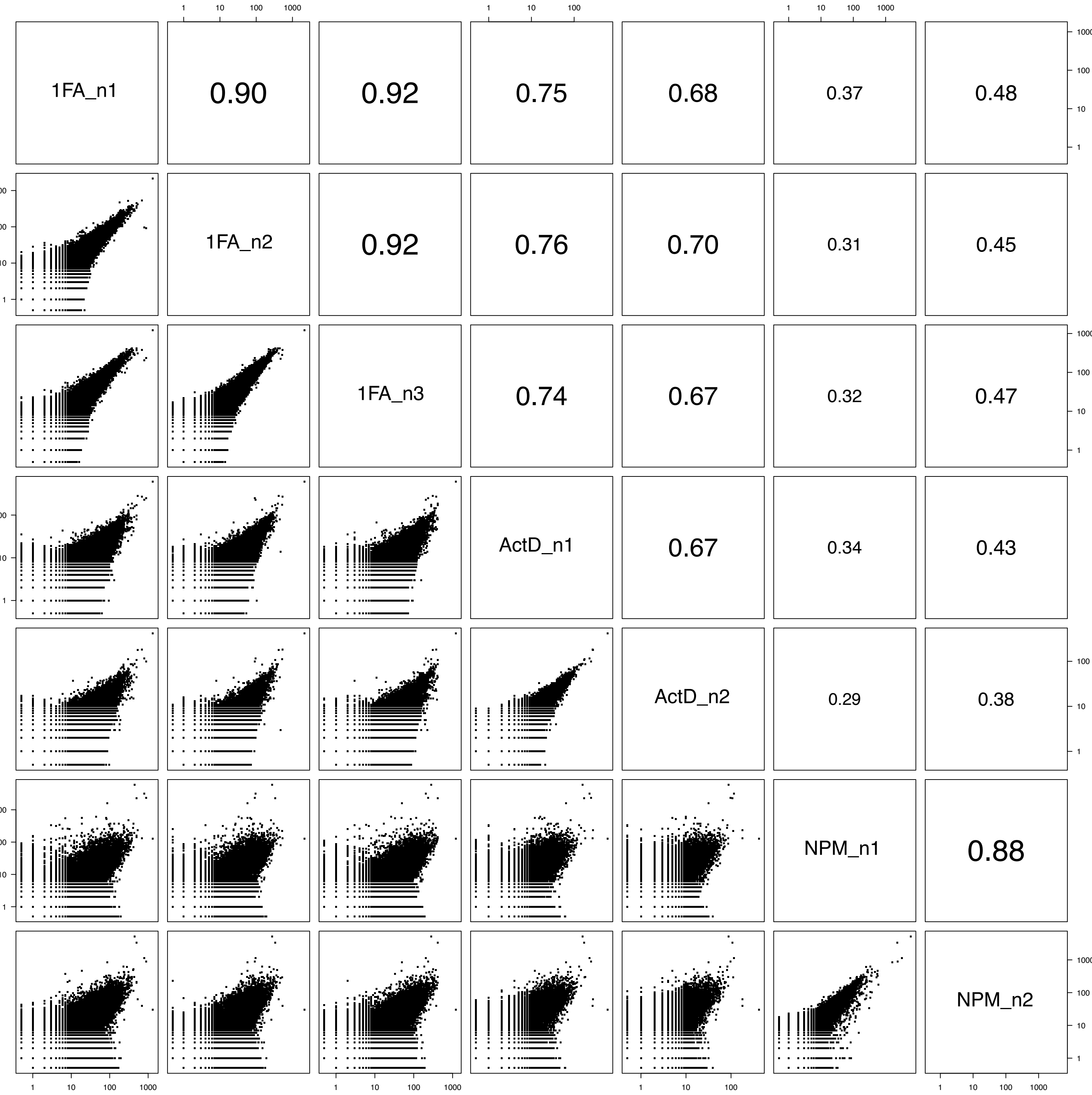

Supplementary Fig. 4. Reproducibility of RADICL-seq libraries among total, ActD, and NPM datasets. Reproducibility of the RNA-DNA interaction frequencies across total (1FA), ActD, and NPM replicates, assessed by counting the occurrences of genic transcripts and 25-kb genomic bin pairs. The plot was generated using the raw datasets.

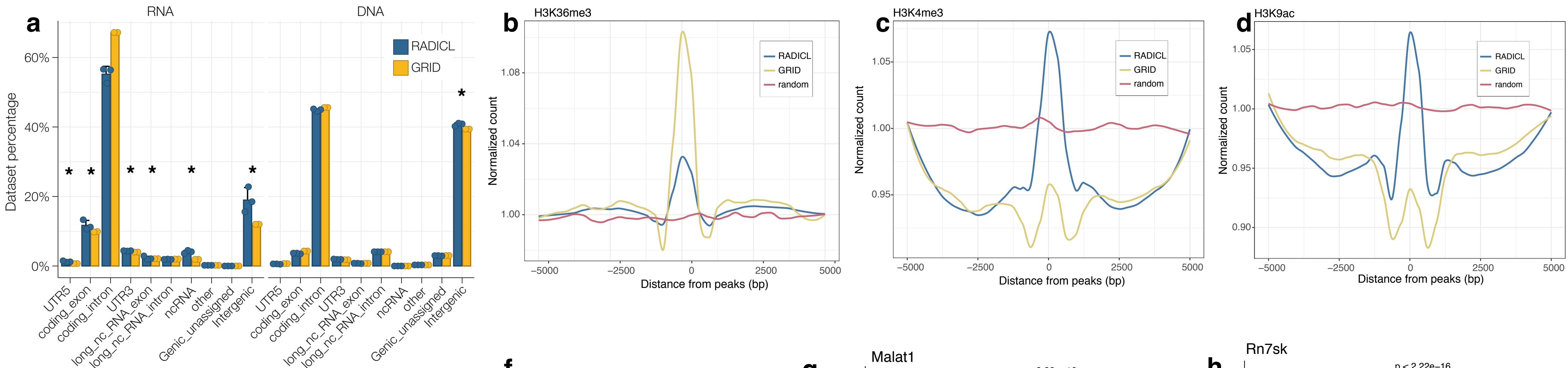

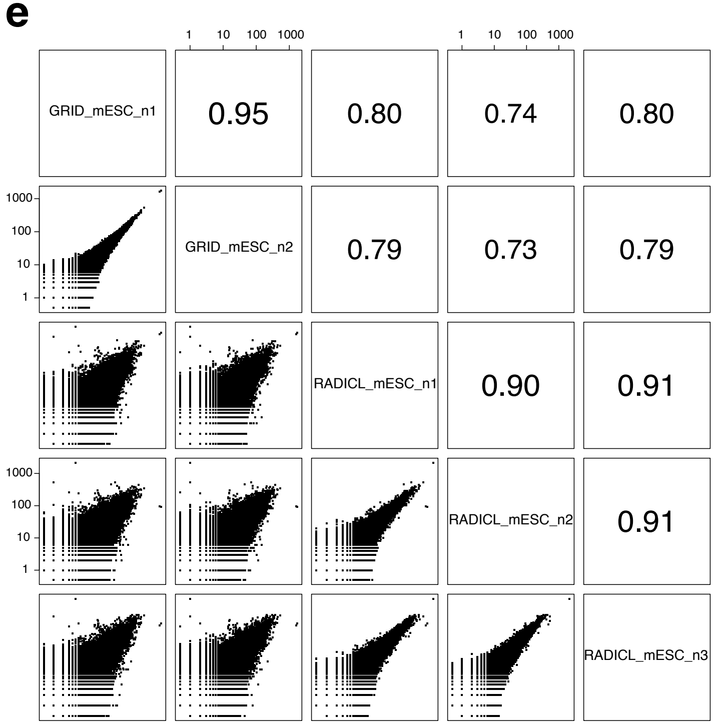

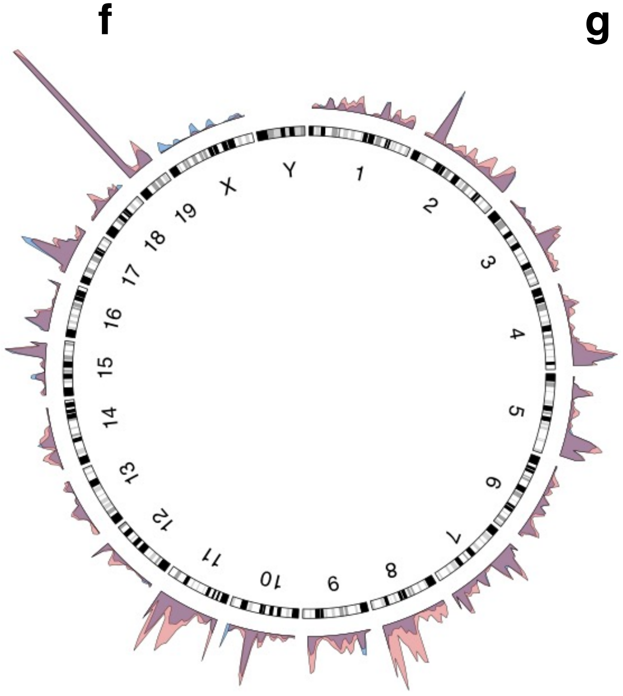

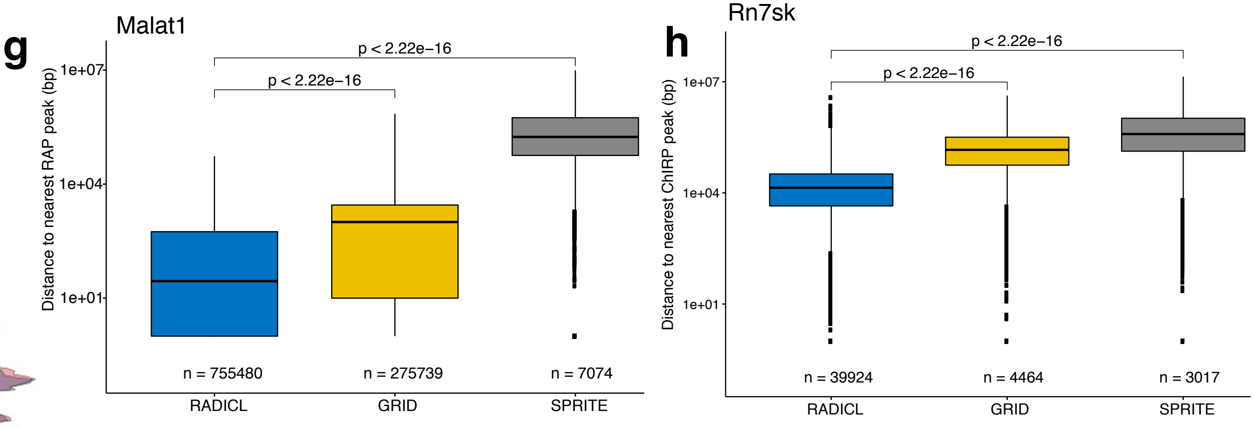

Supplementary Fig. 5. Comparison between RADICL-seq and other technologies. a) Distribution of RADICL-seq (blue) and GRID-seq (yellow) RNA (left panel) and DNA (right panel) tags across biotypes and genic features; data are presented as mean ± s.d; statistical significance was calculated with one-sided (H_0: RADICL < GRID) two-proportions z-test; * = P≤0.05. b–d) RADICL-seq (blue) and GRID-seq (yellow) tag densities around H3K36me3 (b), H3K4me3 (c), and H3K9ac (d) ChIP-seq peaks. The red line represents the density of tags around randomly selected positions of the genome. e) Reproducibility of the RNA-DNA interaction frequencies across 1% FA RADICL-seq and GRID-seq replicates, assessed by counting the occurrences of genic transcripts and 25-kb genomic bin pairs. f) Densities of RADICL-seq (red) and RAP-DNA (blue) of Malat1 RNA-chromatin interactions. All panels were generated using the total raw RADICL-seq dataset. g) Genomic linear distance to nearest Malat1 RAP peak and h) to nearest Rn7sk ChIRP peak of respectively Malat1 and Rn7sk interacting DNA tags detected with RADICL-seq (blue), GRID-seq (yellow) and SPRITE (grey); the number of DNA tags for the set are reported below the box; statistical significance was calculated with two-sided Wilcoxon-Mann-Whitney test.

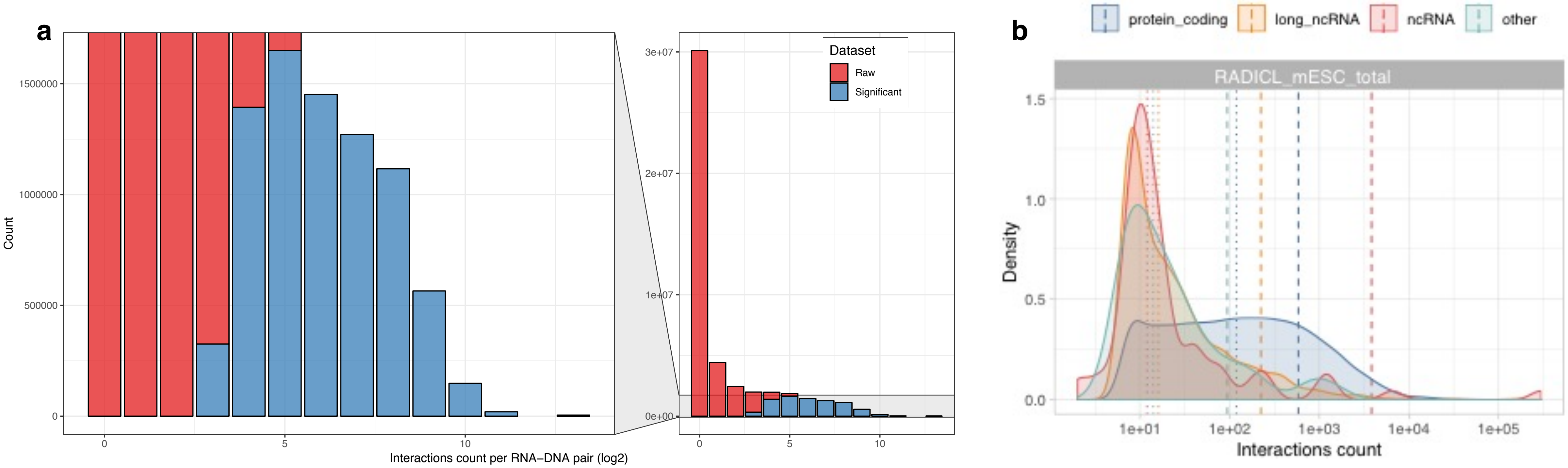

c

	**Raw dataset**			**Significant dataset**		
**Dataset**	**Uniquely mapped interactions**	**Cis** ***(% total)***	**Trans** ***(% total)***	**Uniquely mapped interactions**	**Cis** ***(% total)***	**Trans** ***(% total)***
Total	49,914,160	17,523,875 *(35.1%)*	32,390,285 *(64.9%)*	8,420,123	7,954,089 *(94.5%)*	466,034 *(5.5%)*
ActD	12,002,693	2,936,878 *(24.5%)*	9,065,815 *(75.5%)*	724,021	664,853 *(91.8%)*	59,168 *(8.2%)*
NPM	21,489,520	2,331,587 *(10.8%)*	19,157,933 *(89.2%)*	1,136,020	1,129,456 *(99.4%)*	6,564 *(0.6%)*

d

**Condition**	**Biotype**	**Raw dataset**	**Significant dataset**	Percentage
Total	Protein-coding	20,684	12,441	60.2%
Total	Long_ncRNAs	10,480	1,430	13.7%
Total	ncRNAs	2,427	81	3.3%
Total	Other	5,831	49	0.8%
ActD	Protein-coding	20,034	8,053	40.2%
ActD	Long_ncRNAs	9,523	279	2.9%
ActD	ncRNAs	1,454	30	2.1%
ActD	Other	3,646	8	0.2%
NPM	Protein-coding	20,479	10,702	52.3%
NPM	Long_ncRNAs	10,248	762	7.4%
NPM	ncRNAs	1,950	130	6.7%
NPM	Other	4,969	33	0.7%

Supplementary Fig. 6. Comparison between raw and significant datasets in mESCs. a) Interaction counts distribution in the total dataset before (raw; red) and after the significance calculation and filtering (significant; blue). b) Distribution of genic transcript interaction counts across biotype groups for the significant total dataset; the vertical lines represent the mean (dashed) and median (dotted) of each biotype group. c) Summary of the number and types of interactions for the indicated raw and significant datasets. d) Summary of the number and types of RNA biotypes for the indicated raw and significant datasets.

a

Experimental condition	Biological replicate	Raw reads	rRNA-containing read pairs	Multi-mapping read pairs	Uniquely mapped read pairs
1FA	n1	109.6 M	12.4 M	15.9 M	15.4 M
1FA	n2	177.1 M	23.4 M	20.2 M	17.6 M
1FA	n3	116.7 M	21.1 M	19.5 M	16.4 M

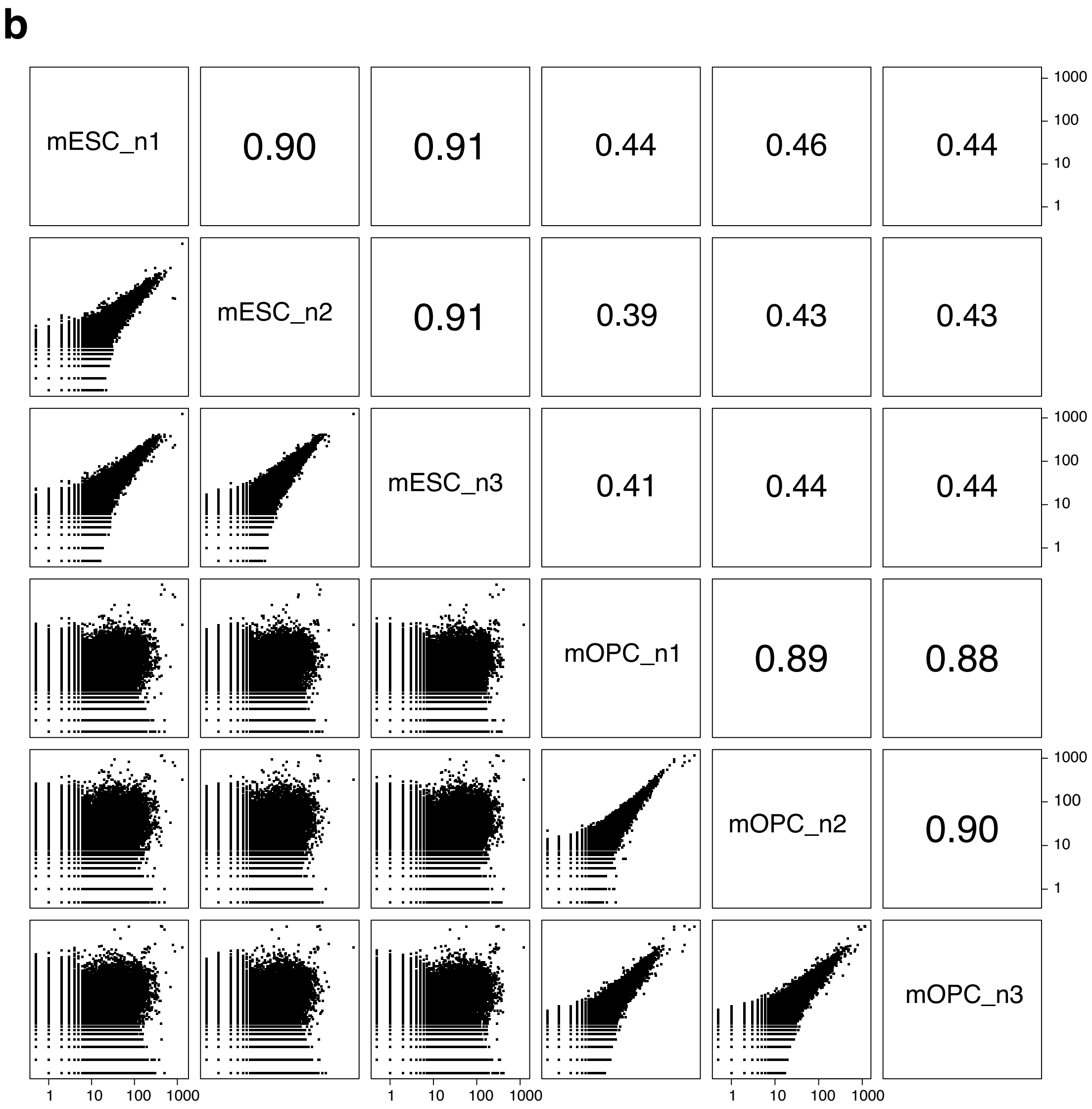

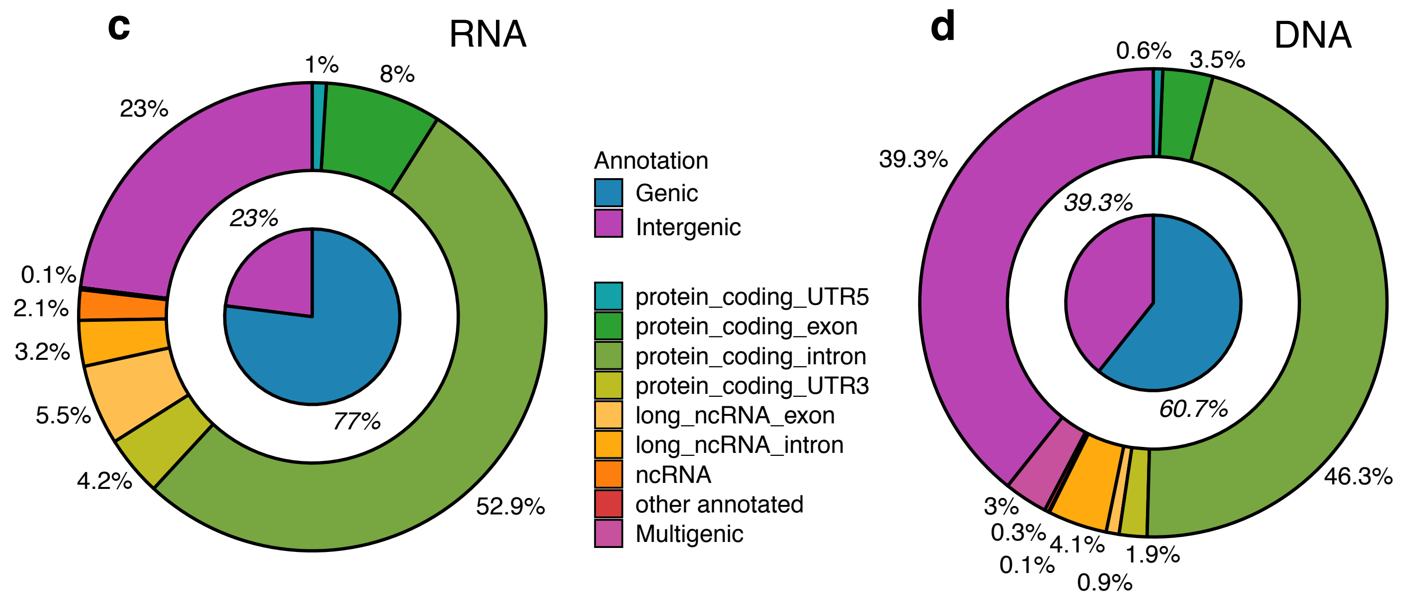

Supplementary Fig. 7. Features of RADICL-seq libraries from mOPCs. a) Summary statistics of the sequencing results for total mOPC RADICL-seq replicates. M, million. b) Reproducibility of the RNA-DNA interaction frequencies across total replicates in mESCs and mOPCs, assessed by counting the occurrences of genic transcripts and 25-kb genomic bin pairs. c) RNA and d) DNA tag origins in mOPC. The inner pie charts represent a broader classification into intergenic and genic (annotated genes), while the outer circles show a finer classification of the genic portion. All panels were generated using the raw datasets (not selected for significant interactions).

a

Experimental condition	Biological replicate	Raw reads	rRNA-containing read pairs	Multi-mapping read pairs	Uniquely mapped read pairs
NPM	n1	183.4 M	54.2 M	35.2 M	24.3 M
NPM	n2	83.2 M	3.5 M	29.3 M	25.5 M

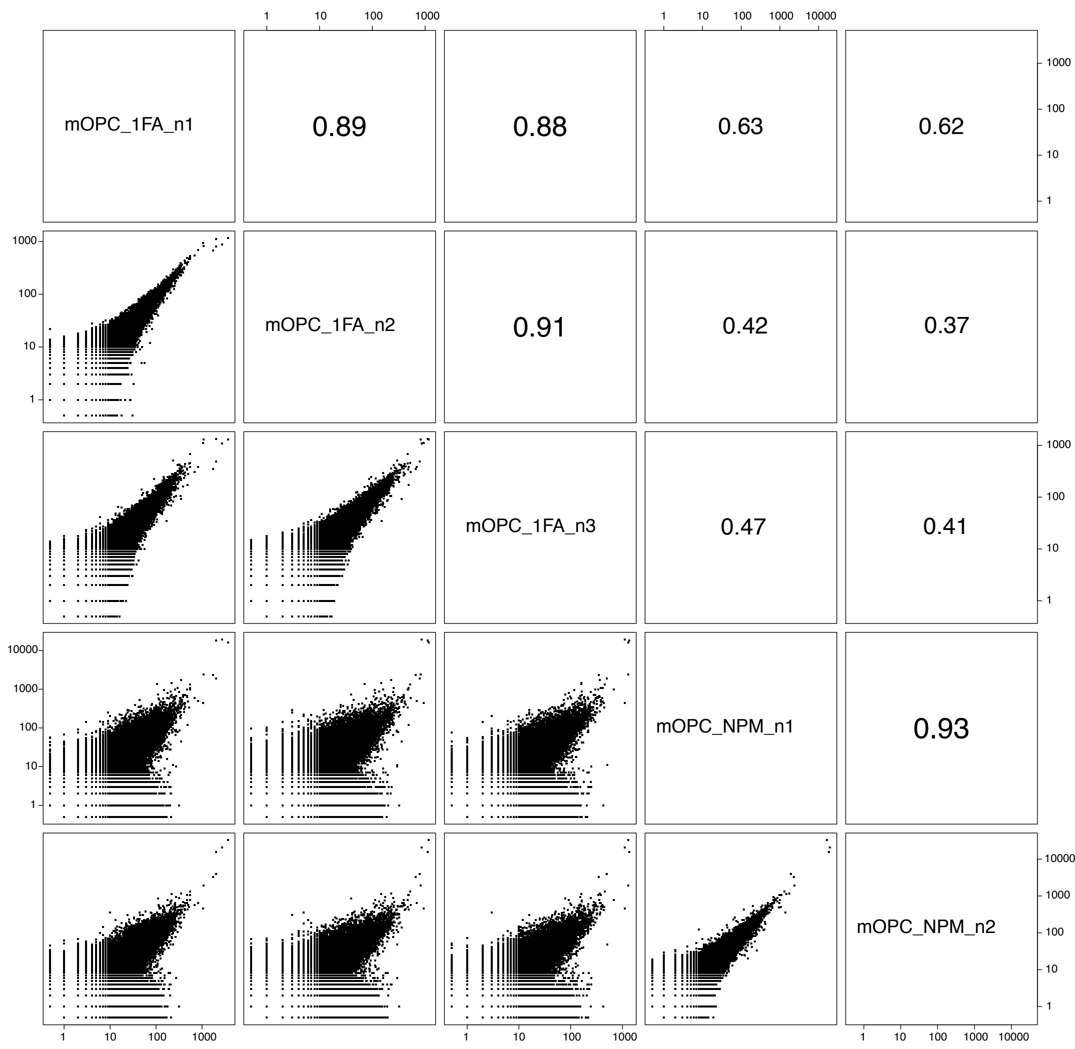

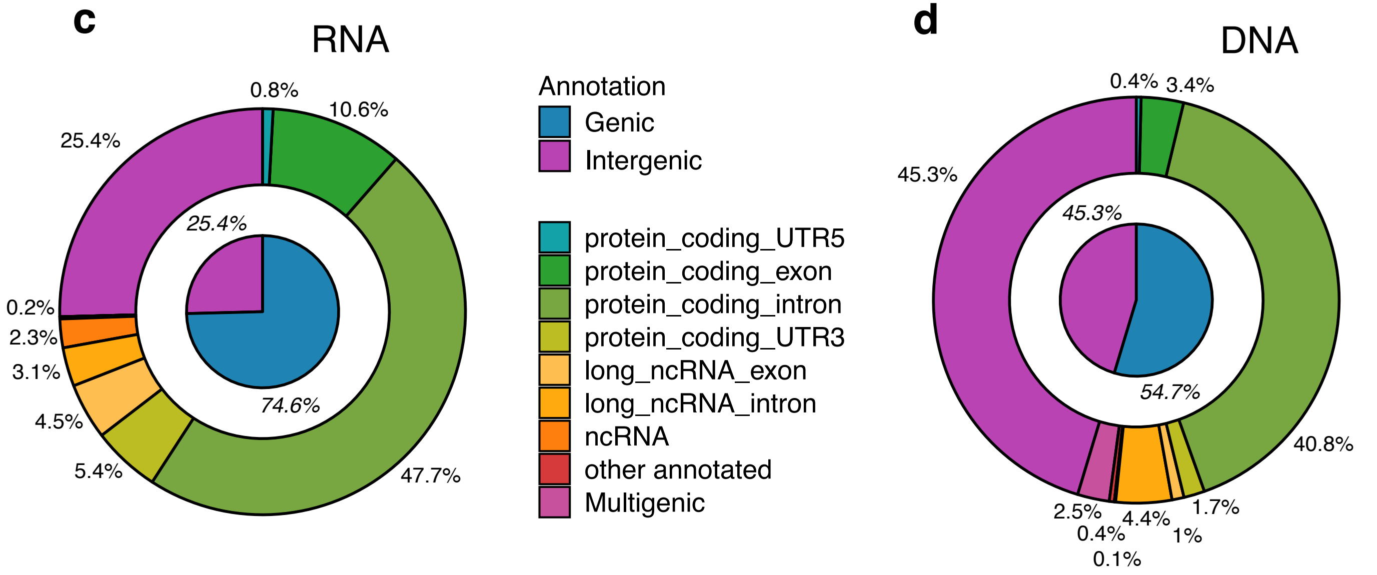

Supplementary Fig. 8. Features of RADICL-seq libraries from the NPM condition in mOPCs. a) Summary statistics of sequencing results for the mOPC RADICL-seq non-protein–mediated (NPM) replicates. b) Reproducibility of the RNA-DNA interaction frequencies across total and NPM replicates in mOPCs, assessed by counting the occurrences of genic transcripts and 25-kb genomic bin pairs. c) RNA and d) DNA tag origins in mOPC NPM samples. The inner pie charts represent a broader classification into intergenic and genic (annotated genes), while the outer circles show a finer classification of the genic portion. All panels were generated using the raw datasets.

a

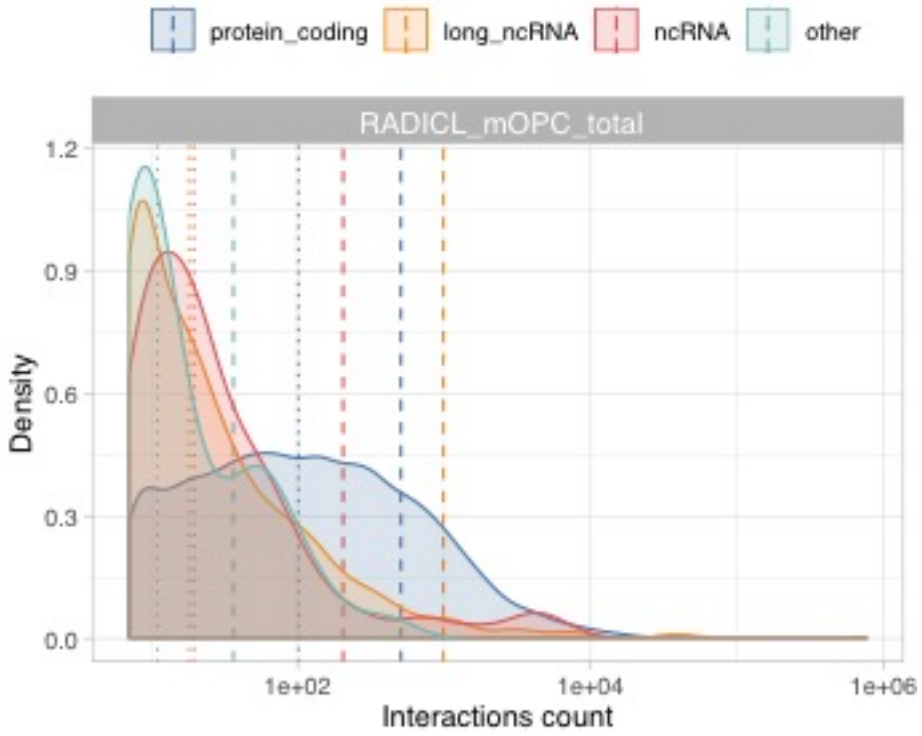

b

	Raw dataset			Significant dataset		
**Dataset**	**Uniquely mapped interactions**	**Cis** ***(% total)***	**Trans** ***(% total)***	**Uniquely mapped interactions**	**Cis** ***(% total)***	**Trans** ***(% total)***
Total	38,068,124	13,148,185 *(34.5%)*	24,919,939 *(65.5%)*	6,765,064	5,933,461 *(87.7%)*	466,034 *(12.3%)*
NPM	37,140,198	4437608 (12%)	32,702,590 *(88%)*	2,500,559	2,386,440 *(95.4%)*	114,119 *(0.6%)*

c

**Condition**	**Biotype**	**Raw dataset**	**Significant dataset**	Percentage
Total	Protein-coding	20,497	10,325	50.4%
Total	Long_ncRNAs	10,418	1,101	10.6%
Total	ncRNAs	2,181	96	4.4%
Total	Other	5,237	41	0.8%
NPM	Protein-coding	20,583	12,434	60.4%
NPM	Long_ncRNAs	10,488	1,970	18.8%
NPM	ncRNAs	2,267	114	5.1%
NPM	Other	5,832	81	1.4%

Supplementary Fig. 9. Comparison between raw and significant datasets in mOPCs. a) Distribution of genic transcript interaction counts across biotype groups for the significant total dataset; the vertical lines represent the mean (dashed) and median (dotted) of each biotype group. b) Summary of the number and types of interactions for the raw and significant datasets. c) Summary of the number and types of RNA biotypes for the raw and significant datasets.

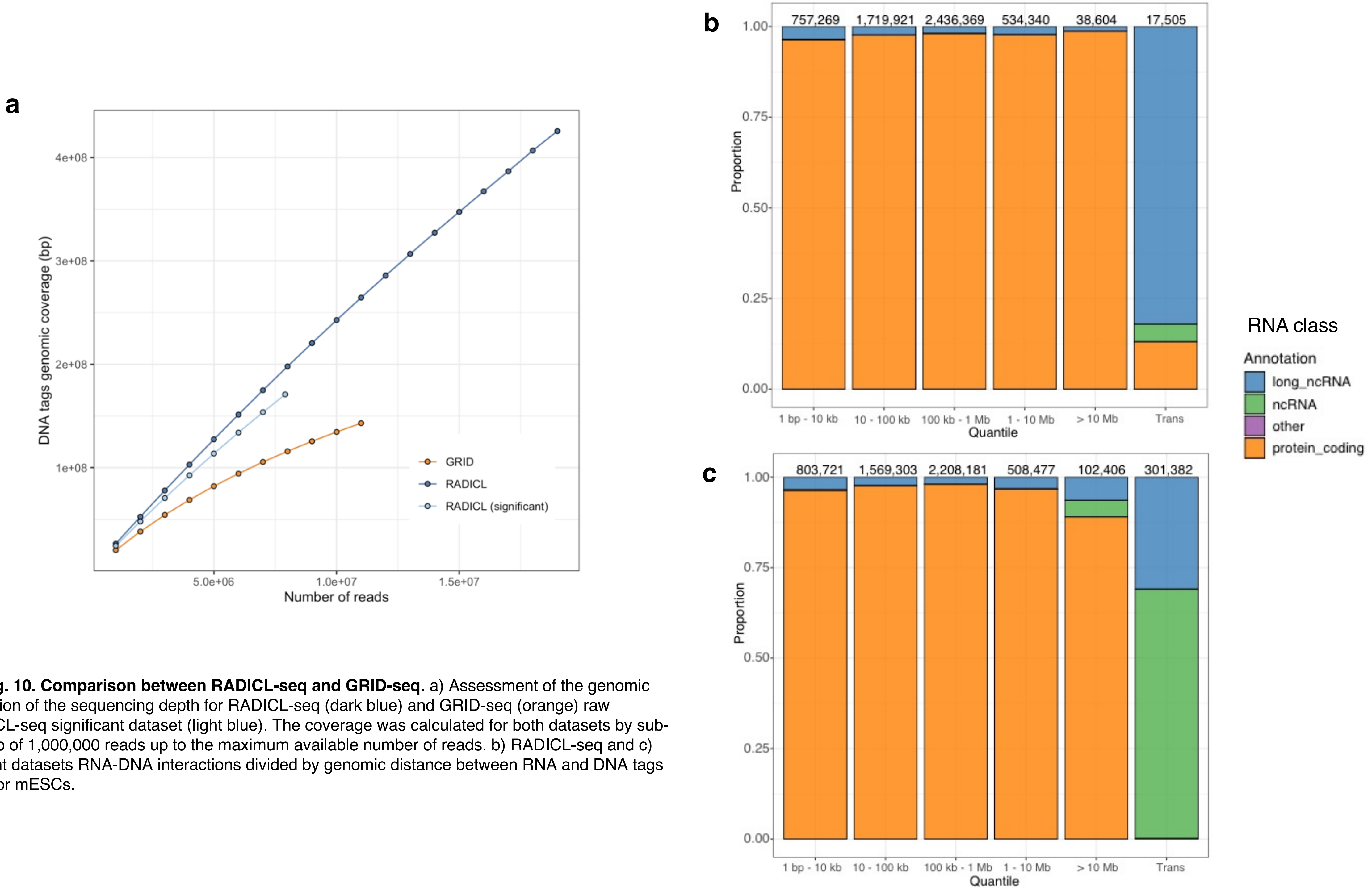

Supplementary Fig. 10. Comparison between RADICL-seq and GRID-seq. a) Assessment of the genomic coverage as a function of the sequencing depth for RADICL-seq (dark blue) and GRID-seq (orange) raw datasets and RADICL-seq significant dataset (light blue). The coverage was calculated for both datasets by sub-sampling with a step of 1,000,000 reads up to the maximum available number of reads. b) RADICL-seq and c) GRID-seq significant datasets RNA-DNA interactions divided by genomic distance between RNA and DNA tags and by RNA class for mESCs.

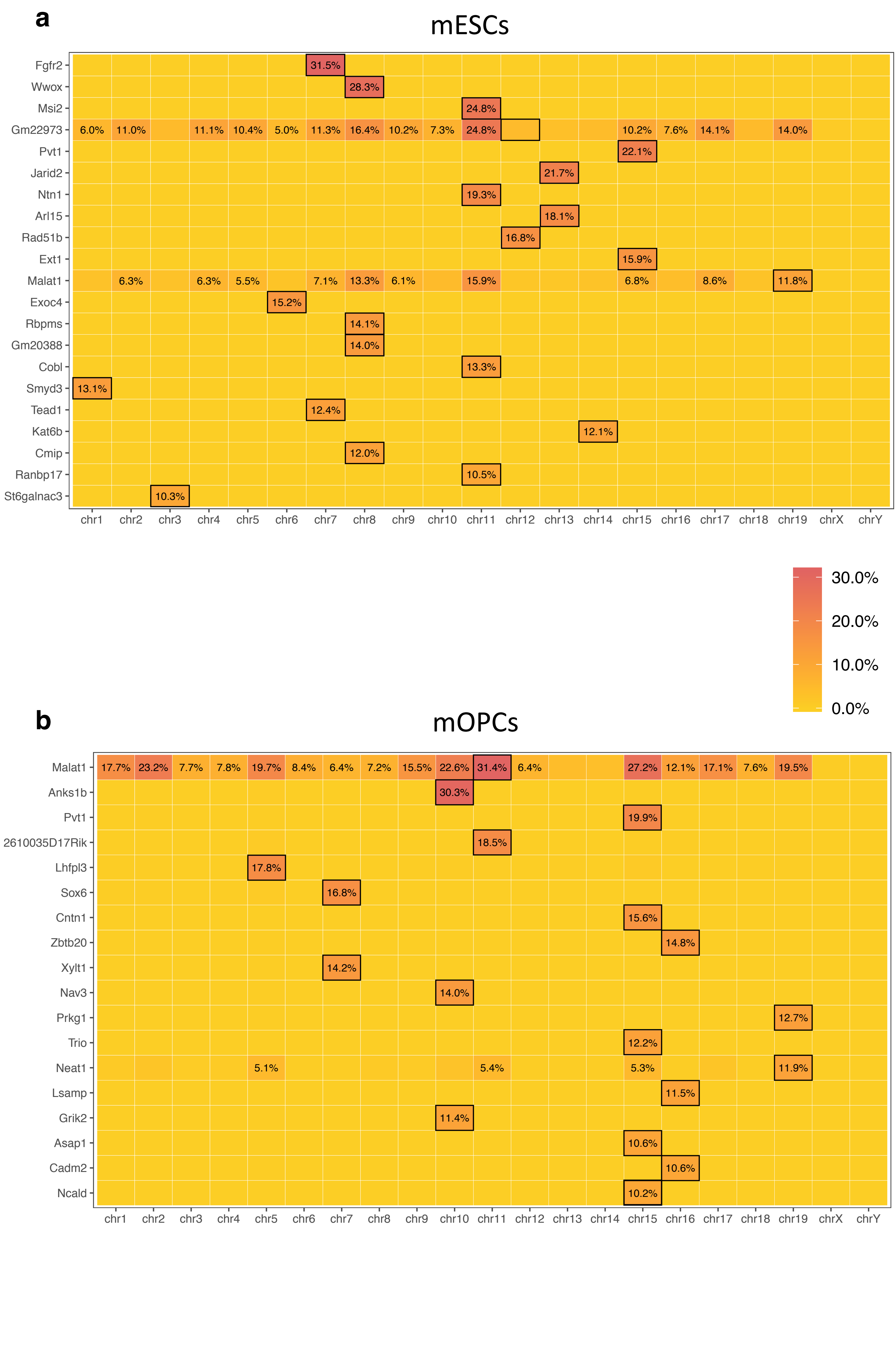

Supplementary Fig. 11. Chromosome-wide binding of selected transcripts. a) mESC and b) mOPC RADICL-seq heatmaps showing the percentage of loci covered on each chromosome by the most interacting (≥10% in at least one chromosome) RNAs. The chromosome of origin for each RNA is highlighted by a black box. All panels were generated using significant total datasets.

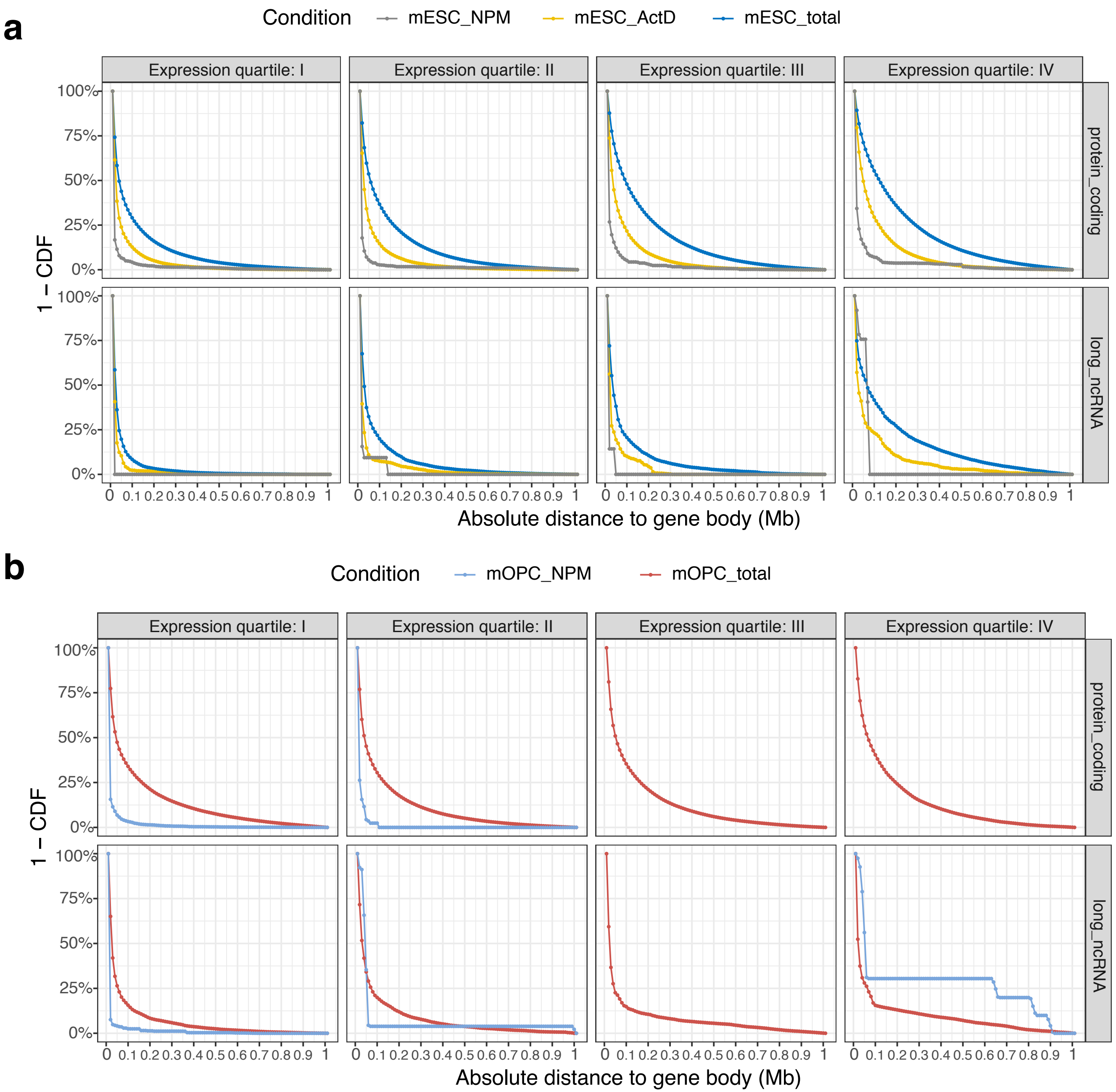

Supplementary Fig. 12. Interaction distances and expression in mESCs and mOPCs. a) Counter cumulative distribution function (1 - CDF) of the absolute (*i.e.*, up- or downstream) linear genomic distances between RNA tags mapping to a gene locus and DNA tags mapping up to 1 Mb away from the same gene locus for NPM (grey), ActD-treated (yellow), and total (blue) mESC datasets. b) Same but for NPM (light blue) and total (red) mOPCs datasets. In each panel, the top row comprises the interactions mediated by protein-coding RNAs, while the bottom row shows those mediated by long non-coding RNAs. The transcripts were divided into four quartile groups based on their CAGE normalized (TPM) counts. All panels were generated using significant datasets.

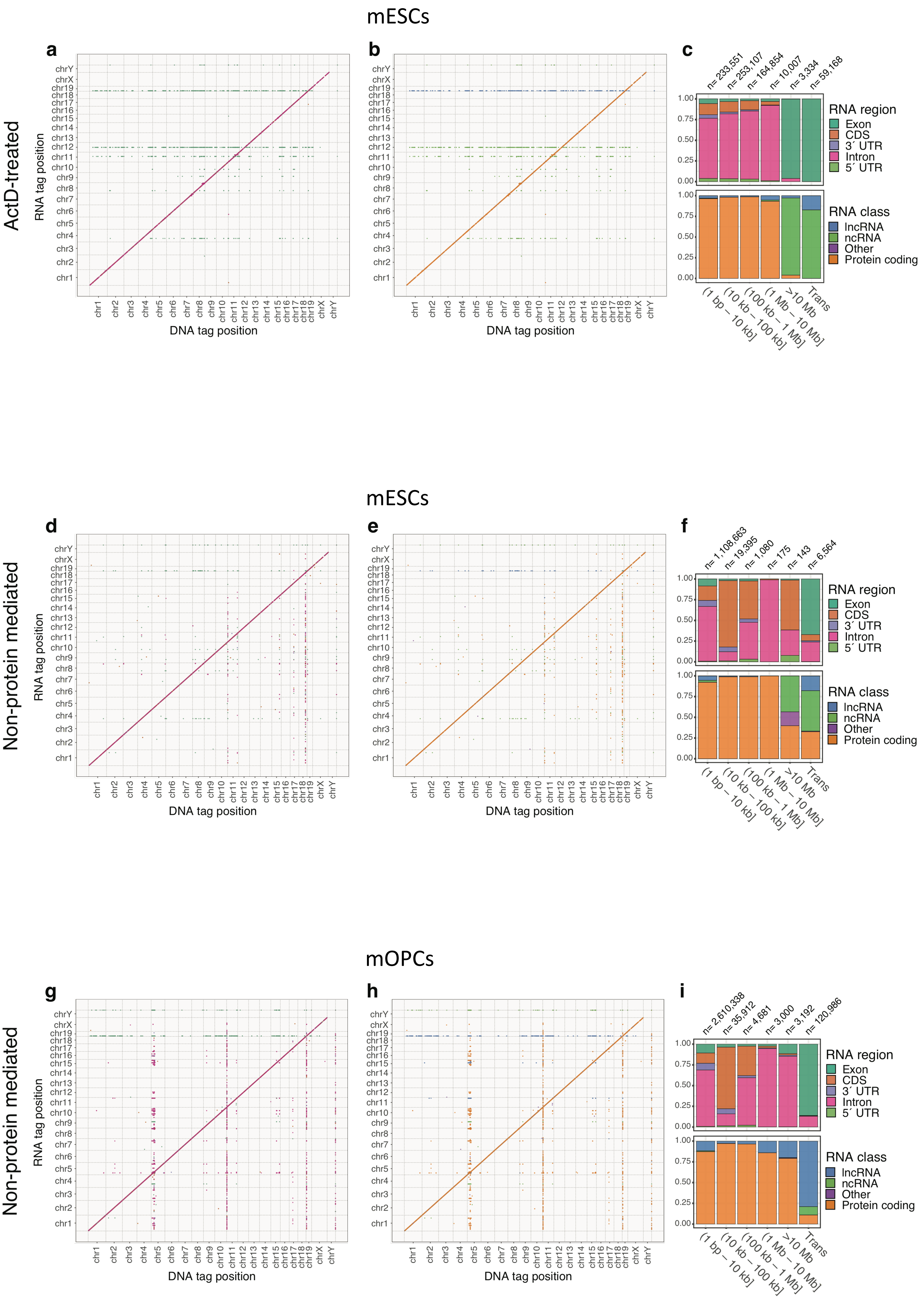

Supplementary Fig. 13. Genome-wide RNA-chromatin features of RADICL-seq libraries from ActD and NPM datasets in mESCs and mOPCs. a,b) RNA-DNA interactions shown as a single point per 25-kb bin and colored (see key in c) by the most represented (a) RNA region or (b) RNA class in that bin for the ActD-treated mESC dataset. c) RNA-DNA interactions in the ActD-treated mESC dataset, quantified according to genomic distance between RNA and DNA tags. d,e) RNA-DNA interaction matrix for the non-protein–mediated (NPM) mESC dataset, similar to a,b. f) RNA-DNA interactions in the NPM mESC dataset, quantified according to genomic distance between RNA and DNA tags. g,h) RNA-DNA interaction matrices for the NPM mOPC dataset, similar to a,b. i) RNA-DNA interactions in the NPM mOPC dataset, quantified according to genomic distance between RNA and DNA tags. All panels were generated using significant datasets.

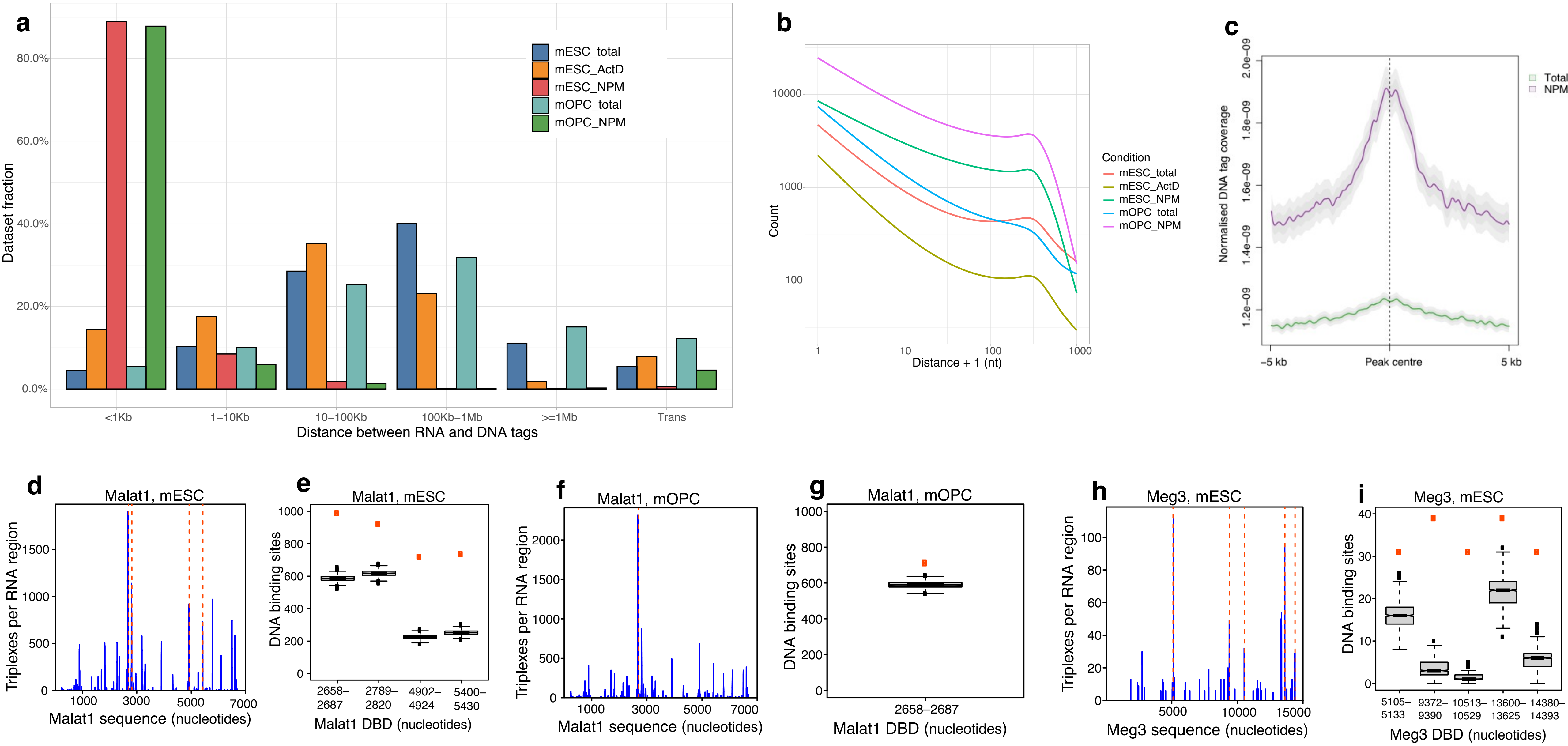

Supplementary Fig. 14. Characteristics of RADICL-seq libraries from NPM datasets. a) Distribution of the linear genomic distances between RNA and DNA tags derived from the same read for the indicated datasets in mESCs and mOPCs. b) Density plot of the linear genomic distance between RNA and DNA for tags located at less than 1 kb from each other in the above datasets. c) Metadata profiles showing the average coverage of RADICL-seq DNA tags at the peaks defined by DRIP-seq signal in mESCs for total and NPM conditions. d, f, h) Number of triplexes formed by various regions of (d, f) Malat1 transcripts and (h) Meg3 transcripts in *trans* contacts in NPM datasets of the indicated cell types. Orange dashed lines correspond to significant DNA binding domains (DBD). e, g, i) Number of the DNA binding sites involved in triplex formation in DNA contacts (orange dots) and in the random background DNA set (boxplots, 1000 permutations). Only DBDs with a significant overrepresentation of triplexes over the background are shown. Statistical significance was calculated using permutation test. All panels were generated using significant datasets.

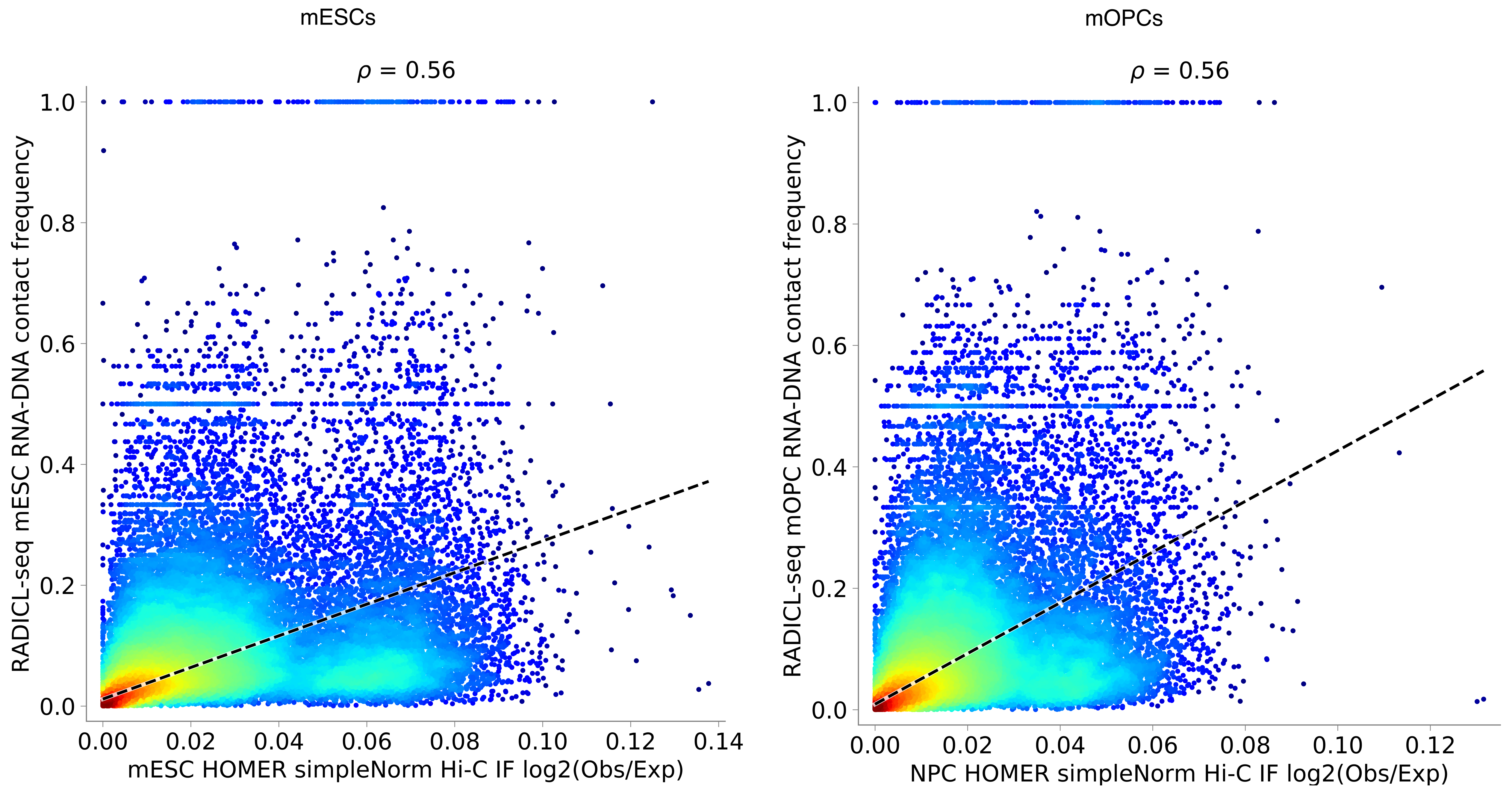

Supplementary Fig. 15. Comparison between RADICL-seq and Hi-C data in mESCs and mOPCs. Pearson correlation (r) of contact frequencies in the HOMER 'simpleNorm' normalized Hi-C vs. significant RADICL-seq total datasets for mESCs (left) and mOPCs (right) at 25-kb resolution.

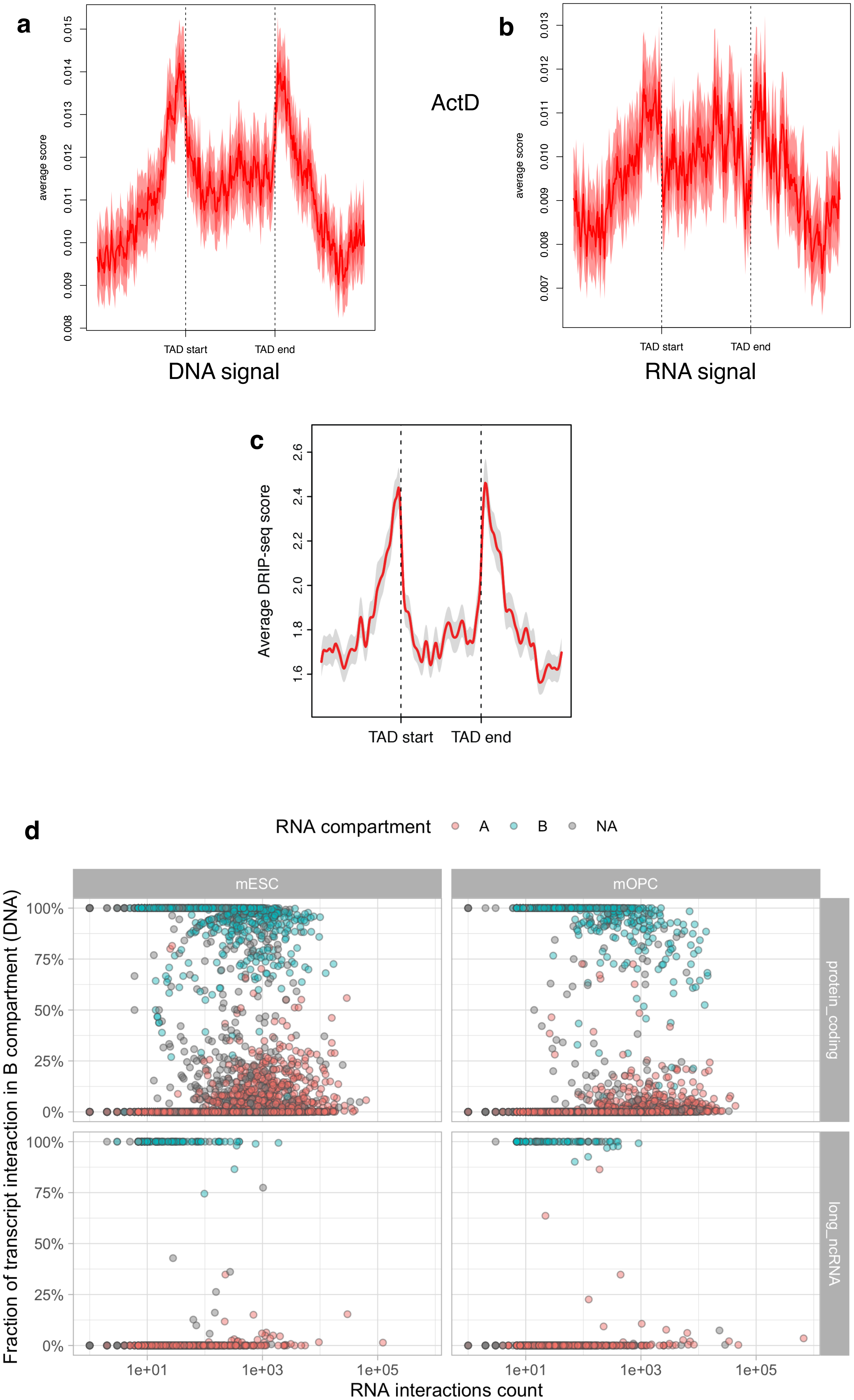

Supplementary Fig. 16. Frequency of RNA-DNA interactions at TADs, A and B compartments in mESC and mOPC datasets. a,b) Metadata profiles showing the average (a) DNA tag or (b) RNA tag coverage at TAD boundaries in mESC datasets for the ActD-treated condition. c) Average DRIP-seq signal at TAD boundaries in mESCs. d) Distribution of interactions between mRNAs (protein_coding) and lncRNAs (long_ncRNA) and A and B compartments in mESC and mOPC total datasets. The color of the points indicates from which compartment the RNA originates (A, red; B, blue; Undetermined, grey). All panels were generated using significant datasets.

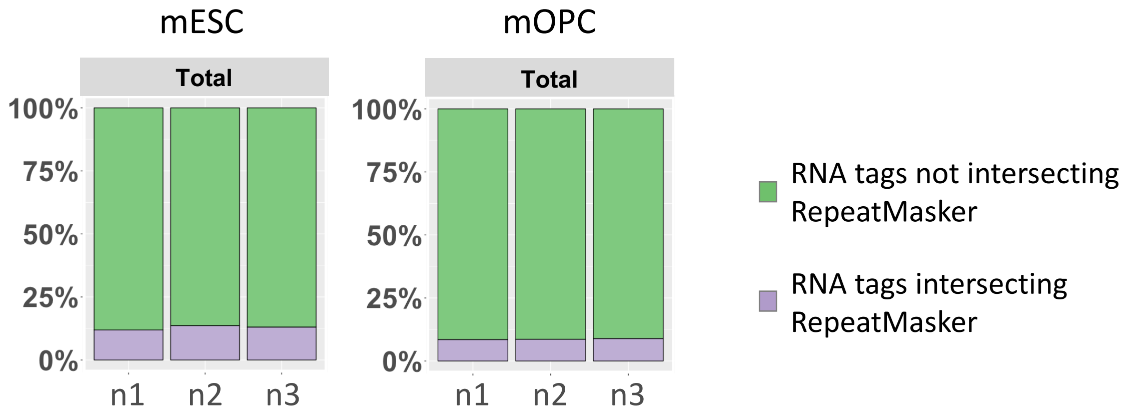

Condition	Significant interactions ($P < 0.05$)	RNA tags not intersecting RepeatMasker	RNA tags intersecting RepeatMasker
mESC_total_n1	2,841,875	2,502,970	338,905
mESC_total_n2	2,608,899	2,252,602	356,297
mESC_total_n3	3,014,022	2,619,960	394,062
mOPC_total_n1	2,378,935	2,176,159	202,776
mOPC_total_n2	2,261,016	2,066,741	194,275
mOPC_total_n3	2,141,631	1,950,400	191,231

Supplementary Fig. 17. Frequency of REs in RADICL-seq libraries from mESCs and mOPCs. Proportion of significant RNA-DNA interactions in total mESC and mOPC libraries with RNA tags intersecting or non-intersecting RepeatMasker elements. The table displays the number of interactions shown in the plot.

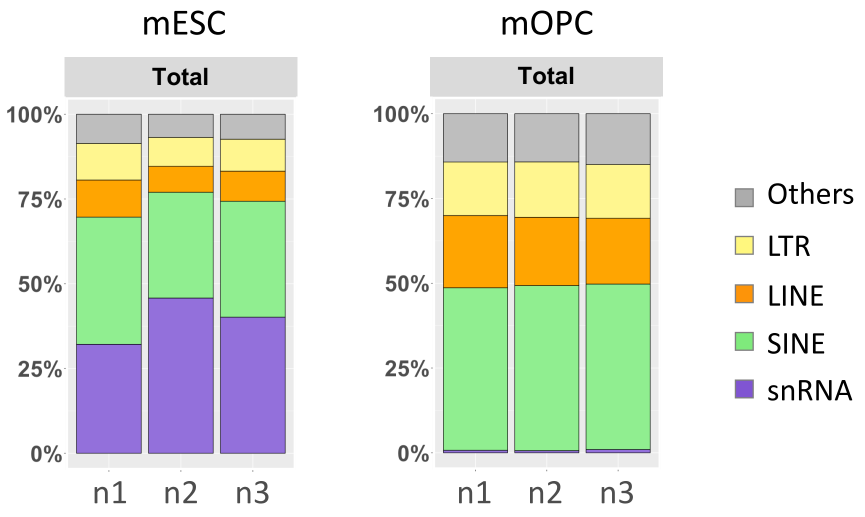

Breakdown of Repeat Families for RNA-DNA pairs with RNA intersecting RepeatMasker elements annotated within GENCODE vM14						
**Condition**	**Total**	**snRNA**	**SINE**	**LINE**	**LTR**	**Others**
mESC_total_n1	264,627	85,071	99,254	28,945	28,542	22,815
mESC_total_n2	285,197	130,571	88,986	21,821	24,298	19,521
mESC_total_n3	309,959	124,454	105,955	27,486	29,215	22,849
mOPC_total_n1	125,633	922	60,210	26,781	19,829	17,891
mOPC_total_n2	128,329	811	62,491	25,828	20,965	18,234
mOPC_total_n3	123,087	1,202	60,047	23,880	19,535	18,423

Supplementary Fig. 18. Distribution of RE families in RADICL-seq libraries from mESCs and mOPCs. Representation of main families of repeats for RNA-DNA pairs where the RNA intersects with RepeatMasker elements contained within GENCODE vM14 annotations for significant total mESC and mOPC libraries. Only non-self interactions were used for this plot. The table displays the count of the interactions used for the plot.

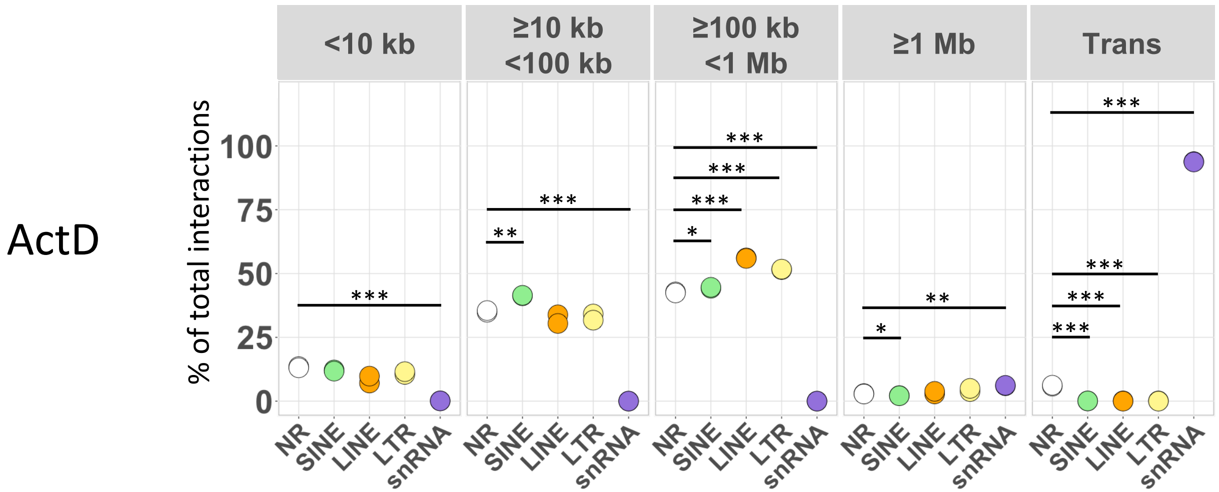

Supplementary Fig. 19. Patterns of RNA-DNA distance intervals for RE-mediated interactions in the ActD condition. Distribution of RNA-DNA distance intervals for RNA tags intersecting RepeatMasker annotations paired with DNA tags intersecting GENCODE annotations in the significant RADICL-seq mESC libraries upon ActD treatment. Only RNA tags intersecting RepeatMasker elements contained within GENCODE ver. M14 features were used for this analysis. Statistical significance was calculated with two-tailed Student *t*-test. * $P \leq 0.05$; ** $P \leq 0.01$; *** $P \leq 0.001$.

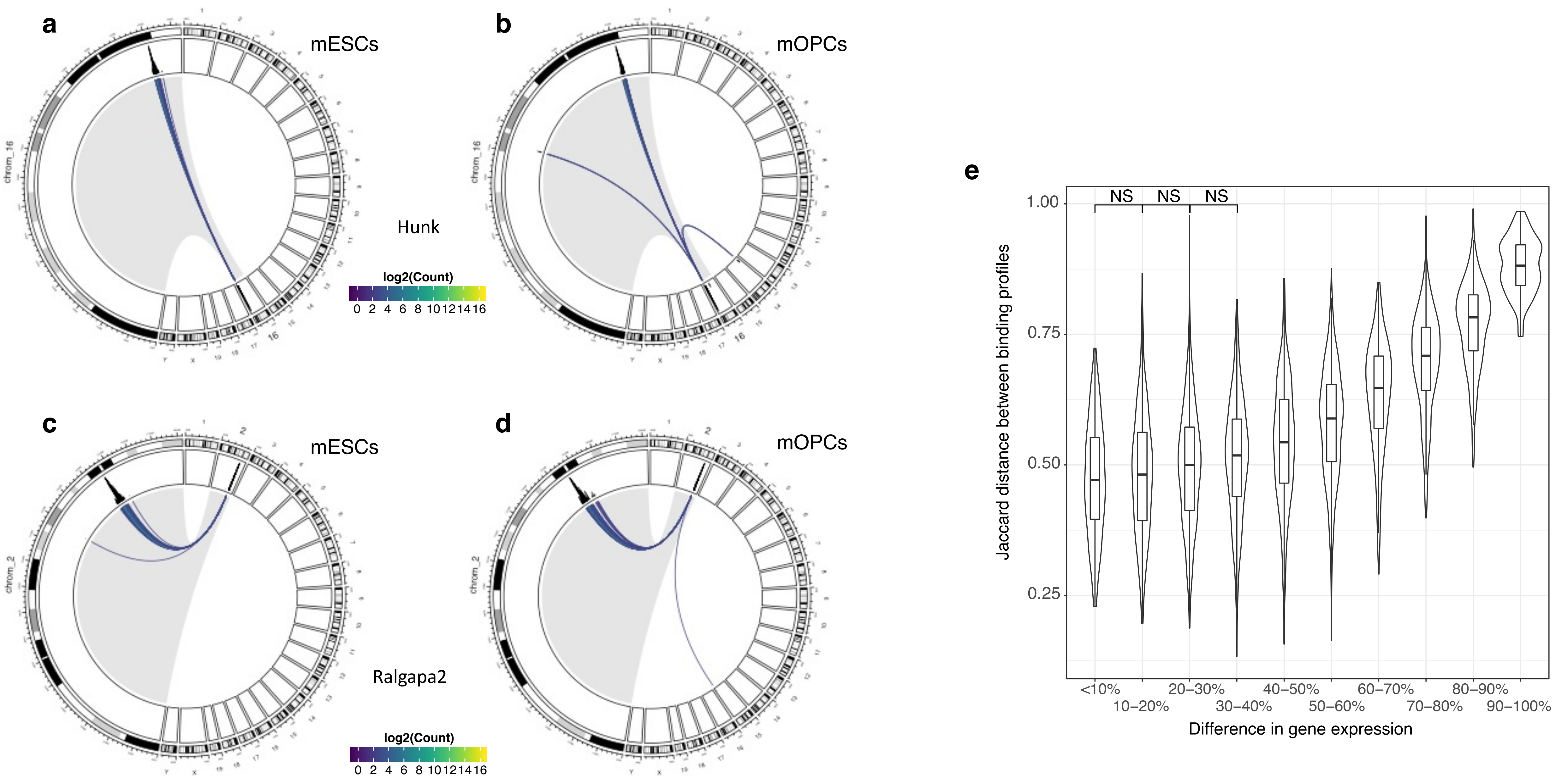

Supplementary Fig. 20. Cell type–specific patterns of interactions. a,b) Circos plots depicting Hunk genomic interactions in mESCs and mOPCs, respectively. c,d) Circos plots depicting Ralgapa2 genomic interactions in mESCs and mOPCs, respectively. Each line represents the interaction between the genic transcript and the contacted genomic bin, while its color indicates log2 of the RADICL-seq count. The chromosome of origin of the RNA under investigation is shown enlarged (gray shading) on the left portion of each circos plot. e) The distribution of Jaccard distances for genome-wide RNA-DNA binding profiles between mESCs and mOPCs for deciles of difference in gene expression between the cell types. For genes with a difference in gene expression between cell types of <40%, there was no significant difference (NS, two-sided Wilcoxon-Mann-Whitney test. $P<0.05$) in the distribution of Jaccard distances for genome-wide RNA-DNA binding profiles. All panels were generated using significant total datasets.

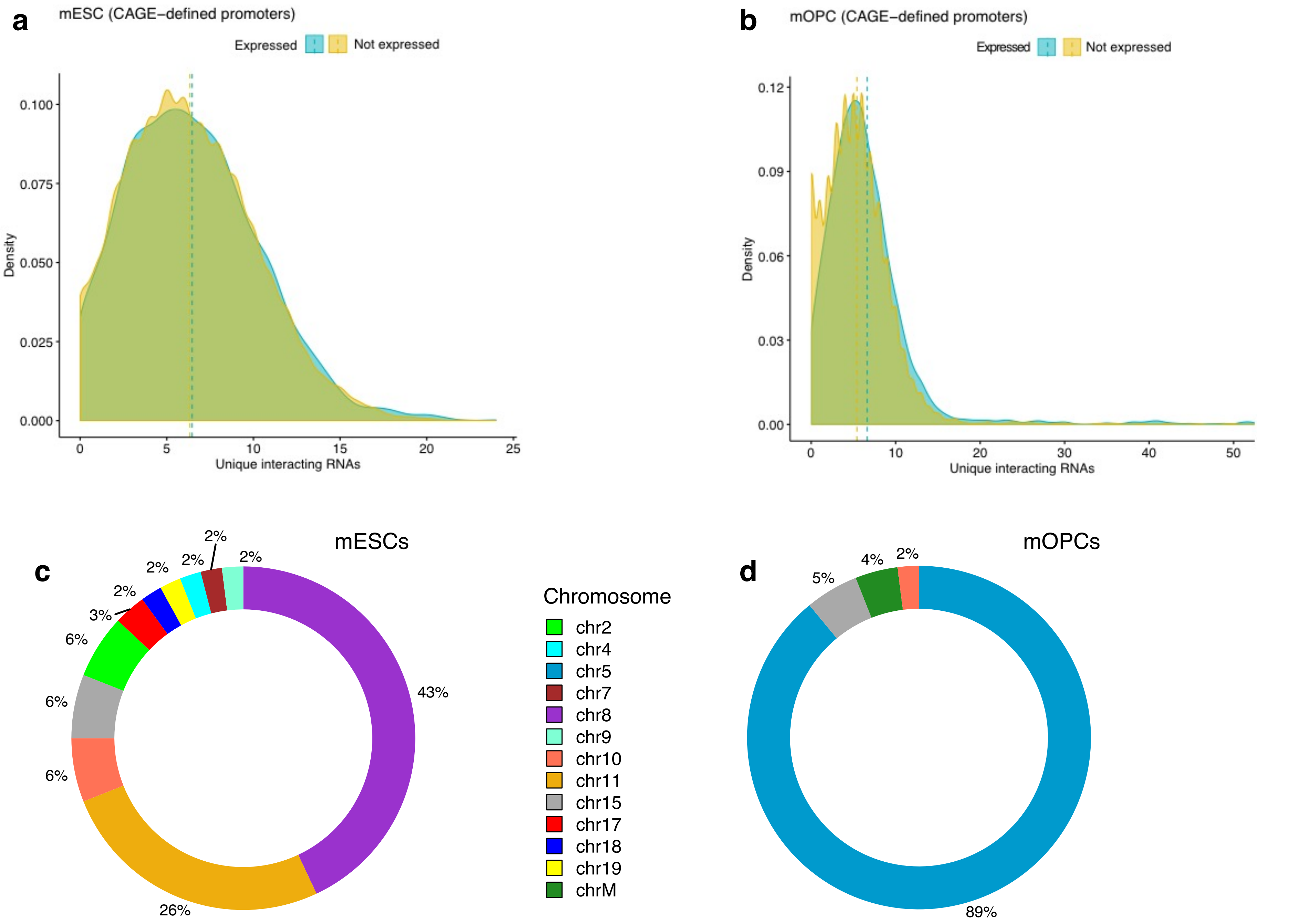

Supplementary Fig. 21. RNA-DNA interactions at promoter regions. a,b) Distribution of unique interacting RNAs on expressed (≥3 TPM; yellow) and not expressed (<3 TPM; blue) CAGE-derived promoters (±2 kb) in mESCs and mOPCs, respectively. c,d) Distribution of CAGE-derived promoters (top 100 ranked by number of unique interacting RNAs) per chromosome in mESCs and mOPCs, respectively. All panels were generated using significant total datasets.

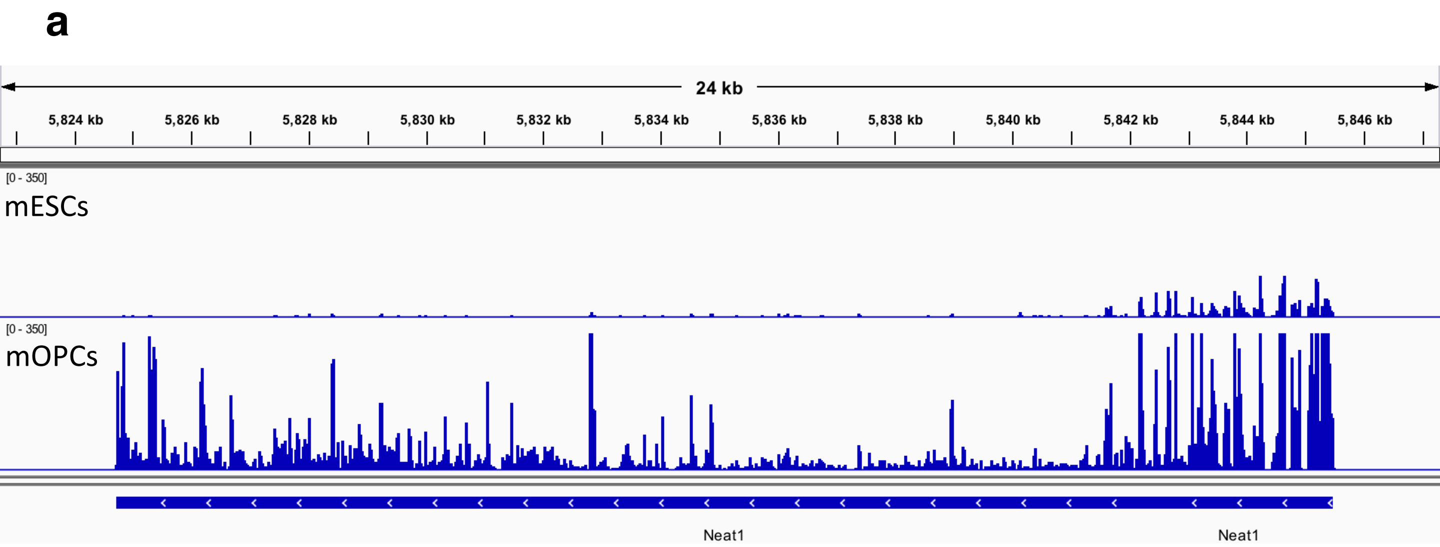

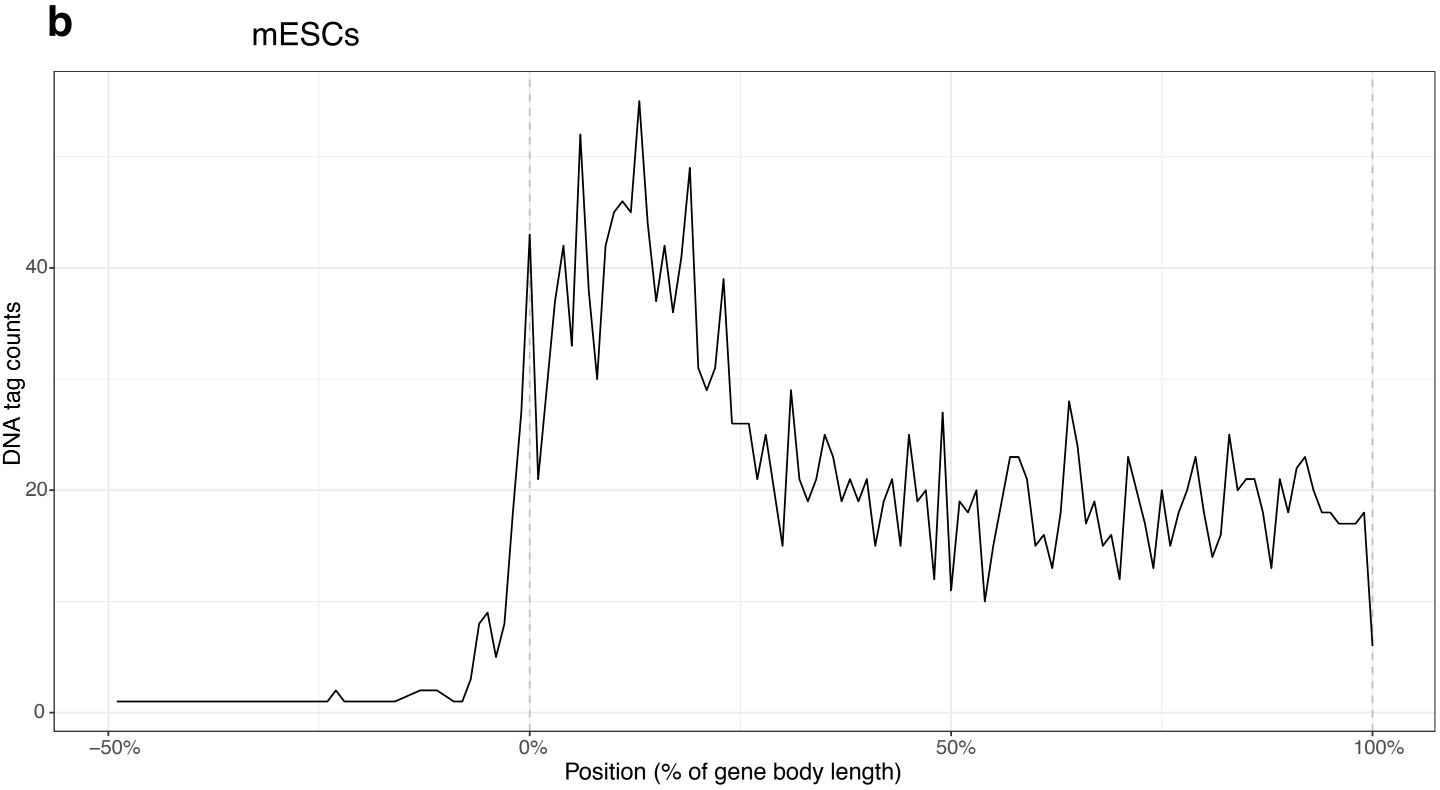

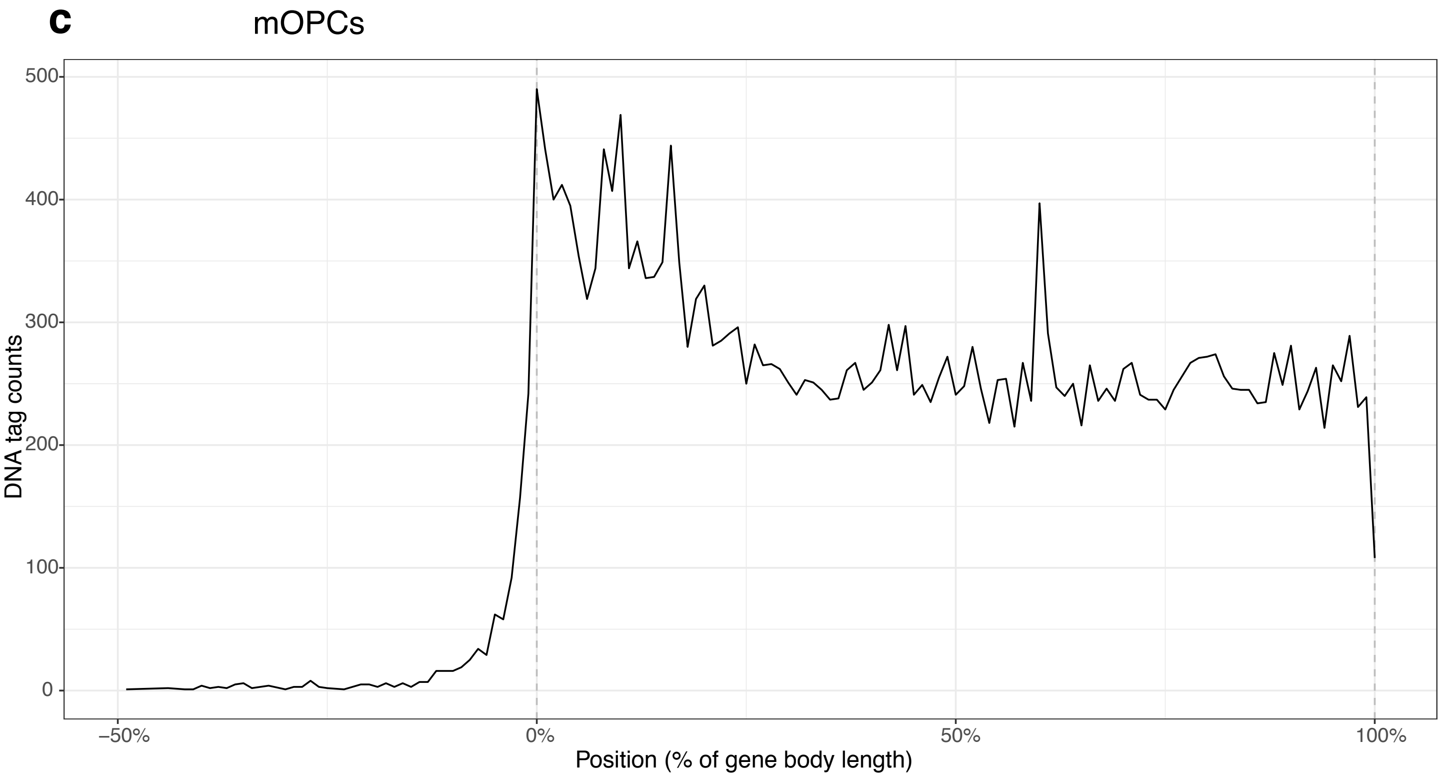

Supplementary Fig. 22. Characterization of Neat1-mediated RNA-chromatin interactions. a) Genomic coverage of Neat1 RNA tags across its gene body in the total mESC dataset (top) and mOPC dataset (bottom). b,c) Density of the DNA tag of Neat1 target genes across their gene bodies in mESCs and mOPCs, respectively. Position is reported as percentage of the gene body length, where 0% and 100% indicate the transcription start and end sites, respectively. All panels were generated using significant datasets.

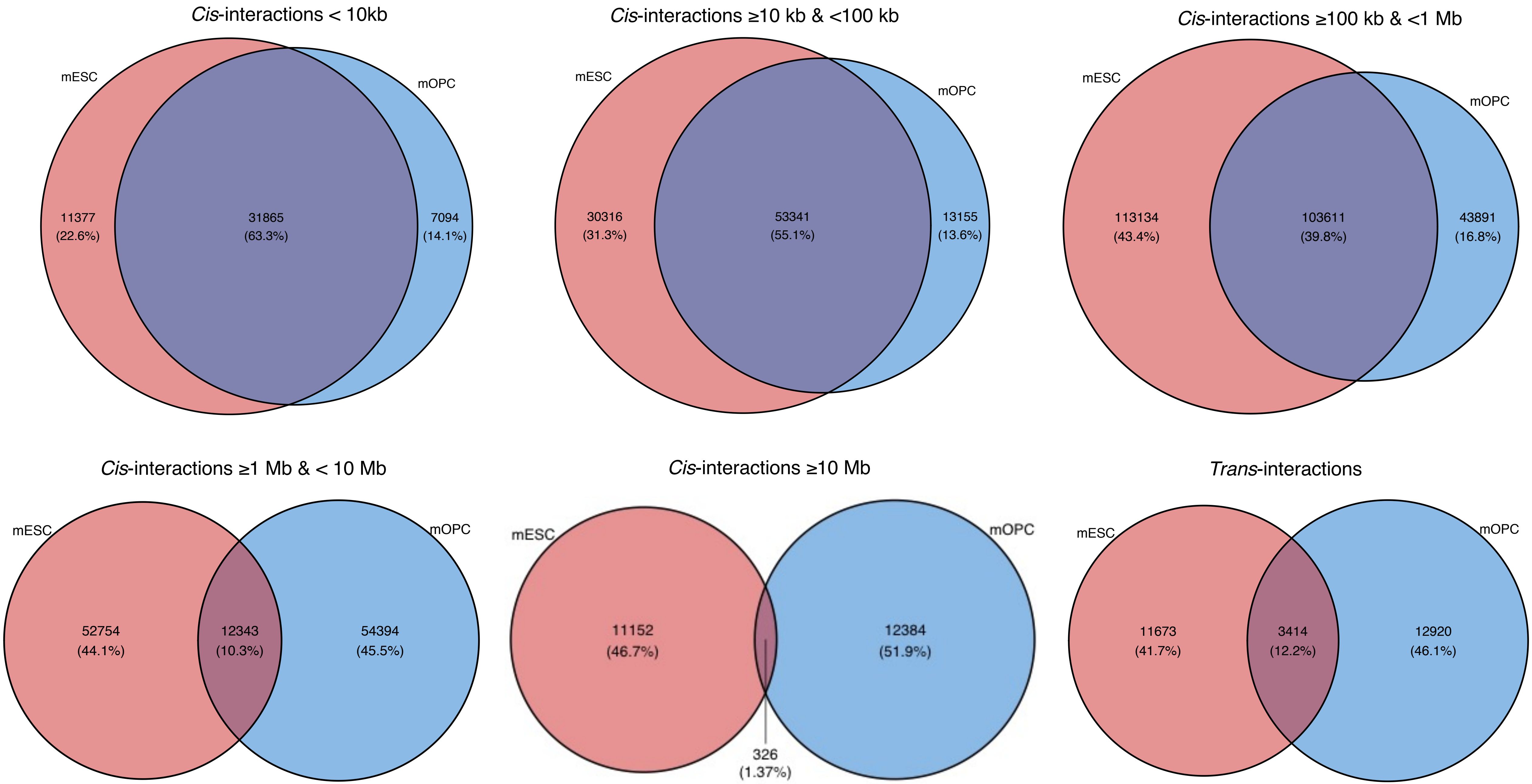

Supplementary Fig. 23. Comparison of *cis*- and *trans*-interactions in mESCs and mOPCs. Interaction overlaps between mESCs (red) and mOPCs (blue) at the indicated linear distances between RNA and DNA tags or interacting in *trans*. All panels were generated using significant total datasets.

Supplementary Table 1. Comparison for capture rates of different RNA biotypes between RADICL-seq and GRID-seq.

BIOTYPE	DETECTED IN BOTH TECHNOLOGIES	DETECTED ONLY WITH GRID-seq	DETECTED ONLY WITH RADICL-seq
antisense	1,614	55	512
lincRNA	2,520	77	918
protein coding	17,322	120	1,937
snoRNA	291	20	28
snRNA	70	42	48

Supplementary Table 2. Top 10 chromatin-interacting RNAs sorted by the number of significant counts in the indicated datasets.

Experimental condition	Transcript ID	Transcript name	biotype	Raw counts	Significant counts
Total	ENSMUSG00000065232.1	Gm22973	ncRNA	918,723	298,499
Total	ENSMUSG00000092341.2	Malat1	Long ncRNA	755,513	145,579
Total	ENSMUSG00000030849.18	Fgfr2	Protein-coding	166,917	69,594
Total	ENSMUSG00000004637.15	Wwox	Protein-coding	134,461	52,644
Total	ENSMUSG00000038518.15	Jarid2	Protein-coding	121,111	43,753
Total	ENSMUSG00000092329.1	Gm20388	Protein-coding	124,753	41,348
Total	ENSMUSG00000069769.13	Msi2	Protein-coding	121,308	40,729
Total	ENSMUSG00000029763.17	Exoc4	Protein-coding	107,330	35,457
Total	ENSMUSG00000097039.8	Pvt1	Long ncRNA	94,372	32,971
Total	ENSMUSG00000042348.9	Arl15	Protein-coding	81,000	31,556
ActD	ENSMUSG00000065232.1	Gm22973	ncRNA	253,794	30,302
ActD	ENSMUSG00000064899.1	Snord118	ncRNA	141,036	14,722
ActD	ENSMUSG00000092341.2	Malat1	Long ncRNA	209,761	10,260
ActD	ENSMUSG00000092329.1	Gm20388	Protein-coding	27,615	7,160
ActD	ENSMUSG00000030849.18	Fgfr2	Protein-coding	23,037	3,576
ActD	ENSMUSG00000069769.13	Msi2	Protein-coding	20,143	3,454
ActD	ENSMUSG00000029763.17	Exoc4	Protein-coding	21,040	3,021
ActD	ENSMUSG00000035569.17	Ankrd11	Protein-coding	16,565	2,553
ActD	ENSMUSG00000048402.14	Gli2	Protein-coding	12,441	2,534
ActD	ENSMUSG00000097039.8	Pvt1	Long ncRNA	16,153	2,399
NPM	ENSMUSG00000092341.2	Malat1	Long ncRNA	252,334	18,946
NPM	ENSMUSG00000064337.1	mt-Rnr1	ncRNA	146,847	5,325
NPM	ENSMUSG00000064339.1	mt-Rnr2	ncRNA	113,093	3,927
NPM	ENSMUSG00000088088.1	Rmrp	ncRNA	169,160	3,753
NPM	ENSMUSG00000030849.18	Fgfr2	Protein-coding	54,785	3,536
NPM	ENSMUSG00000004637.15	Wwox	Protein-coding	50,436	3,253
NPM	ENSMUSG00000092329.1	Gm20388	Protein-coding	41,456	3,204
NPM	ENSMUSG00000038518.15	Jarid2	Protein-coding	44,665	2,786
NPM	ENSMUSG00000029763.17	Exoc4	Protein-coding	43,909	2,641
NPM	ENSMUSG00000069769.13	Msi2	Protein-coding	38,209	2,587

Supplementary Table 3. Analysis of lncRNA distance interaction patterns. For each range of RNA and DNA distance intervals, the numbers of unique lncRNAs and the numbers and percentages of all lncRNA-mediated interactions are reported for both mESCs and mOPCs significant total datasets.

Condition	Distance interval	Unique lncRNAs	Interactions	Percentage
mESCs total	≤10 kb	1,422	40,952	12.8%
mESCs total	>10 kb & ≤100 kb	1,177	52,280	16.4%
mESCs total	>100 kb & ≤1 Mb	285	60,331	18.9%
mESCs total	>1 Mb & ≤10 Mb	40	23,090	7.2%
mESCs total	>10 Mb	5	9,267	2.9%
mESCs total	Trans	3	133,705	41.8%
mOPCs total	≤10 kb	1074	58,781	5.4%
mOPCs total	>10 kb & ≤100 kb	958	68,932	6.4%
mOPCs total	>100 kb & ≤1 Mb	250	91,052	8.5%
mOPCs total	>1 Mb & ≤10 Mb	62	66,539	6.1%
mOPCs total	>10 Mb	17	38,277	3.5%
mOPCs total	Trans	10	759,844	70.1%

Supplementary Table 4. Top ten interacting transcripts for *cis*- and *trans*-contacts in each experimental condition. Only lncRNAs and protein-coding (pc) RNAs are listed for each condition. Data produced from significant datasets.

Condition	Gene ID	Gene Name	Number of Contacts
mESC_total	ENSMUSG00000092341.2	Malat1	134436
mESC_total	ENSMUSG00000106943.1	Dancr	104
mESC_total	ENSMUSG00000092274.2	Neat1	57
mESC_total	ENSMUSG00000097451.10	Rian	15
mESC_total	ENSMUSG00000021268.17	Meg3	12
mESC_total	ENSMUSG00000101609.1	Kcnq1ot1	12
mESC_total	ENSMUSG00000108414.1	Snhg1	9
mESC_total	ENSMUSG00000078952.9	Lncenc1	7
mESC_total	ENSMUSG00000085396.7	Firre	6
mESC_total	ENSMUSG00000097391.8	Mirg	3
mESC_ActD	ENSMUSG00000092341.2	Malat1	9277
mESC_ActD	ENSMUSG00000106943.1	Dancr	9
mESC_ActD	ENSMUSG00000097451.10	Rian	5
mESC_NPM	ENSMUSG00000092341.2	Malat1	2206
mESC_NPM	ENSMUSG00000092274.2	Neat1	58
mESC_NPM	ENSMUSG00000021268.17	Meg3	41
mESC_NPM	ENSMUSG00000101609.1	Kcnq1ot1	38
mESC_NPM	ENSMUSG00000097451.10	Rian	13
mESC_NPM	ENSMUSG00000078952.9	Lncenc1	13
mESC_NPM	ENSMUSG00000085438.1	1700020I14Rik	8
mESC_NPM	ENSMUSG00000097391.8	Mirg	6
mESC_NPM	ENSMUSG00000085715.2	Tsix	5
mESC_NPM	ENSMUSG00000085396.7	Firre	5
mOPC_total	ENSMUSG00000092341.2	Malat1	735180
mOPC_total	ENSMUSG00000092274.2	Neat1	24374
mOPC_total	ENSMUSG00000106943.1	Dancr	173
mOPC_total	ENSMUSG00000073147.3	5031425E22Rik	16
mOPC_total	ENSMUSG00000087259.7	2610035D17Rik	13
mOPC_total	ENSMUSG00000101609.1	Kcnq1ot1	12
mOPC_total	ENSMUSG00000097039.8	Pvt1	12
mOPC_total	ENSMUSG00000097536.2	2610037D02Rik	8
mOPC_total	ENSMUSG00000097814.5	Panct2	2
mOPC_total	ENSMUSG00000075555.11	Gm10863	1
mOPC_NPM	ENSMUSG00000092341.2	Malat1	76184
mOPC_NPM	ENSMUSG00000092274.2	Neat1	12781
mOPC_NPM	ENSMUSG00000101609.1	Kcnq1ot1	47
mOPC_NPM	ENSMUSG00000097039.8	Pvt1	36
mOPC_NPM	ENSMUSG00000073147.3	5031425E22Rik	24
mOPC_NPM	ENSMUSG00000087259.7	2610035D17Rik	22
mOPC_NPM	ENSMUSG00000098243.3	Gm4258	4
mOPC_NPM	ENSMUSG00000097207.7	6030443J06Rik	3
mOPC_NPM	ENSMUSG00000105265.4	Sox2ot	3
mOPC_NPM	ENSMUSG00000054556.6	Gm4876	2

pcRNA trans-interactions

Condition	Gene ID	Gene Name	Number of Contacts
mESC_total	ENSMUSG00000062647.16	Rpl7a	18
mESC_total	ENSMUSG00000027404.15	Snrpb	14
mESC_total	ENSMUSG00000036427.5	Gpi1	9
mESC_total	ENSMUSG00000004980.16	Hnrnpa2b1	5
mESC_total	ENSMUSG00000031939.16	Taf1d	5
mESC_total	ENSMUSG00000078578.9	Ube2d3	5
mESC_total	ENSMUSG00000000131.15	Xpo6	4
mESC_total	ENSMUSG00000021767.16	Kat6b	4
mESC_total	ENSMUSG00000022884.14	Eif4a2	4
mESC_total	ENSMUSG00000030275.6	Etnk1	4
mESC_NPM	ENSMUSG00000010608.15	Rbm25	14
mESC_NPM	ENSMUSG00000021196.14	Pfkp	14
mESC_NPM	ENSMUSG00000025964.15	Adam23	12
mESC_NPM	ENSMUSG00000021767.16	Kat6b	11
mESC_NPM	ENSMUSG00000022884.14	Eif4a2	10
mESC_NPM	ENSMUSG00000030275.6	Etnk1	9
mESC_NPM	ENSMUSG00000035569.17	Ankrd11	9
mESC_NPM	ENSMUSG00000004980.16	Hnrnpa2b1	8
mESC_NPM	ENSMUSG00000029817.11	Tra2a	8
mESC_NPM	ENSMUSG00000032582.14	Rbm6	8
mOPC_total	ENSMUSG00000026872.17	Zeb2	22
mOPC_total	ENSMUSG00000023951.17	Vegfa	13
mOPC_total	ENSMUSG00000027404.15	Snrpb	11
mOPC_total	ENSMUSG00000031939.16	Taf1d	11
mOPC_total	ENSMUSG00000051910.13	Sox6	11
mOPC_total	ENSMUSG00000062647.16	Rpl7a	10
mOPC_total	ENSMUSG00000039630.10	Hnrnpu	6
mOPC_total	ENSMUSG00000068748.7	Ptprz1	6
mOPC_total	ENSMUSG00000005103.12	Wdr1	5
mOPC_total	ENSMUSG00000029673.17	Auts2	4
mOPC_NPM	ENSMUSG00000051910.13	Sox6	15
mOPC_NPM	ENSMUSG00000026872.17	Zeb2	9
mOPC_NPM	ENSMUSG00000005103.12	Wdr1	8
mOPC_NPM	ENSMUSG00000056073.16	Grik2	7
mOPC_NPM	ENSMUSG00000053477.16	Tcf4	7
mOPC_NPM	ENSMUSG00000004642.13	Slbp	6
mOPC_NPM	ENSMUSG00000026615.14	Eprs	5
mOPC_NPM	ENSMUSG00000059921.15	Unc5c	5
mOPC_NPM	ENSMUSG00000068748.7	Ptprz1	4
mOPC_NPM	ENSMUSG00000004980.16	Hnrnpa2b1	3

lncRNA cis-interactions

Condition	Gene ID	Gene Name	Number of Contacts
mESC_total	ENSMUSG00000101609.1	Kcnq1ot1	9410
mESC_total	ENSMUSG00000092341.2	Malat1	9363
mESC_total	ENSMUSG00000092274.2	Neat1	2186
mESC_total	ENSMUSG00000021268.17	Meg3	1628
mESC_total	ENSMUSG00000078952.9	Lncenc1	812
mESC_total	ENSMUSG00000085385.7	Snhg17	536
mESC_total	ENSMUSG00000110282.1	B930086L07Rik	536
mESC_total	ENSMUSG00000097451.10	Rian	515
mESC_total	ENSMUSG00000097039.8	Pvt1	483
mESC_total	ENSMUSG00000085715.2	Tsix	474
mESC_ActD	ENSMUSG00000092341.2	Malat1	489
mESC_ActD	ENSMUSG00000101609.1	Kcnq1ot1	171
mESC_ActD	ENSMUSG00000096751.3	Gm28373	86
mESC_ActD	ENSMUSG00000021268.17	Meg3	84
mESC_ActD	ENSMUSG00000110558.1	Gm45779	52
mESC_ActD	ENSMUSG00000078952.9	Lncenc1	39
mESC_ActD	ENSMUSG00000097000.2	Gm17435	38
mESC_ActD	ENSMUSG00000092274.2	Neat1	31
mESC_ActD	ENSMUSG00000100691.1	2010320M18Rik	22
mESC_ActD	ENSMUSG00000097039.8	Pvt1	20
mESC_NPM	ENSMUSG00000092203.7	1110038B12Rik	20
mESC_NPM	ENSMUSG00000105260.1	Gm42434	16
mESC_NPM	ENSMUSG00000092341.2	Malat1	9
mESC_NPM	ENSMUSG00000110282.1	B930086L07Rik	8
mESC_NPM	ENSMUSG00000111394.1	AC160637.1	6
mESC_NPM	ENSMUSG00000079489.2	C030013D06Rik	5
mESC_NPM	ENSMUSG00000102657.1	Gm37899	5
mESC_NPM	ENSMUSG00000110993.1	Gm47963	5
mESC_NPM	ENSMUSG00000070461.3	9230112E08Rik	4
mESC_NPM	ENSMUSG00000102411.1	Gm36936	4
mOPC_total	ENSMUSG00000092341.2	Malat1	26738
mOPC_total	ENSMUSG00000092274.2	Neat1	9033
mOPC_total	ENSMUSG00000101609.1	Kcnq1ot1	8420
mOPC_total	ENSMUSG00000073147.3	5031425E22Rik	2155
mOPC_total	ENSMUSG00000097039.8	Pvt1	936
mOPC_total	ENSMUSG00000107197.1	Gm43312	738
mOPC_total	ENSMUSG00000107083.1	Gm43313	664
mOPC_total	ENSMUSG00000070461.3	9230112E08Rik	532
mOPC_total	ENSMUSG00000097536.2	2610037D02Rik	441
mOPC_total	ENSMUSG00000104118.1	Gm37298	310
mOPC_NPM	ENSMUSG00000092341.2	Malat1	552
mOPC_NPM	ENSMUSG00000092274.2	Neat1	174
mOPC_NPM	ENSMUSG00000073147.3	5031425E22Rik	45
mOPC_NPM	ENSMUSG00000094832.1	Gm21846	26
mOPC_NPM	ENSMUSG00000103038.1	Gm37124	22
mOPC_NPM	ENSMUSG00000103427.1	Gm37534	20
mOPC_NPM	ENSMUSG00000070461.3	BX649560.1	16
mOPC_NPM	ENSMUSG00000107197.1	Gm43312	16
mOPC_NPM	ENSMUSG00000085334.7	Gm12940	14
mOPC_NPM	ENSMUSG00000111212.1	CAAA01194877.1	13

pcRNA cis-interactions

Condition	Gene ID	Gene Name	Number of Contacts
mESC_total	ENSMUSG00000021767.16	Kat6b	863
mESC_total	ENSMUSG00000035569.17	Ankrd11	815
mESC_total	ENSMUSG00000078578.9	Ube2d3	814
mESC_total	ENSMUSG00000025964.15	Adam23	799
mESC_total	ENSMUSG00000022995.16	Enah	724
mESC_total	ENSMUSG00000031575.18	Ash2l	645
mESC_total	ENSMUSG00000038518.15	Jarid2	616
mESC_total	ENSMUSG00000055320.17	Tead1	594
mESC_total	ENSMUSG00000029178.14	Klf3	570
mESC_total	ENSMUSG00000040952.16	Rps19	520
mESC_ActD	ENSMUSG00000033732.10	Sf3b3	92
mESC_ActD	ENSMUSG00000029817.11	Tra2a	64
mESC_ActD	ENSMUSG00000035569.17	Ankrd11	55
mESC_ActD	ENSMUSG00000029439.14	Sfswap	50
mESC_ActD	ENSMUSG00000037957.14	Wdr20	49
mESC_ActD	ENSMUSG00000038095.15	Sbno1	49
mESC_ActD	ENSMUSG00000034269.12	Setd5	48
mESC_ActD	ENSMUSG00000024576.14	Csnk1a1	47
mESC_ActD	ENSMUSG00000059796.16	Eif4a1	44
mESC_ActD	ENSMUSG00000040731.13	Eif4h	42
mESC_NPM	ENSMUSG00000071172.12	Srsf3	5
mESC_NPM	ENSMUSG00000023944.14	Hsp90ab1	4
mESC_NPM	ENSMUSG00000024073.14	Birc6	4
mESC_NPM	ENSMUSG00000024163.17	Mapk8ip3	3
mESC_NPM	ENSMUSG00000028461.12	Ccdc107	3
mESC_NPM	ENSMUSG00000036026.15	Tmem63b	3
mESC_NPM	ENSMUSG00000036036.15	Zfp57	3
mESC_NPM	ENSMUSG00000039218.16	Srrm2	3
mESC_NPM	ENSMUSG00000023991.16	Foxp4	2
mESC_NPM	ENSMUSG00000024459.18	H2-M5	2
mOPC_total	ENSMUSG00000026872.17	Zeb2	2262
mOPC_total	ENSMUSG00000005103.12	Wdr1	1592
mOPC_total	ENSMUSG00000014956.15	Ppp1cb	1160
mOPC_total	ENSMUSG00000032525.15	Nktr	722
mOPC_total	ENSMUSG00000037235.13	Mxd4	711
mOPC_total	ENSMUSG00000032911.6	Cspg4	624
mOPC_total	ENSMUSG00000053007.9	Creb5	491
mOPC_total	ENSMUSG00000059921.15	Unc5c	481
mOPC_total	ENSMUSG00000053477.16	Tcf4	469
mOPC_total	ENSMUSG00000053007.9	Creb5	453
mOPC_NPM	ENSMUSG00000029673.17	Auts2	138
mOPC_NPM	ENSMUSG00000029138.4	4930548H24Rik	138
mOPC_NPM	ENSMUSG00000079555.2	Haus3	144
mOPC_NPM	ENSMUSG00000037605.16	Adgrl3	141
mOPC_NPM	ENSMUSG00000052139.18	Bre	176
mOPC_NPM	ENSMUSG00000015452.14	Ager	407
mOPC_NPM	ENSMUSG00000020882.17	Cacnb1	96
mOPC_NPM	ENSMUSG00000025138.14	Sirt7	180
mOPC_NPM	ENSMUSG00000028035.13	Dnajb4	180
mOPC_NPM	ENSMUSG00000033102.15	Cdc14b	264

Supplementary Table 5. List of cell type-specific markers.

mESC markers, list modified from Zhao, W. *et al. Molecules* **17**, 6196-6246 (2012).

Gene name	Gene ID
Cd324	ENSMUSG00000000303.12
Nacc1	ENSMUSG00000001910.4
Klf4	ENSMUSG00000003032.8
Stat3	ENSMUSG00000004040.16
Cd117	ENSMUSG00000005672.12
Nanog	ENSMUSG00000012396.12
Tbx3	ENSMUSG00000018604.18
Essrb	ENSMUSG00000021255.17
Pou5f1	ENSMUSG00000024406.16
Tfcp2l1	ENSMUSG00000026380.10
Sall4	ENSMUSG00000027547.17
Lef1	ENSMUSG00000027985.14
Cd90	ENSMUSG00000032011.5
Cripto	ENSMUSG00000032494.12
Fbxo15	ENSMUSG00000034391.10
Gbx2	ENSMUSG00000034486.8
Cd326	ENSMUSG00000045394.8
Dppa3	ENSMUSG00000046323.8
Utf1	ENSMUSG00000047751.9
Ssea-1	ENSMUSG00000049307.6
Rex1	ENSMUSG00000051176.6
Klf2	ENSMUSG00000055148.7
Hmga2	ENSMUSG00000056758.14
Dppa4	ENSMUSG00000058550.14
Dppa5	ENSMUSG00000060461.5
Gcnf	ENSMUSG00000063972.13
Foxd3	ENSMUSG00000067261.4
Dppa2	ENSMUSG00000072419.4
Sox2	ENSMUSG00000074637.7
Zfx	ENSMUSG00000079509.10

mOPC markers, list modified from Marques S. *et al*. Science 352, 1326-1329 (2016).

Gene name	Gene ID
Gria3	ENSMUSG00000001986.16
Bcan	ENSMUSG00000004892.13
Slc1a2	ENSMUSG00000005089.15
Atp1a2	ENSMUSG00000007097.14
Tnr	ENSMUSG00000015829.13
Matn4	ENSMUSG00000016995.17
Fabp7	ENSMUSG00000019874.11
Ascl1	ENSMUSG00000020052.9
Id2	ENSMUSG00000020644.9
Zfp36l1	ENSMUSG00000021127.7
Vcan	ENSMUSG00000021614.16
Ednrb	ENSMUSG00000022122.14
Serpine2	ENSMUSG00000026249.10
Gpr37l1	ENSMUSG00000026424.8
Pdgfra	ENSMUSG00000029231.15
Tmem176b	ENSMUSG00000029810.15
Tpm1	ENSMUSG00000032366.15
Cspg5	ENSMUSG00000032482.9
Cspg4	ENSMUSG00000032911.6

Sox10	ENSMUSG00000033006.9
Neu4	ENSMUSG00000034000.15
Rlbp1	ENSMUSG00000039194.15
Olig2	ENSMUSG00000039830.8
Tril	ENSMUSG00000043496.7
S100a1	ENSMUSG00000044080.9
C1ql1	ENSMUSG00000045532.5
Olig1	ENSMUSG00000046160.6
Ncald	ENSMUSG00000051359.14
Sox6	ENSMUSG00000051910.13
Gpr17	ENSMUSG00000052229.5
Pcdh15	ENSMUSG00000052613.16
Sox11	ENSMUSG00000063632.6
Nnat	ENSMUSG00000067786.16
Ptprz1	ENSMUSG00000068748.7
Tmem100	ENSMUSG00000069763.3
Ccnd1	ENSMUSG00000070348.5
Kcnip3	ENSMUSG00000079056.12
Lhfpl3	ENSMUSG00000106379.1

Supplementary Table 6. Shared *trans*-interacting long noncoding RNAs captured in both mESCs and mOPCs. For each transcript, the numbers of shared interactions detected in the significant total dataset for each cell type are shown.

Transcript ID	Name	mESCs total	mOPCs total
ENSMUSG00000092341.2	Malat1	86,770	209,954
ENSMUSG00000064899.1	Snord118	294	103
ENSMUSG00000088088.1	Rmrp	112	106
ENSMUSG00000092274.2	Neat1	15	48
ENSMUSG00000064337.1	Mt-Rnr1	15	36
ENSMUSG00000023795.16	Pisd-ps2	14	23
ENSMUSG00000097039.8	Pvt1	14	18
ENSMUSG00000064339.1	Mt-Rnr2	13	13
ENSMUSG00000065037.1	Rn7sk	12	20
ENSMUSG00000026131.18	Dst	9	11
ENSMUSG00000065878.1	Snord34	8	21
ENSMUSG00000077734.1	Snord83b	8	4
ENSMUSG00000048264.16	Dip2c	7	7
ENSMUSG00000104627.1	Mir3535	4	6
