# Supplementary material for: RADICL-seq identifies general and cell type–specific principles of genome-wide RNA-chromatin interactions
Source: Nat Commun. 2020 Feb 24;11:1018. doi: 10.1038/s41467-020-14337-6 (PMC7039879; doi:10.1038/s41467-020-14337-6)
Supplement: Supplementary file 3 — Reporting Summary [file 41467_2020_14337_MOESM3_ESM.pdf]

nature research

Corresponding author(s): Bonetti Alessandro, Valerio Orlando, Piero Carninci

Last updated by author(s): Dec 12, 2019

Reporting Summary

Nature Research wishes to improve the reproducibility of the work that we publish. This form provides structure for consistency and transparency in reporting. For further information on Nature Research policies, seeAuthors & Referees and theEditorial Policy Checklist .

Statistics

For all statistical analyses, confirm that the following items are present in the figure legend, table legend, main text, or Methods section.

n/a	Confirmed	
☐	☒	The exact sample size (*n*) for each experimental group/condition, given as a discrete number and unit of measurement
☐	☒	A statement on whether measurements were taken from distinct samples or whether the same sample was measured repeatedly
☐	☒	The statistical test(s) used AND whether they are one- or two-sided<br>*Only common tests should be described solely by name; describe more complex techniques in the Methods section.*
☒	☐	A description of all covariates tested
☐	☒	A description of any assumptions or corrections, such as tests of normality and adjustment for multiple comparisons
☒	☐	A full description of the statistical parameters including central tendency (e.g. means) or other basic estimates (e.g. regression coefficient) AND variation (e.g. standard deviation) or associated estimates of uncertainty (e.g. confidence intervals)
☐	☒	For null hypothesis testing, the test statistic (e.g. *F*, *t*, *r*) with confidence intervals, effect sizes, degrees of freedom and *P* value noted<br>*Give P values as exact values whenever suitable.*
☒	☐	For Bayesian analysis, information on the choice of priors and Markov chain Monte Carlo settings
☒	☐	For hierarchical and complex designs, identification of the appropriate level for tests and full reporting of outcomes
☒	☐	Estimates of effect sizes (e.g. Cohen's *d*, Pearson's *r*), indicating how they were calculated

Our web collection on statistics for biologists contains articles on many of the points above.

Software and code

Policy information about availability of computer code

Data collection	*Provide a description of all commercial, open source and custom code used to collect the data in this study, specifying the version used OR state that no software was used.*
Data analysis	TagDust2 (ver. 2.31), RNAdust (ver. 1.0.6), BWA (ver. 0.7.15-r1140) , samtools (ver. 1.3.1), bedtools (ver. 2.17.0), STAR (v2.5.0a), MEME (v4.11.2), MACS (v2.1.1.20160309), TrimGalore! (v0.4.5), Bowtie2 (v2.3.3.1), Picard (v2.9.0.21), HiCUP (v0.5.3), HOMER (v4.9), R custom scripts (a repository is provided).

For manuscripts utilizing custom algorithms or software that are central to the research but not yet described in published literature, software must be made available to editors/reviewers. We strongly encourage code deposition in a community repository (e.g. GitHub). See the Nature Research guidelines for submitting code & software for further information.

Data

Policy information about availability of data

All manuscripts must include a data availability statement. This statement should provide the following information, where applicable:

- Accession codes, unique identifiers, or web links for publicly available datasets
- A list of figures that have associated raw data
- A description of any restrictions on data availability

RADICL-seq and CAGE sequencing data were deposited in the GEO database with the accession code GSE132192.

Field-specific reporting

Please select the one below that is the best fit for your research. If you are not sure, read the appropriate sections before making your selection.

Life sciences study design

All studies must disclose on these points even when the disclosure is negative.

Sample size	Not used
Data exclusions	Not used
Replication	All RADICL-seq experiments were performed in duplicates or triplicates. High reproducibility for replicates from same experimental conditions was shown in Fig. 1c and Supplementary Fig. 5
Randomization	Not used
Blinding	Not used

Reporting for specific materials, systems and methods

We require information from authors about some types of materials, experimental systems and methods used in many studies. Here, indicate whether each material, system or method listed is relevant to your study. If you are not sure if a list item applies to your research, read the appropriate section before selecting a response.

Materials & experimental systems

n/a	Involved in the study	
☒	☐	Antibodies
☐	☒	Eukaryotic cell lines
☒	☐	Palaeontology
☒	☐	Animals and other organisms
☒	☐	Human research participants
☒	☐	Clinical data

Methods

n/a	Involved in the study	
☒	☐	ChIP-seq
☒	☐	Flow cytometry
☒	☐	MRI-based neuroimaging

Eukaryotic cell lines

Policy information about cell lines

Cell line source(s)	Mouse embryonic stem cells (mESCs) R08 purchased from RIKEN BioResource Center. Olineu cell line derived from mouse oligodendrocyte progenitor cells (mOPCs) obtained from the lab of the original publication (Jung M et al, 1995).
Authentication	mESCs and mOPCs were checked for morphology by microscope. Furthermore, from each cell type, RNA was extracted, converted to cDNA, specific expression markers were checked by quantitative PCR to confirm the cell type.
Mycoplasma contamination	mESCs and mOPCs were tested negative for mycoplasma contamination.
Commonly misidentified lines (See ICLAC register)	No commonly misidentified lines have been used.
