# Supplementary material for: RADICL-seq identifies general and cell type–specific principles of genome-wide RNA-chromatin interactions
Source: Nat Commun. 2020 Feb 24;11:1018. doi: 10.1038/s41467-020-14337-6 (PMC7039879; doi:10.1038/s41467-020-14337-6)
Supplement: Supplementary file 2 — Peer Review File [file 41467_2020_14337_MOESM2_ESM.pdf]

Editorial Note: This manuscript has been previously reviewed at another journal that is not operating a transparent peer review scheme. The manuscript was considered suitable for publication without further review at *Nature Communications.*
